# Surface-Enhanced Raman Spectroscopy in Breast Cancer Detection: A Bibliometric Review and Landscape of Global Trends

**DOI:** 10.3390/bios16090511

**Published:** 2026-09-10

**Authors:** Alitzel B. García-Hernández, Gethzemani M. Estrada-Villegas, Ana L. Gómez-Gómez, Ma. de la Paz Salgado-Cruz, Dana M. Cortez Landa

**Affiliations:** 1 Centro de Investigación en Química Aplicada (CIQA), Parque de Investigación e Innovación Tecnológica, Apodaca 66628, Nuevo León, Mexico; 2 Departamento de Ingeniería Mecatrónica, Universidad Politécnica de Apodaca, El Barrenal, Apodaca 66600, Nuevo León, Mexico; 3 Departamento de Ingeniería Bioquímica, Escuela Nacional de Ciencias Biológicas, Instituto Politécnico Nacional, U. P. Adolfo López Mateos, Ciudad de México 07738, Mexico

**Keywords:** SERS, biosensors, diagnostic, breast cancer, biofunctionalization, nanomaterials

## Abstract

Surface-Enhanced Raman Spectroscopy (SERS) has emerged as a powerful analytical platform for breast cancer (BC) detection, offering ultrasensitive, multiplexed, and label-free molecular recognition. However, despite the rapid expansion of the field, no prior study has combined quantitative bibliometric mapping with a cluster-validated technical and translational synthesis, limiting a comprehensive understanding of the field’s structure, evolution and clinical projection. In this review, a PRISMA-guided bibliometric analysis was conducted; 199 articles on SERS-based BC detection (2016–2025) were retrieved from SCOPUS, Web of Science and Google Scholar, mapping publication trends, keyword co-occurrence networks (VOSviewer), and Multiple Correspondence Analysis (MCA) with hierarchical clustering on principal components. The results reveal sustained growth in scientific output, led by Asia, North America, and Europe. The 20 most-cited articles (271 citations maximum) showed a shift from substrate optimization toward AI-assisted liquid biopsy platforms. MCA identified five clusters, corroborated by the co-occurrence network: (1) nanostructured platforms for diagnosis; (2) biofunctionalization strategies; (3) liquid biopsy approaches targeting exosomes, circulating tumor cells, and alternative biofluids; (4) diagnostic interpretation based on chemometrics, machine learning (ML) and artificial intelligence (AI); and (5) translational achievements in preclinical and clinical studies. Each cluster was anchored by a technical sub-analysis of its landmark studies, an integration largely absent from prior SERS reviews. This framework clarifies the field’s trajectory and positions SERS as a key technology for non-invasive, personalized diagnostics in precision oncology.

## 1. Introduction

Breast cancer (BC) is the most frequently diagnosed malignancy among women worldwide and remains a leading cause of cancer-related mortality [1]. Current epidemiological data report more than 2.4 million new cases annually globally, accounting for nearly 19% of all cancer deaths in women [2]. Despite significant advances in screening and therapeutic strategies, the global problem of BC continues to rise, underscoring the urgent need for diagnostic technologies that are sensitive, accurate, and non-invasive, capable of improving early detection and guiding personalized treatment.

Conventional diagnostic methods, including mammography, ultrasound, magnetic resonance imaging (MRI), and histopathological biopsy, have long served as the clinical standard. While these approaches are effective, they are constrained by radiation exposure, invasiveness, high costs, and reduced sensitivity in certain patient populations, particularly women with dense breast tissue [3,4,5]. Complementary biochemical assays targeting biomarkers such as HER2, CA15-3, estrogen receptor (ER), and progesterone receptor (PR) provide valuable molecular insights [6]. However, these assays often lack sufficient sensitivity and specificity for early-stage disease, limiting their utility as individual diagnostic modalities capable of bridging the gap between imaging and molecular profiling.

Raman spectroscopy offers one such approach; it is a non-destructive vibrational spectroscopy based on inelastic scattering of monochromatic light, in which the energy change between incident and scattered photons corresponds to specific molecular vibrational modes. This produces a highly specific “molecular fingerprint” capable of identifying biomolecules such as proteins, lipids, and nucleic acids without the need for labeling. However, the Raman scattering cross-section is intrinsically small, resulting in a very low ratio of scattering to excitation photons. In practice, only about one in 10^6^–10^8^ incident photons undergo Raman scattering [7,8]. This weak signal is further compounded by a strong autofluorescence background in biological matrices, which together limit the sensitivity of conventional Raman spectroscopy for the detection of trace-level analytes in complex biofluids.

To overcome this limitation, SERS employs an active plasmonic substrate, typically composed of noble-metal nanostructures such as gold (Au) nanoparticles (NPs, AuNPs) or silver (Ag) NPs (AgNPs), that amplify the Raman signal by several orders of magnitude, in some cases achieving up to 10^14^-fold enhancement [9]. This enhancement arises from two complementary mechanisms. The electromagnetic mechanism (EM) accounts for the main contribution and originates from the excitation of localized surface plasmon resonance (LSPR) on the nanostructure surface. In this mechanism, when analyte molecules are positioned within nanoscale gaps or sharp geometric features (“hotspots”), the local electromagnetic field intensifies dramatically, amplifying both incident and scattered light. The chemical mechanism (CM), although generally of smaller magnitude, arises from charge-transfer interactions between the adsorbed analyte and the metal surface that modify the polarizability of the adsorbed molecule and further contribute to signal enhancement. The synergistic interaction of EM and CM defines the overall enhancement factor (EF) of a given SERS substrate, and optimizing this interaction through nanostructure design remains a central strategy to improve detection sensitivity. Together, fluorescence quenching, diffraction spatial resolution, and EFs sufficient to enable single-molecule detection have positioned SERS as a substantially more sensitive alternative to conventional Raman spectroscopy for biomarker analysis for BC detection [10].

A wide spectrum of biomarkers has been investigated using SERS, including proteins (HER2, EpCAM, CD44) [11,12,13,14], nucleic acids (microRNAs (miRNAs), telomerase activity) [15,16,17], extracellular vesicles (exosomes) [18,19,20], and metabolites. These targets have been analyzed across diverse sample types such as tissues [21], blood [22], serum [23], plasma [24], urine [25], tears [26] and nipple discharge [27], enabling both invasive and non-invasive diagnostic strategies. Recent advances have further integrated SERS with microfluidic chips [28,29], aptamer-based biosensors, ratiometric probes [30,31], ML [32,33,34,35] and AI algorithms [36,37,38], facilitating high sensitivity, multiplexing [34,39,40,41,42], and real-time clinical applications [43].

**Figure 1:**
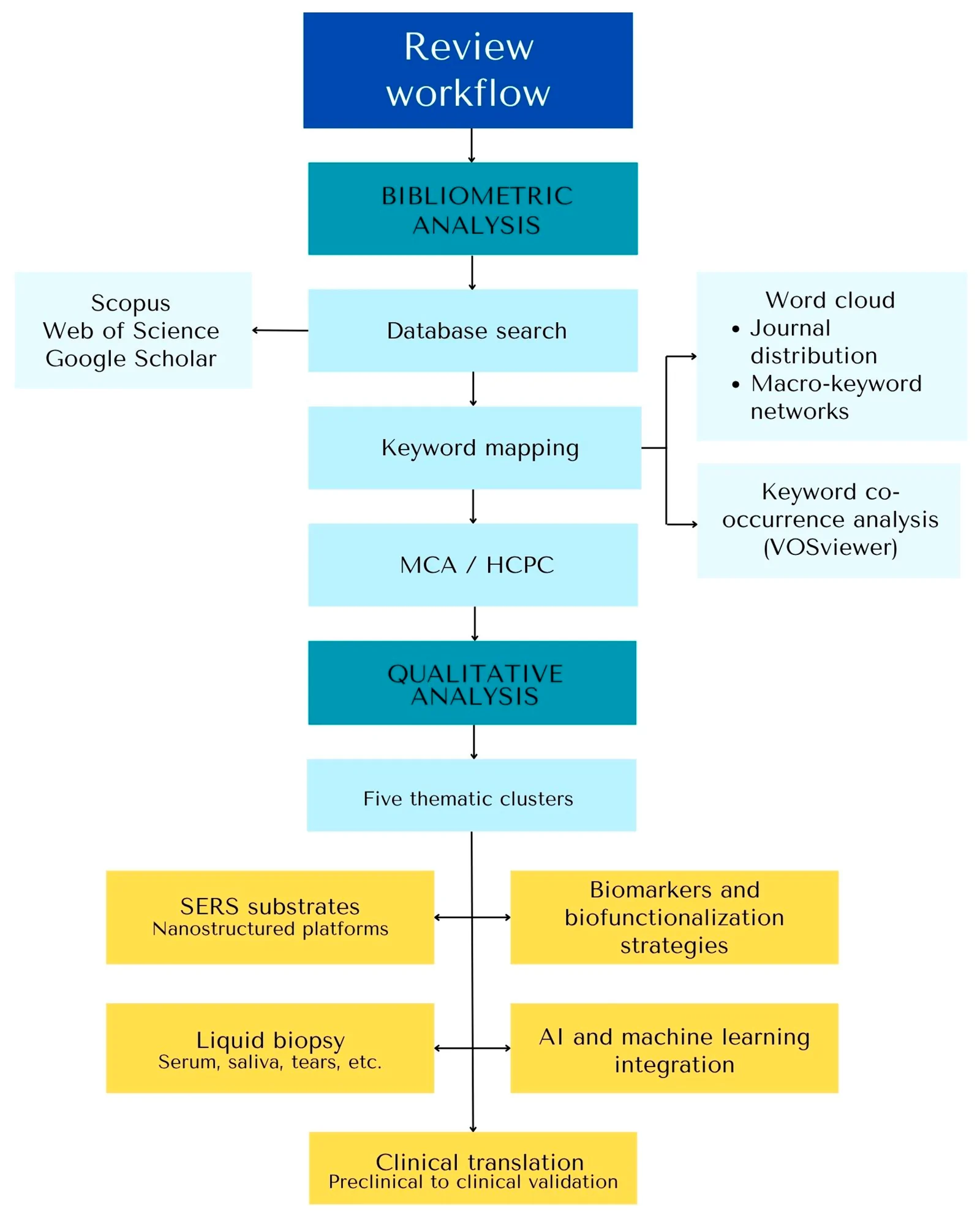
Review workflow for SERS-based breast cancer detection: bibliometric analysis, keyword mapping, MCA/HCPC clustering, and thematic domains.

In this context, our review provides an integrated, evidence-based bibliometric synthesis of global research on SERS breast cancer detection. As illustrated in Figure 1, the review follows a structured workflow combining bibliometric analysis, keyword mapping, and Multiple Correspondence Analysis with hierarchical clustering on principal components (HCPCs) to identify five core thematic domains, which are then examined through a critical, cluster-level technical and clinical synthesis. Within this framework, we map publication trends, geographic distribution, highly influential studies, and emerging research directions; critically examine the evolution of plasmonic nanoarchitectures and biosensor designs and their relationship to conventional Raman spectroscopy; and evaluate their application across diverse biomarkers and biological samples. This integrated framework aims to clarify the current maturity of the field and to identify the methodological, technological, and regulatory priorities required to advance SERS from experimental breast cancer models toward reproducible, clinically applicable diagnostic tools.

Several reviews have addressed SERS in the context of cancer diagnostics, liquid biopsy, biomarker detection, bioimaging, microfluidics, and theranostics [44,45,46,47,48,49,50,51]. However, a structured comparison of the relevant literature reveals that only a limited number of reviews focus specifically on breast cancer, and even fewer consider SERS as their primary analytical approach [44,45,46,47]. Reviews dedicated to breast cancer have largely concentrated on liquid biopsy biomarkers, onco-miRNAs, or plasmonic biosensors. In contrast, broader reviews on SERS explore multiple diseases, biomarker types, material systems, or therapeutic applications [48,49,52]. While these contributions provide valuable technical perspectives, they generally organize evidence according to biomarkers, detection modalities, or applications, without systematically linking nanoarchitecture design to analytical performance, methodological robustness, and clinical translation.

Table S1 in the Supplementary Material summarizes these contributions. Nevertheless, none of the identified reviews integrate bibliometric mapping with a functional comparison of SERS nanoarchitectures and a critical evaluation of validation quality, reproducibility, machine learning, and clinical feasibility. The present review addresses this gap by systematically linking nanoarchitecture-dependent amplification and detection functions to biological targets, analytical performance, evidence quality, and translational viability.

## 2. Methods

### 2.1. Data Collection

Data were retrieved from three databases: Scopus, consulted on 3 March 2026, complemented by Web of Science (WoS) and Google Scholar (GS) on 20 August 2026, covering publications from 2016 to 2025 (Tables S2–S4 in the Supplementary Material). A structured search strategy was designed using Boolean operators (AND, OR) and the keywords breast cancer, SERS, diagno*, and detection. The search was restricted to research articles published in English.

For Scopus, the final query was (TITLE-ABS-KEY (breast cancer)) AND (TITLE-ABS-KEY (SERS RAMAN)) AND (TITLE-ABS-KEY (DIAGNO* OR DETECTION)) AND PUBYEAR > 2015 AND PUBYEAR < 2027 AND (LIMIT-TO (DOCTYPE, “ar”)) AND (LIMIT-TO (LANGUAGE, “English”)), constructed using the “Combine queries” tool. This search yielded 231 records, from which bibliographic information, abstracts, and author/index keywords were exported in CSV format for subsequent analysis. After applying predefined inclusion and exclusion criteria, 196 articles were retained for qualitative synthesis and bibliometric analysis.

In GS, the advanced search tool was applied with the terms breast cancer, SERS, diagnosis, diagnostic, and detection, using “SERS Raman” as an exact phrase. The query returned 29 documents, of which eight met the inclusion criteria; however, four were duplicates of Scopus records, and one was repeated in the same search.

For WoS, the “combine queries” tool was used with the following structure: #1 (TI = breast cancer), #2 (TI = SERS), #3 (TI = detection), #4 (TI = diagno*), #5 (#3 OR #4). The final query was (#1 AND #2 AND #5) AND (DT == “ARTICLE” AND LA == “ENGLISH”), yielding 34 documents, including 28 duplicates from Scopus and 6 unique articles.

**Figure 2:**
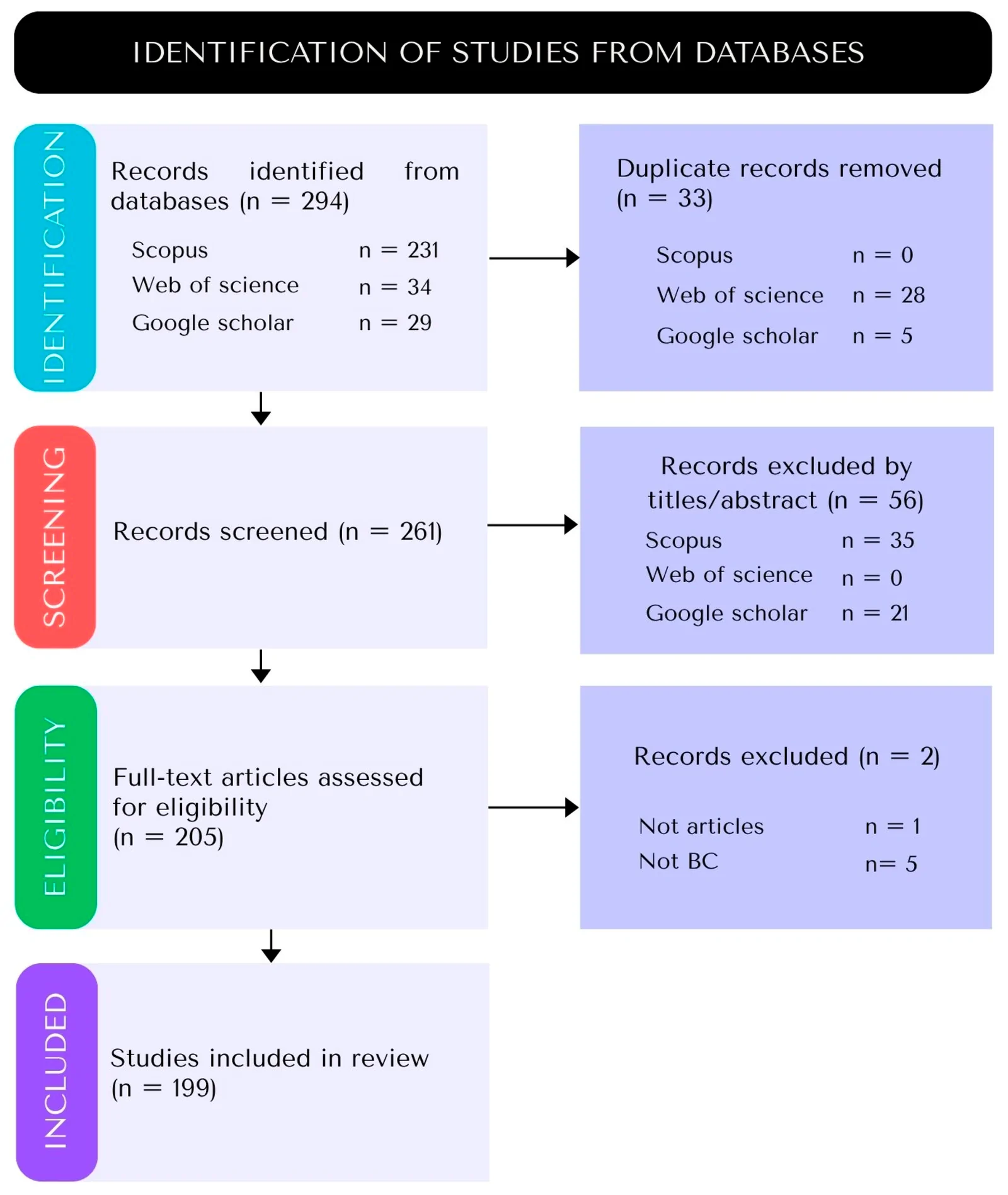
PRISMA-based study selection for SERS breast cancer detection review.

To ensure transparency, reproducibility and methodological rigor, the study selection process followed the four PRISMA phases—identification, screening, eligibility, and inclusion—as observed in Figure 2.

### 2.2. Data Processing for Performance Metrics

OriginPro 2021 (OriginLab Corporation, Northampton, MA, USA) was employed to process numerical data, including the geographical distribution of scientific production (number of papers published per country and per year). For this evaluation, the aim was to capture the general trend of the data; therefore, 227 original articles (after excluding reviews) from the CSV file retrieved from the Scopus search were used as the primary dataset. Linear regression analysis was applied to fit the publication trend, yielding the equation of the form y=mx+b, where *y* represents the response variable (number of published papers), *x* denotes the year, *m* corresponds to the slope (describing the rate of increase in publications), and *b* is the intercept. This approach allowed quantification of the temporal trend and assessment of the growth trajectory of research output.

To assess academic prominence and get a comprehensive overview of the research landscape, the top 20 most-cited articles on the topic were systematically identified for targeted analysis. Core bibliographic data, including titles, authors, publication years, and journals, were extracted from the Scopus database, along with cumulative citation metrics. Additionally, to evaluate the institutional and academic reach of these landmark studies, the respective Journal Impact Factors (JIFs) were cross-referenced using data retrieved from the official publisher platforms.

### 2.3. Bibliographic Mapping and Network Visualization Settings (Word Cloud Mapping and Keyword Co-Occurrence Network Analysis)

Word clouds of keywords and journals were generated using the Word*It*Out online platform (WordItOut, Enideo, Antwerp, Belgium; available at worditout.com). For the keyword cloud, only terms with a frequency greater than three occurrences (>3) were included, whereas for the journal cloud, terms with more than one occurrence (≥ 1) were considered. In both cases, the “minor” option in the “difference in sizes of words” tool was applied to adjust the relative scaling of word sizes.

Keyword co-occurrence analysis was done using VOSviewer (version 1.6.20, Leiden University, Leiden, The Netherlands), a widely recognized tool for bibliometric mapping. The co-occurrence network was constructed based on keyword frequency (author and index keywords) extracted from the CSV export file, applying a minimum occurrence threshold of ≥5 to reduce noise and enhance interpretability. The resulting network was visualized using the association strength normalization method, which facilitated the identification of thematic clusters and their interrelationships. Cluster interpretation was performed according to keyword composition, and temporal overlay visualization was employed to highlight emerging research trends within the field.

Bibliometric quantification (word cloud mapping, keyword co-occurrence mapping and Multiple Correspondence Analysis (MCA)) was restricted to the Scopus-derived database to preserve metadata consistency, given that Scopus provides standardized, exportable author and index keyword fields required for these analyses. However, records retrieved from Scopus, WoS and GS were incorporated into the qualitative cluster thematic sections to ensure structural artifacts arising from heterogeneous metadata schemas across databases, methodological consistency and a broader representation of the scientific landscape related to SERS-based breast cancer detection.

### 2.4. Multiple Correspondence Analysis (MCA)

MCA was employed to explore relationships among categorical variables, as it is particularly suited for binary datasets and latent structure identification based on chi-square distances. The analysis used a curated set of keywords derived from selected articles. Preprocessing was performed in Power Query (Microsoft Excel, V. 2606, Microsoft Corporation, Redmond, WA, USA) to merge synonyms and standardize terminology (Table S5 in the Supplementary Material), while terms appearing in <4 documents were excluded to reduce sparsity and improve robustness (Table S6 in the Supplementary Material).

A binary presence–absence matrix was constructed (Table S7 in the Supplementary Material), with rows representing unique publications and columns representing keywords (1 = presence; 0 = absence). Computations were carried out in RStudio (version 4.x, Posit Team, Boston, MA, USA), utilizing the ‘FactorMineR’ package for multivariate computational analyses, following the methodological framework of [53] (Table S8 in the Supplementary Material). Dimensionality reduction was achieved by extracting principal dimensions that maximize explained inertia. The optimal number of retained dimensions was determined using eigenvalues, explained inertia, scree plot inspection, and Benzécri’s correction to adjust for artificial inertia inflation.

Interpretation was based on keyword contributions and spatial distribution in the factorial plane, with results visualized through two-dimensional factor maps to identify thematic groupings. To complete the spatial projection, hierarchical clustering on principal components (HCPCs) further classified publications and keywords into homogeneous clusters. Then, MCA patterns were contrasted with co-occurrence network analysis, providing complementary insights into the structure and evolution of the research field.

Finally, to qualitatively ground and concretize each thematic domain derived from the HCPC, a targeted sub-analysis was conducted within each cluster. The bibliographic metadata and core insights of these landmark papers were systematically summarized in comparative tables and critically analyzed in Section 3.5, Section 3.6, Section 3.7, Section 3.8 and Section 3.9.

## 3. Results and Discussion

### 3.1. Bibliometric Review and Macro-Trends Analysis

#### 3.1.1. Geographical Distribution of Scientific Production

**Figure 3:**
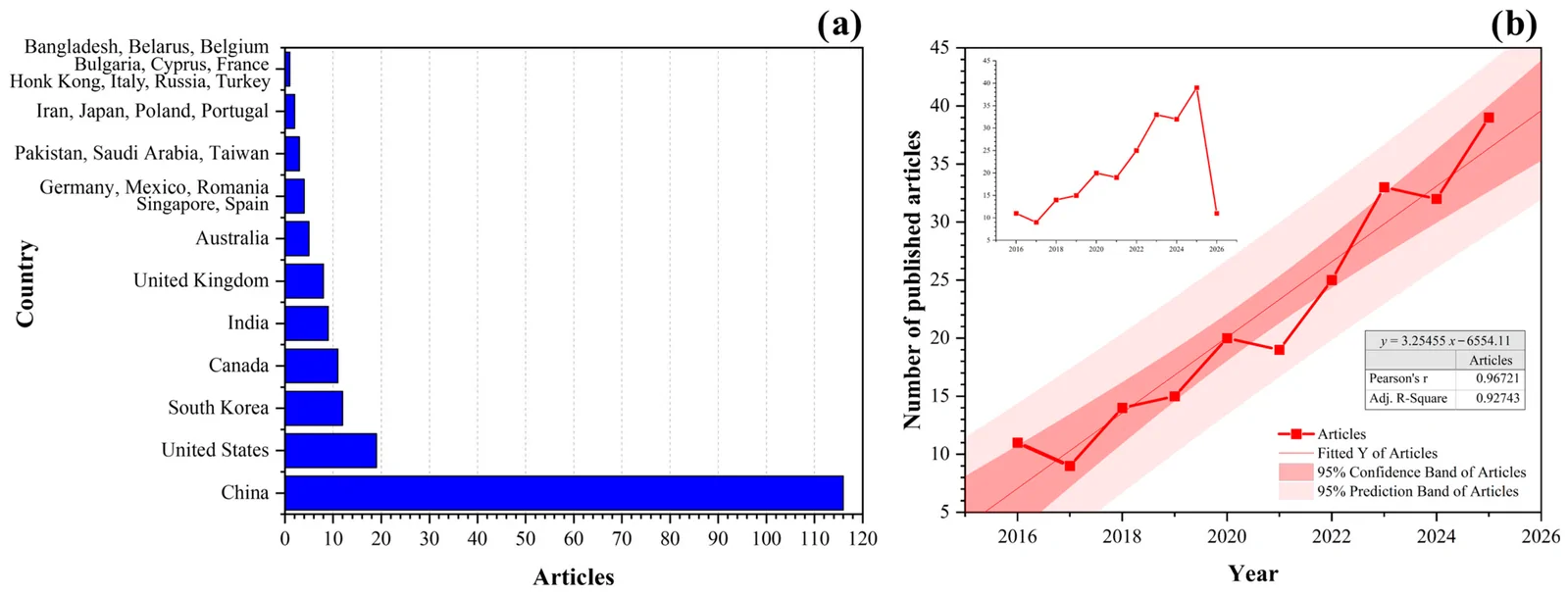
(**a**) Number of articles published per country from 2016 to 2025; (**b**) fitted linear trend for annual publications up to 2025; the inset displays the total number of articles published until 6 March 2026.

Figure 3a illustrates the global distribution of research articles focusing on SERS for breast cancer detection. The bibliometric analysis reveals a pronounced geographic concentration of research activity, with a few key countries driving most of the advancements. China emerges as the main contributor, accounting for 116 published articles (51.10% of the total). The United States ranks second with 19 articles (8.37%), followed by South Korea (12, 5.28%), Canada (11, 4.84%), and India (9, 3.96%). Conversely, nations such as the United Kingdom (8, 3.52%), Australia (5, 2.20%), and various other European, Middle Eastern, and Asian countries exhibit moderate to low publication volumes, collectively representing 20.70% of global production. This distribution suggests that SERS-related research has achieved broad international participation. Nevertheless, the Scopus results show that China and the United States account for a large share of the publications, indicating their leading role in the generation of scientific output within this research domain.

#### 3.1.2. Annual Publication Trends and Growth (2016–2025)

The evolutionary trajectory of the scientific literature in the SERS and breast cancer domain from 2016 to 2025 is presented in Figure 3b, while the inset, as a reference, presents the overall production until 6 March 2026. In Figure 3b, the data exhibits a pronounced, sustained ascending trend in annual publication volume over the last decade, reflecting an accelerating global interest in this spectroscopic technique. Starting with 11 publications in 2016, the research output has more than tripled, reaching 39 articles in 2025. Linear regression analysis applied to this data produced a strong positive slope, yielding an equation indicative of robust and continuous growth in research productivity. The high adjusted R-square (R^2^ = 0.92743) and Pearson’s correlation coefficient (*r* = 0.96721) demonstrate a highly positive linear relationship between temporal progression and research publications. This pattern reflects a sustained increase in scientific output over the study period, suggesting growing research interest and continued activity in the field. The narrow 95% confidence and prediction bands further support the stability and statistical predictability of this publication trend. However, these findings should be interpreted as evidence of publication growth rather than indicators of scientific maturity, methodological robustness, or readiness for clinical implementation, as such aspects were not formally assessed in the present study.

Despite minor inter-annual fluctuations, notably the slight declines observed in 2017 and 2021, the overall trend demonstrates a sustained increase in research activity over time. This continued growth reflects ongoing scientific interest in the application of SERS for BC research and highlights its exploration as a potential analytical approach for molecular characterization and detection-related studies. However, publication growth alone should not be interpreted as evidence of scientific maturity, clinical adoption, or diagnostic effectiveness, as these aspects were not formally assessed in the present study.

#### 3.1.3. Analysis of Highly Cited Documents

The analysis of highly cited documents serves as a robust indicator for identifying intellectual milestones, novel methodologies, and shifting paradigms that shape a scientific discipline. By evaluating the top 20 most-cited articles within the domain of SERS applied to breast cancer diagnostics (compiled between 2016 and 2025), a clear transition from fundamental substrate optimization to highly sophisticated clinical research is observed (Table 1).

The dataset reveals an entirely geographic concentration of high-impact research, representing the national production trends identified in the previous analysis (Figure 3a). Of the top 20 significant papers, China accounts for 16 publications, followed by South Korea (2) and the United States (2). Notably, the study by Shin et al. (2023) published in *Nature Communications* has garnered attention owing to its remarkable citation velocity, accumulating 271 citations in a very short temporal window [38]. This rapid citation highlights an immediate global focus on non-invasive technologies and universal cancer screening platforms.

The distribution of these papers across high-impact journals reinforces the field’s interdisciplinary nature. These publications are classified across distinct scientific subareas such as multidisciplinary (e.g., *Nature Communications*, *Angewandte Chemie International Edition*), nanotechnology and materials science (e.g., *ACS Nano*, *Advanced Functional Materials*, *Nano Letters*, *Small*), biosensing (e.g., *Biosensors and Bioelectronics*, *Talanta*, *ACS Sensors*), and oncology (e.g., *Cancer Research*).

This publishing distribution demonstrates that while the foundational physics and material validation of SERS remain deeply related to analytical chemistry and materials science platforms (with Journal Impact Factor, JIF, ranging from 1.8 to 19.0), clinical validation and therapeutic translation are penetrating top-level medical and multi-disciplinary literature. This advanced growth reinforces SERS as a leading candidate for next-generation oncological diagnostics.

Regarding the academic structure of these top-cited works, the literature can be classified into five specific research fronts, which directly validate and anchor the clusters observed in the VOSviewer and MCA maps (Section 3.3 and Section 3.4, respectively):

1. Optimization of Physicochemical Properties in the Plasmonic Surfaces: A significant number of the highly cited literature is dedicated to overcoming the traditional SERS limitations of signal reproducibility and intensity through advanced substrate engineering. The top-cited papers demonstrate that optimizing the morphology, composition, and spatial arrangement of nanomaterials is critical to creating dense, reproducible electromagnetic “hotspots”.
  Researchers have innovated from simple spherical nanoparticles toward complex, high-aspect-ratio geometries. For instance, Jimenez De Aberasturi et al. (2016) presented a branched morphology of gold nanostars to achieve single-cell sensitivity [54]. Similarly, Eom et al. (2017) demonstrated that sharp-tip geometries in gold nanobipyramids and engineered “nanogap-rich” structures in gold nanowires provide superior localized surface plasmon resonance (LSPR), driving detection limits down to the attomolar level [10].
  However, to introduce multifunctionality and enhance chemical stability, authors are synthesizing multicomponent substrates, such as hybrid substrates. Pang et al. (2016) developed a dual-function Fe_3_O_4_@Ag core–shell system, utilizing the magnetic iron core for target enrichment and the silver shell for strong SERS enhancement [17]. Other high-impact configurations combine plasmonic metals with carbonaceous materials, such as the Au@Ag/graphene oxide platforms engineered by Q. Zhang et al. (2023) to suppress background noise and improve structural reproducibility [55].
  Beyond individual nanoparticles, improving the surface properties has emerged as a key trend. Ma et al. (2020) achieved significant signal amplification by coupling silver nanoparticles with a porous silicon Bragg reflector, successfully exploiting the synergistic effects of photonic bandgap structures and plasmonics [56]. On the other hand, Lin et al. (2021) optimized the surface chemistry by fabricating super-hydrophobic SERS substrates, allowing droplets of clinical serum to concentrate perfectly on targeted zones, which drastically minimized signal variability during automated screening [57].
  Collectively, these highly cited works establish that the rational design of a substrate’s physicochemical properties is a fundamental enabler for achieving the ultra-low limits of detection necessary to detect breast cancer biomarkers in real-world clinical samples.
2. Multiplexed Nanoprobe and Aptamer Engineering: Achieving the molecular specificity required to differentiate breast cancer subtypes or heterogeneous cell populations is a recurring bottleneck. High-impact works have addressed this through sophisticated surface biochemical functionalization. Choi et al. (2020) successfully employed multiplexed targeting using a combination of anti-EpCAM, anti-ErbB2, and anti-CD44 functionalized nanospheres [58]. Similarly, Q. Zhang et al. (2023) introduced dual-aptamer ratiometric SERS biosensors, proving that multi-target recognition significantly suppresses false-positive rates and drastically reduces signal variability [55].
3. The Liquid Biopsy and Exosomal Frontier: Exosomes and circulating microRNAs (miRNAs) represent the most prominent sample matrices among the top-cited papers. Studies like Shin et al. (2023) (271 citations) [38], Lee et al. (2019) (233 citations) [59], and Xie et al. (2022) (154 citations) [19] underline a major paradigm shift away from invasive, solid tissue biopsies toward blood-based diagnostics. The literature demonstrates that isolating, capturing, and profiling serum- or plasma-derived exosomes via advanced plasmonic architectures offers an ultra-sensitive, non-invasive way for early-stage breast cancer screening and postoperative recurrence monitoring.
4. Artificial Intelligence and Chemometric Convergence: The integration of advanced computational workflows is a defining characteristic of the most influential papers. Innovative studies, such as Lin et al. (2021) using deep learning on super-hydrophobic SERS substrates [57], and Diao et al. (2023) utilizing machine learning for label-free exosome classification, demonstrate that raw spectral fingerprints are no longer analyzed in isolation [33]. The consensus among highly cited authors is that AI and chemometrics are mandatory components to resolve the inherent molecular complexity and overlapping spectral bands typical of clinical human samples.
5. Clinical Translation: The clinical translation of SERS is extending into the operating room and expanding into alternative diagnostic fluids. Wang et al. (2017) in *Cancer Research* (98 citations) validated the topical application of SERS nanoparticles for real-time intraoperative guidance during lumpectomies, allowing surgeons to visualize tumor margins [60]. On the other hand, Kim et al. (2020) open another path by using human tears as a completely non-invasive biofluid, demonstrating that SERS can successfully discriminate metabolite variations in breast cancer patients using point-of-care setups [26].

To conduct a comprehensive bibliometric analysis, the articles listed in Table 1 were evaluated based on their quality, validation studies performed, risk of bias, reproducibility, and sample size, as summarized in Table 2. A discrepancy was observed between bibliometric influence and the limited clinical validation of SERS platforms. Within this subsection, technical and preclinical studies reported high sensitivity, in some cases low limits of detection (LOD), and notable improvements in reproducibility. For instance, Lin et al. [57] and Lee et al. [59] achieved relative standard deviations (RSDs) of 2.3–4.65% by controlling the coffee-ring effect and ensuring homogeneous hotspot distribution. Likewise, Pang et al. [17] and Lee et al. [59] attained femtomolar and attomolar LODs, respectively. However, these results co-existed with reduced effective sample sizes, retrospective designs, potential spectral pseudo-replication, predominantly internal validation, and limited assessment of reproducibility across batches, operators, and instruments. Moreover, EFs, LODs, and RSD values are inherently specific to each platform and describe its analytical performance, but do not, in themselves, constitute evidence of methodological quality, diagnostic accuracy, or clinical utility. Even the studies with the largest cohorts [38,57] used test sets drawn from the same selection framework, meaning their results do not represent fully independent external validation.

This gap was most evident in studies employing artificial intelligence or multivariate classification approaches. In several works, the large number of spectra contrasted with a small number of participants, cell lines, or independent biological units, increasing the risk of pseudo-replication and information leakage. This was particularly apparent in Xie et al. [19], who generated 8265 spectra from four cell lines, whereas Kim et al. [26] produced multiple spectra from only 10 participants. When spectra originating from the same biological unit can be distributed across both training and test sets, there is a risk of pseudo-replication and information leakage, which may overestimate model accuracy. The number of spectra should therefore not be interpreted as equivalent to the effective sample size.

The studies by Shin et al. [38] and Lin et al. [57] represented the most extensive and methodologically robust evaluations, enrolling 753 and 695 participants, respectively, and employing test sets separated at the participant level. Nevertheless, these test sets originated from the same retrospective selection framework, corresponding to held-out internal evaluations rather than truly independent external validation. Among the remaining human studies, small sample sizes, internal cross-validation, and extreme case–control designs predominated. Consequently, the high accuracy, sensitivity, specificity, or area under the receiver operating characteristic curve (AUROC) values reported should be interpreted as exploratory findings, particularly when not accompanied by confidence intervals, calibration assessment, or geographic, temporal, or prospective validation.

Taken together, Table 2 also shows that studies involving human samples did not necessarily reach the same level of evidence. Some works demonstrated only analytical feasibility using a small number of clinical samples, such as the two-sample study by Zheng et al. [61], while others conducted exploratory evaluations involving 10–60 participants [26,33,55,56,59,62]. Furthermore, the exclusion of mixed tissue regions in Wang et al. [60] and the predominance of retrospective two-gate designs increased the risk of overly optimistic estimates and limited the representativeness of real-world clinical scenarios.

In addition, cellular, animal, and theranostic studies [10,17,30,54,58,59,63,64,65] provided important evidence regarding sensitivity, selectivity, stability, and detection mechanisms, but should not be interpreted as diagnostic validations. Their value lies in demonstrating technical or preclinical feasibility rather than establishing sensitivity, specificity, or generalizability in patients.

Overall, Table 2 demonstrates that favorable enhancement factors, limits of detection, and algorithmic accuracies did not necessarily correspond to greater clinical robustness. The available evidence primarily supports the analytical feasibility and exploratory diagnostic potential of SERS. Clinical implementation will require larger effective sample sizes, participant-level data partitioning, prospective and external cohorts, representative populations, confidence intervals, and a more comprehensive assessment of reproducibility across batches, operators, instruments, and centers.

In summary, the analysis of the 20 most cited papers confirms that SERS-based breast cancer research has evolved from an isolated material science pursuit into a mature, highly collaborative translational discipline, covering five interconnected research fronts: plasmonic substrate optimization, multiplexed biofunctionalization, liquid biopsy platforms, AI-assisted spectral interpretation, and clinical translation. However, the accompanying critical judgment reveals that bibliometric prominence does not consistently align with the rigor of clinical validation. While several high-impact studies achieved femtomolar to attomolar limits of detection and RSD values below 5%, reflecting genuine advances in analytical performance, most relied on limited effective sample sizes, internally validated test sets, and, in AI-assisted studies, spectra-to-participant ratios that raise concerns of pseudo-replication and information leakage. Even the two most extensively evaluated cohorts [38,57] were tested on internal rather than truly independent external datasets. Accordingly, the field’s most-cited achievements should be regarded as strong evidence of analytical feasibility and exploratory diagnostic potential, rather than established clinical validity. Closing this gap will require larger, participant-level-partitioned cohorts, prospective and external validation, and standardized reproducibility testing across batches, operators, and instruments, an effort increasingly supported by the integration of targeted surface chemistry (aptamers/antibodies) and computational intelligence (machine learning).

To transition from this analysis of the high-impact literature to a complete, systemic overview of the entire dataset, the subsequent sections map the macro-level structures of the field through three bibliometric lenses: journal analysis and keyword network mapping (Section 3.2), keyword co-occurrence analysis (Section 3.3), and Multiple Correspondence Analysis (Section 3.4).

**Table 1:** The twenty most cited documents between 2016 and 2025 from Scopus database.

Biomarker	Sample	Type of Sensor or Substrate	Aim	Main Findings	Journal and IF	TC Country (Year)	Reference
Exosomes	Plasma	AuNPs coated on the APTES-functionalized cover glass	Establish a single diagnostic platform capable of detecting multiple cancer types at early stages using exosome profiling enhanced by SERS and AI.	Exosome-derived SERS spectra, when analyzed with AI, achieved over 95% accuracy in distinguishing six different cancers from plasma samples, demonstrating the feasibility of a universal, non-invasive diagnostic test. Highlighted potential for clinical translation of SERS-based diagnostics.	Nature Communications 15.7	271 South Korea (2023)	[38]
Exosomes miRNA	Serum	Plasmonic head-flocked gold nanopillars	Develop a highly sensitive and specific SERS-based sensor for detecting exosomal microRNAs (miRNAs) as biomarkers for breast cancer.	Developed SERS sensor achieved femtomolar-level sensitivity and high specificity for exosomes, with accurate breast cancer diagnosis.	Small 13.8	233 South Korea (2019)	[59]
microRNAs	RNA extract	Fe_3_O_4_@Ag magnetic nanoparticles: core of (magnetic iron oxide) for easy separation and enrichment and shell of Ag for strong plasmonic enhancement in SERS	Design a highly sensitive SERS-based detection platform for microRNAs (miRNAs) in cancer cells through the integration of magnetic nanoparticles (Fe_3_O_4_@Ag) with duplex-specific nuclease (DSN) signal amplification for improved sensitivity and specificity.	Dual-function nanoparticle system (Fe_3_O_4_@Ag), combined with duplex-specific nuclease signal amplification, enabled femtomolar detection (0.3 fM) of intracellular miRNAs, providing a powerful tool for early cancer diagnosis and intracellular biomarker analysis.	Biosensors and Bioelectronics 12.6	199 China (2016)	[17]
Breast cancer cell lines		Gold nanostars (AuNSs) prestabilized with PEG4-mbt	Create SERS-encoded gold nanostars as multiplexed probes for cell discrimination and identification between different cell populations.	Branched morphology of gold nanostars, encoded with distinct Raman reporters, allowed single-cell sensitivity and simultaneous identification of multiple cell types, highlighting their potential for multiplexed biomedical diagnostics.	Chemistry Materials 10.4	169 United States (2016)	[54]
Synthetic DNA sequences		Hybrid nanostructured biosensor: gold-disk electrode (AuDE) was coated with reduced graphene oxide (rGO), decorated with plasmonic gold-coated Fe_2_Ni@Au magnetic nanoparticles functionalized with double-stranded DNA (dsDNA)	Develop and validate a dual-mode biosensor that integrates SERS and electrochemical detection for studying anticancer drug-DNA interactions.	Nanostructured SERS-electrochemical biosensors can sensitively and specifically detect interactions between anticancer drugs and DNA. The system provided complementary information on binding strength and molecular changes. The biosensor achieved nanomolar sensitivity (LOD: 8 µg/mL), offering a powerful platform for drug screening and mechanistic studies. This dual-mode approach highlighted the potential of nanostructured biosensors in pharmacological research and personalized medicine.	Biosensors and Bioelectronics 12.6	167 China (2016)	[65]
Exosomes	Serum	AuNSs	Establish a label-free, AI-assisted SERS platform for breast cancer diagnosis and postoperative monitoring using serum-derived exosomes.	Label-free SERS profiling of serum exosomes, combined with artificial intelligence, can accurately diagnose breast cancer and assess postoperative outcomes. Gold nanostructured substrates provided reproducible Raman fingerprints of exosomes, which were then classified by AI algorithms to distinguish cancer patients from healthy controls. The approach proved sensitive, non-invasive, and capable of monitoring treatment response, underscoring its potential for clinical translation in breast cancer diagnostics.	Nano Letters 11.3	154 China (2022)	[19]
Anti-epithelial cell adhesion molecule (EpCAM) Anti-erythroblastic oncogene B2 (ErbB2) Anti-cluster of differentiation (CD44)		PEGylated Ag-Au hollow nanospheres	Investigate a SERS-based biosensor platform capable of detecting multiple cancer biomarkers with high sensitivity and specificity.	Nanostructured SERS biosensors can achieve ultrasensitive and multiplexed detection of cancer biomarkers at femtomolar concentrations. By integrating gold and silver nanostructures with Raman reporter probes, the system provided strong signal enhancement and specificity, enabling simultaneous monitoring of multiple biomarkers in cancer cells. This multiplexing capability represents a significant advance in cancer diagnostics, offering a powerful tool for early detection and personalized medicine.	Biosensors and Bioelectronics 12.6	141 China (2020)	[58]
Metabolites and molecular signatures in tears	Human tears from breast cancer patients	Gold-decorated, hexagonal-close-packed polystyrene (Au/HCP-PS) nanosphere monolayer	Present a strategy for highly sensitive detection and quantification using tears as novel diagnostic biofluids for breast cancer non-invasive identification.	Label-free SERS biosensors can successfully detect breast cancer using human tear samples. Gold nanostructured substrates provided reproducible Raman fingerprints of tear metabolites, enabling discrimination between cancer patients and healthy controls. The approach was non-invasive, rapid, and sensitive, highlighting tears as a promising biofluid for cancer diagnostics and positioning SERS as a powerful tool for point-of-care applications.	ACS Applied Materials and Interfaces 9.5	133 China (2020)	[26]
Hydrogen peroxide		Core–satellite nanostructures: Core: Gold nanorods (photoacoustic-active). Satellites: Silver nanoparticles decorated with Raman reporters	Design a dual-mode nanoprobe capable of quantitatively detecting hydrogen peroxide in biological systems. By combining SERS and photoacoustic imaging in a single core–satellite nanostructure.	Dual ratiometric core–satellite nanoprobe can quantitatively visualize hydrogen peroxide in cancer and inflammation. Gold nanorods provided strong photoacoustic signals, while silver nanoparticle satellites carried Raman reporters that responded to H_2_O_2_, enabling ratiometric SERS detection. The system achieved nanomolar sensitivity, successfully monitored ROS levels in cancer cell lines, and visualized H_2_O_2_ distribution in tumor-bearing mice. This dual-mode approach provided complementary molecular and imaging information, advancing precision diagnostics of oxidative stress in disease.	Angewandte Chemie International Edition 16.9	132 China (2021)	[30]
Tissue and serum samples		Porous silicon Bragg reflector (PSBR) combined with AgNPs	Validate a PSBR-SERS biosensor for breast cancer detection, leveraging the synergistic effects of porous silicon photonic structures and silver nanoparticles.	The novel porous silicon Bragg reflector SERS-active structure significantly enhanced Raman signals, enabling sensitive detection of breast cancer biomarkers in tissue and serum samples. By combining photonic bandgap effects with plasmonic silver nanoparticles, the biosensor achieved strong signal amplification and reproducibility. The results demonstrated clear discrimination between cancerous and non-cancerous samples, confirming the potential of PSBR-SERS structures for clinical breast cancer diagnostics.	Chinese Optics Letters 1.8	127 China (2021)	[56]
Exosomal	Serum	AuNPs	Develop a multiplexed, quantitative biosensor platform that integrates SERS with vertical flow technology for breast cancer subtyping.	SERS-vertical-flow biosensor can multiplex and quantitatively profile exosomal proteins in patient serum samples (MUC1, HER2 and CEA), enabling accurate breast cancer subtyping. Gold nanoparticle probes functionalized with Raman reporters and antibodies provided strong, reproducible signals, while the vertical flow design improved assay speed and minimized background noise. The biosensor achieved picomolar sensitivity, successfully distinguishing breast cancer subtypes in clinical samples, highlighting its potential for precision diagnostics and personalized treatment planning.	ACS Nano 15	126 China (2023)	[62]
Circulating tumor cell	Blood	AuNPs functionalized with Raman reporters and antibodies	Produce a multiplexed SERS-based biosensor platform integrated with a microfluidic chip for in situ profiling of circulating tumor cells.	Multiplexed SERS nanovectors combined with multivariate analysis can accurately profile circulating tumor cell phenotypes in situ using a microfluidic chip. Gold nanoparticle-based nanovectors encoded with distinct Raman reporters enabled simultaneous detection of multiple biomarkers at the single-cell level. The system successfully discriminated against heterogeneous CTC populations, highlighting its potential for liquid biopsy applications and precision oncology. This multiplexing capability represents a significant advance in cancer diagnostics, offering a powerful tool for monitoring tumor heterogeneity and guiding treatment decisions.	Small 13.8	120 China (2018)	[64]
Cell surface proteins		Gold nanobipyramids (AuNBPs) bioconjugated with targeting ligands (antibodies)	Integrate diagnostic and therapeutic functions in a single bioconjugated gold nanobipyramid platform for cancer treatment.	Gold nanobipyramids bioconjugated with antibodies can serve as dual-function agents for breast cancer diagnostics and therapy. Their sharp-tip geometry provided strong SERS enhancement, enabling single-cell detection of cancer biomarkers, while NIR irradiation induced efficient photothermal ablation of targeted cells. The results confirmed that AuNBPs can simultaneously deliver ultrasensitive detection and selective therapy, highlighting their potential as multifunctional nanoplatforms in precision oncology.	ACS Biomaterials Science & Engineering 5.8	120 China (2017)	[63]
Telomerase	Cells	Gold nanowires (AuNWs) with engineered nanogap-rich structures	Validate a label-free nanogap-rich Au nanowire SERS biosensor for ultrasensitive detection of telomerase activity in cancer diagnosis.	Nanogap-rich gold nanowire SERS sensors can detect telomerase activity with attomolar sensitivity, enabling accurate diagnosis of gastric and breast cancer tissues. The engineered nanogaps provided strong plasmonic enhancement, allowing clear discrimination between cancerous and non-cancerous samples. Validation in both cell lines and patient tissues confirmed the sensor’s robustness and clinical potential, establishing telomerase activity as a reliable biomarker for cancer diagnostics using SERS technology.	Advanced Functional Materials 19	111 China (2017)	[10]
Exosomal surface proteins		Au@Ag nanoparticles/graphene oxide (Au@Ag NPs/GO) substrate with 4-nitrothiophenol (4-NTP)	Analyze the dual-aptamer radiometric SERS biosensor functionality for breast cancer exosome detection with enhanced sensitivity, specificity, and reproducibility for clinical diagnostics.	The SERS biosensor can achieve ultrasensitive and precise identification of breast cancer exosomes. By combining two aptamers targeting different exosomal proteins with Raman reporters, the system provided radiometric signals that improved accuracy and reduced variability. The biosensor achieved femtomolar sensitivity, successfully discriminated against breast cancer patients from healthy controls, and validated its performance in both cell line-derived and clinical serum exosomes. This dual-recognition strategy represents a significant advance in exosome-based diagnostics.	ACS Sensors 7.7	108 China (2023)	[55]
CA15-3, CA125 and CEA	Serum	AgNPs	Design a microfluidic-SERS biosensor capable of multiplexed detection of breast cancer biomarkers in clinical samples.	The SERS-integrated microfluidic chip can simultaneously and sensitively detect multiple breast cancer biomarkers in real serum samples. Silver nanoparticle probes functionalized with antibodies and Raman reporters enabled multiplexed profiling of CA15-3, CA125 and CEA with high sensitivity. The biosensor successfully distinguished breast cancer patients from healthy controls, highlighting its potential for non-invasive, accurate, and multiplexed cancer diagnostics.	Talanta 6.1	106 China (2018)	[61]
DNA, proteins and lipids	Serum	AgNPs	Compare the diagnostic performance of SERS and conventional Raman spectroscopy for breast cancer detection using serum samples.	SERS using silver nanoparticles provided significantly stronger and more reproducible spectral signals compared to conventional Raman spectroscopy for breast cancer detection. Analysis of patient serum samples revealed that SERS could sensitively distinguish cancer patients from healthy controls, whereas conventional Raman spectroscopy showed weaker discriminatory power. The results confirmed that SERS is a superior technique for serum-based cancer diagnostics, offering enhanced sensitivity and diagnostic accuracy.	Spectrochimica Acta Part A: Molecular and Biomolecular Spectroscopy 5.2	104 China (2020)	[66]
Proteins, metabolites, nucleic acids	Blood	AuNPs deposited on a super-hydrophobic substrate	Develop a high-throughput, AI-assisted SERS platform for non-invasive blood analysis and multi-disease screening, using super-hydrophobic substrates with deep learning algorithms.	The super-hydrophobic SERS platform, combined with deep learning algorithms, enables high-throughput and highly sensitive blood analysis for non-invasive multi-disease screening. Gold nanoparticle-coated substrates provided strong Raman enhancement, while the super-hydrophobic design ensured reproducible droplet positioning. Deep learning models successfully classified spectral fingerprints from patient serum samples, distinguishing breast cancer and other diseases from healthy controls. This approach highlights the potential of combining advanced nanostructured SERS substrates with AI for clinical diagnostics.	Advanced Functional Materials 19.0	105 China (2021)	[57]
Exosomes	Serum-derived exosomes	Gold nanostructured substrates	Establish a label-free, machine learning-assisted SERS platform for cancer diagnosis and therapeutic monitoring using exosomes.	Gold nanostructured substrates provided reproducible Raman fingerprints of exosomes, while machine learning models classified these spectra with high accuracy. The system achieved femtomolar sensitivity, successfully distinguishing cancer patients from healthy controls and tracking therapeutic responses over time. This approach highlights the potential of integrating AI with SERS for precision oncology.	Analytical Chemistry 8.0	100 China (2023)	[33]
EGFR, HER2, ER, and CD44	Tissue	AuNPs	Develop and validate a Raman-encoded molecular imaging platform using topically applied SERS nanoparticles for intraoperative breast cancer surgery. The authors aimed to improve surgical precision by enabling sensitive, multiplexed visualization of tumor margins, thereby reducing recurrence rates and enhancing patient outcomes.	The topically applied SERS nanoparticles enable multiplexed molecular imaging for intraoperative guidance of breast cancer lumpectomy. Gold nanoparticles encoded with Raman reporters and antibodies provided strong, distinct signals for multiple tumor biomarkers. This approach allowed surgeons to visualize tumor margins with high sensitivity, reducing the risk of incomplete tumor removal. The results highlight the potential of SERS-encoded nanoparticles as a powerful tool for real-time surgical guidance.	Cancer Research 13.2	98 United States (2017)	[60]

**Table 2:** Study design, validation, limitations, and general judgment of the 20 most cited documents between 2016 and 2025 retrieved from Scopus databases.

Design and Effective Sample	SERS Validation	Model/Clinical Validation	Main Limitations	General Judgment	Reference
Multicenter clinical cohort, plasma/exosomes: 210 controls, 543 patients; training *n* = 233, test *n* = 520	PDMS chips with AuNPs EF 4.28 × 10^5^ CV 6.0% at 1364 cm^−1^	MIL-CNN classifier AUC: 0.970 Sensitivity: 90.2% Specificity: 94.4% CI reported BC AUC: 0.876	Retrospective; case–control; single population; no external validation or calibration; non-uniform colloidal hotspots.	Strongest clinical evidence; leading translational methodological study.	[38]
Clinical bioanalytical study. Exosomal RNA extracts from BC serum vs. qRT-PCR; four BC cell lines	3D Au nanopillar substrate (“head-flocked”) fabricated by RIE. Hybridization with LNA probes for miR-21, miR-200c (Cy3). RSD: 2.98–4.65% LOD: 1 aM–100 nM Recovery: 97–102%	Calibration of log-concentration vs. Cy3 at 1150 cm^−1^ (R^2^ > 0.95). Recovery: 97–102% RSD: 3–6% No patient evaluation and no AI.	Fortified serum and cell lines; no clinical accuracy; requires heating above T_m_ to avoid false signals.	Excellent plasmonic microsensing methodology; strong analytical evidence, preclinical.	[59]
In vitro study in HeLa, MCF-7, and A549 cell lines. Small RNA extracts	Magnetic capture probes (Fe_3_O_4_@Ag NPs) conjugated with DNA; ratiometric amplification via DSN. LOD: 0.3 fM Single-base selectivity and recovery efficiency, with SERS readout at 1586 cm^−1^.	Direct analytical validation vs. commercial RT-qPCR kit (let-7 copy count). Relative error: 2.6–12.1%. No AI, no human evaluation.	Limited to purified RNA extracts; not validated in native serum/plasma. Insufficient documentation of reproducibility. Long incubation (60 min/55 °C).	Analytical evidence of good quality; DSN enzymatic amplification avoids false positives from nonspecific hybridization.	[17]
In vitro multiplexed study. Individual cultures and complex co-cultures; 5 breast cancer cell lines (MCF-7, SK-BR3, HCC-1395, CAMA1, MDA-MB-231)	Ratiometric SERS probes (RaR) based on Au NSs encapsulated in PMA polymer, loaded with 5 orthogonal thiol reporters. LOD: 7.8 × 10^8^ particles/mL.	DCLS unmixing algorithm (commercial software). 84% classification accuracy of individual cells. No clinical diagnostic model.	16% of cells not identified due to low nanoparticle uptake; no human participants.	Good biophysical rigor in vitro; ratiometric NSs suitable for spatial/temporal tumor monitoring. Preclinical technical evidence.	[54]
In vitro bioanalytical study. Synthetic BRCA1 dsDNA adsorbed to sensor electrode instead of cellular samples and DOX	Hybrid SERS–electrochemical biosensors (DPV). Electrode modified with rGO and Fe_2_Ni@Au core–shell magnetic NPs with immobilized DNA. LOD: 8 µg/mL for DOX.	Dual ratiometric analytical validation. DPV current measurement (LOD: 0.008 mg/mL, R^2^ = 0.994). No AI, no cancer detection.	No validation with clinical samples (serum or complex cells); not diagnostic.	Excellent prototype of bioanalytical instrumentation and hybrid electrode. Analytical evidence outside diagnostic corpus.	[65]
In vitro study with small clinical validation. 8265 exosome spectra from 4 cell lines for training; patient samples for testing	Dense monolayer of AuNSs, plasmonic peak at 772 nm (NIR). Incomplete reproducibility characterization.	ANN/CNN with 4 hidden layers Spectral partition: 80/20 Clinical accuracy: 79.4% Training accuracy: 95%	Severe spectral pseudo-replication (thousands of spectra from only 4 cultures); trained on cells, applied to patients; no external validation or calibration.	Excellent physicochemical concept; high risk of algorithmic bias and overfitting.	[19]
In vitro unicellular study. Mapping BC cells (SK-BR-3, MDA-MB-231)	Stable Ag-Au nanotags resistant to salt, pH, temperature; ratiometric reporters (RBITC, MGITC, DTDC); functionalized with CD44, EpCAM, ErbB2 antibodies.	Multiplexed in situ correlation validated against Western blot phenotyping. No AI, no human evaluation.	Limited to in vitro cell mapping.	Very good bioimaging methodology; antibody-functionalized hollow spheres reduce receptor heterogeneity. Adequate preclinical technical evidence.	[58]
Preliminary clinical study. Tear samples from 5 cancer patients and 5 controls; 10 spectra per participant	Honeycomb-pattern polystyrene chips coated with gold (Au/HCP-PS) to suppress coffee-ring effect. EF: 5.4 × 10^10^ LOD: 100 fM RSD: 6.5% and 3.5%	PCA-LDA with LOOCV; preprocessing with Savitzky–Golay, rubber band, vector normalization. Clear separation (86.7% cumulative spectral variance).	Only 10 participants; case–control design; possible dependence between spectra.	Excellent non-invasive sensing concept; suppression of coffee-ring effect and portable equipment favor applicability. High risk as pilot clinical study.	[26]
Preclinical in vivo model. Mice with 4T1 breast tumors and rabbits with knee osteoarthritis	Dual ratiometric colloidal probe is based on AuNRs loaded with aspirin. Bimodal SERS and PA system; selective detection of ROS.	Analytical and in vivo validation. Ratiometric PA/SERS kinetics plateau at 2 h post-injection (intratumoral/intraarticular). No AI, no human evaluation.	Not applicable to clinical breast cancer detection.	Technical preclinical evidence; outside clinical corpus.	[30]
Retrospective clinical study. 30 serum samples (early BC vs. 30 controls); 3 spectra averaged per person	Porous silicon Bragg reflector decorated with AgNPs. Signal ×3 vs. flat silicon. EF 10^11^ with R6G Limited batch reproducibility	PCA-LDA with LOOCV; 8 PCs explaining: Variance: 94.8% Sensitivity: 93.3% Specificity 96.7% Accuracy: 95%	Moderate monocentric cohort; retrospective two-gate case–control design; internal validation only; no calibration or CI.	Strong plasmonic substrate engineering; exploratory performance.	[56]
Preliminary clinical study. Serum from 26 participants: 5 controls, 21 patients across 4 subtypes (Luminal A, HER2+, Triple Negative, Luminal B)	Rapid nitrocellulose vertical flow device (iREX). Au NS probes coded with p-NTP and multiplexed aptamers for MUC1, HER2, CEA; differentiated test zones. LOD: 1.0–2.9 × 10^7^ particles/mL.	Direct ratiometric clinical validation comparing intensity at 1328 cm^−1^ vs. CD63 spot. Subtypes established by IHC. No AI.	Small cohort; only 5–6 subjects per group; no independent test, blinding, global accuracy, or CI.	Adequate point-of-care device design (vertical flow/NC formats); avoids false negatives; exploratory clinical feasibility.	[62]
Ex vivo unicellular study. 126 spectra/cells from 3 BC lines (SKBR3, MCF7, MDA-MB-231) and Jurkat control; artificially injected into donor blood	Microfluidic chip (12 µm capture barriers); 3 orthogonal Au@Ag aptamer nanovectors (SAVs); spectral normalization via rCLS.	Supervised PLS-DA classifier with CV: Sensitivity: 87.5–96.77% Specificity: 95.75–97.92% Blind test with spiked cells.	Limited to artificial samples (blood enriched with commercial cultures); no patient CTCs; unclear test size.	Outstanding methodological quality for SERS signal normalization; high model risk; preclinical evidence.	[64]
Preclinical in vitro and in vivo study. BC (MCF-7), hepatocellular carcinoma (HepG2), normal mammary epithelial (MCF-10A). Validated in nude mice with MCF-7 tumors	Colloidal probes based on Au NBPs. Encoded with 2-naphthalenethiol reporter; functionalized with PEG and folic acid. LOD: 5 cells/mL.	Preclinical and theranostic validation. No AI, no diagnostic accuracy.	Preclinical theranostic study; no human samples.	High-quality theranostic nanotechnology study.	[63]
Preclinical model, cellular and animal study; tissues from 16 mice bearing gastric and breast xenografts (SNU-484, MDA-MB-231)	Gold NW rich in nanogaps decorated with AuNPs. TS primer hybridization and methylene blue exchange. EF: 1.34 × 10^4^ RSD: 4.80% at 1620 cm^−1^ (excellent analytical stability) LOD: 0.2 cells/mL	No AI, no human samples.	No clinical validation; cellular and xenograft models.	Technically adequate preclinical evidence, with physical reproducibility of the plasmonic substrate.	[10]
Preliminary clinical study. Serum from 11 BC patients, 12 pancreatic cancer patients, and 8 controls	Plasmonic ratiometric biosensor (Au@Ag NPs/GO) for immunomagnetic exosome capture via anti-EpCAM aptamers. LOD: 1.5 × 10^2^ particles/mL RSD: 8.5% Signal retention at 30 days: 91% EF: 7.33 × 10^6^	Direct ratiometric validation via two-tailed t-test. Significant differences (*p* < 0.001) between patients and controls. No AI.	Small cohort (*n* = 31); EpCAM is general to epithelial tumors; case–control design; no formal accuracy or CI.	Outstanding plasmonic biosensor development; technically adequate, exploratory clinical evidence.	[55]
In vitro immunoanalytical study. Human commercial serum enriched with recombinant tumor analytes and 2 real samples	Immuno-SERS biosensor with continuous microfluidic injection channels. AgNPs (53 nm) modified with 4-MBA and antibodies against CA15-3, CA125 (LOD: 0.01 U/mL), CEA (LOD: 0.1 pg/mL); 21-day stability.	Quantitative analytical validation. Linear ratiometric calibration curves (R > 0.95) at 1333, 1080, 1378 cm^−1^. No AI.	Only 2 real samples; cannot calculate sensitivity, specificity, or generalization.	Excellent microfluidic immuno-SERS biosensor. Analytical validation only, not clinical.	[61]
Retrospective clinical study. 29 participants: 17 BC patients (stages II–IV), 12 controls; 15 spectra per participant	Spectra from mixing 50 µL native serum with 50 µL AgNPs on aluminum support (785 nm laser). Reproducibility shown qualitatively; no RSD or batch evaluation.	Multivariable PLS-DA classifier with 14 latent variables: AUROC: 0.94 Sensitivity: 90% Specificity: 98.4%	Retrospective case–control design; small sample size; pseudo-replication risk; no external validation or CI.	Demonstration of comparative accuracy; exploratory performance.	[66]
Clinical serum cohort: 695 participants (203 controls, 321 breast cancer, 77 leukemia M5, 94 HBV)	EF: 6.21 × 10^7^ LOD: ~10^−12^ M for R6G RSD: 2.3–3.7% Coffee-ring control Superhydrophobic platform.	5-layer DL model; training *n* = 626; diagnostic accuracy 98.6% in held-out test set.	Test set from same population; retrospective case–control design; no calibration or CI.	Relevant SERS-DL evidence; no external validation.	[57]
In vitro study with limited clinical validation. Exosomes from 3 cell lines (H8, HeLa, MCF-7); 5 BC patients, 5 cervical cancer patients, 5 controls	Trilaminar plasmonic substrate of AuNPs. EF: 2.4 × 10^4^ RSD: ~3.3% Stability: 30 days	PCA-LDA with 46 PCs; random 80/20 partition; clinical serum accuracy 93.3%, in vitro accuracy 91.1%.	Batch separation unclear; only 5 subjects per group; no external validation or CI.	Excellent physical engineering; high risk, preliminary clinical evidence.	[33]
Prospective translational clinical study. 57 fresh surgical tissue specimens from 29 lumpectomy patients; 2106 ROIs	REMI ratiometric imaging system with 4 Au@SiO_2_ nanocapsules (120 nm) targeting HER2, EGFR, ER, CD44; detection at 785 nm with ultrafast CCD.	DCLS algorithm; H&E reference: Sensitivity: 89.3% Specificity: 92.1% Per-patient: 92.9%	Exclusion of 74 mixed ROIs (tumor/benign) to avoid pixel-to-pixel co-registration issues; threshold developed/evaluated in same cohort; ROI correlation.	Strong technical rigor; automated identification of carcinomas in fresh tissue within <15 min; ex vivo clinical feasibility.	[60]

PDMS: Polydimethylsiloxane; MIL-CNN: Multiple Instance Learning; RIE: reactive ion etching; DSN: duplex-specific nuclease; DCLS: direct-classical-least-squares; NSs: nanostars; PA: photoacoustic; NBPs: nanobipyramids; NW: nanowires; DOX: doxorubicin; AUC: area under the curve.

### 3.2. Journal Distribution and Macro-Keyword Networks

**Figure 4:**
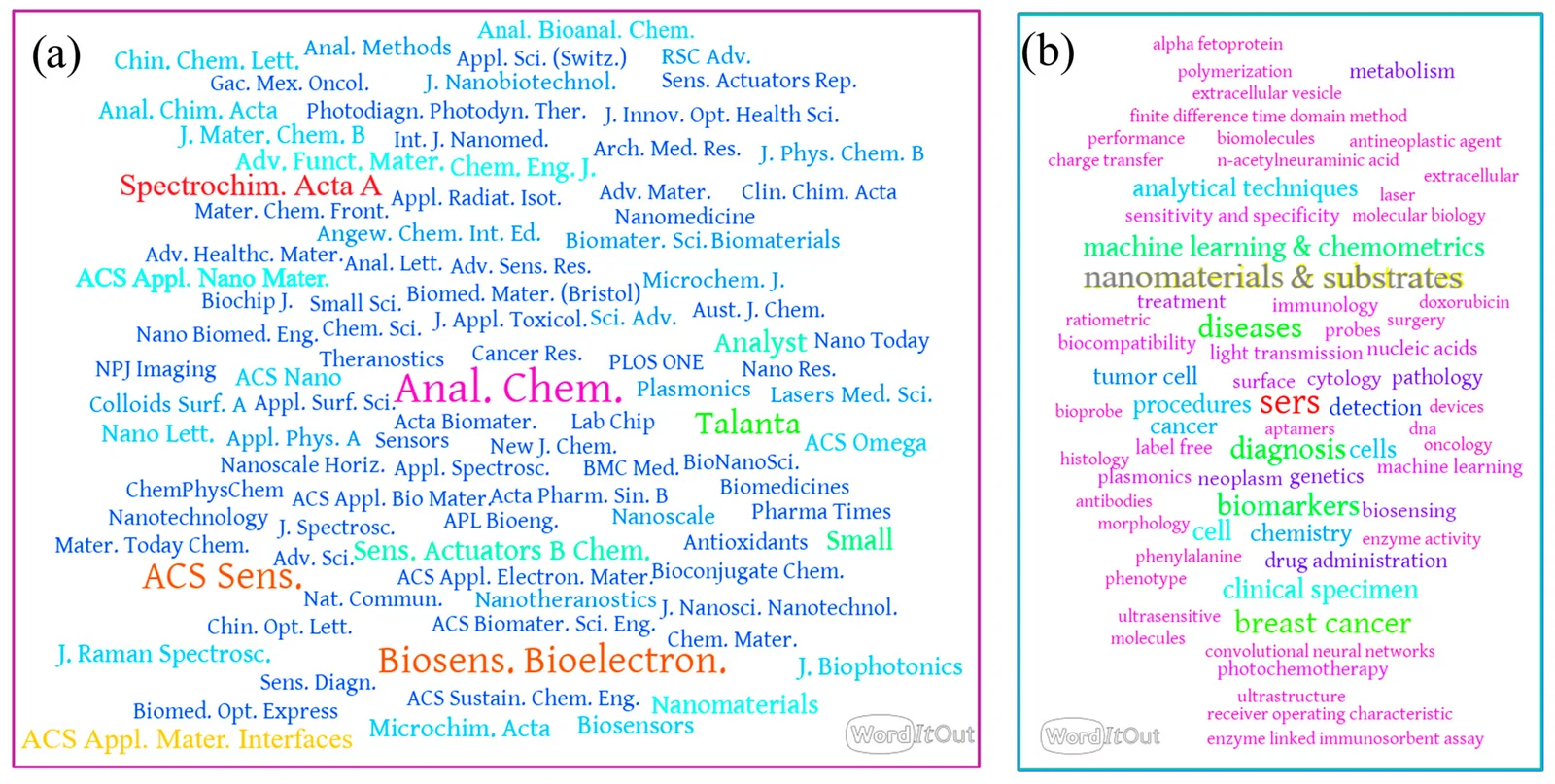
Word clouds generated from the Scopus dataset: (**a**) journal cloud including terms with frequency ≥1, similarly, scaled to reflect relative occurrence, and (**b**) keyword cloud including terms with frequency >3, scaled using the “minor” option for word size differences. Colors denote similarity in frequency, while font size reflects relative variation: larger letters correspond to higher frequencies, whereas smaller letters indicate lower values.

The visualization of publication journals in Figure 4a presents the preferred dissemination channels and underscores the interdisciplinary framework of SERS applications in breast cancer detection and diagnosis. According to the word cloud, although the research in this field is fundamentally based on physical chemistry, it operates at the intersection of several scientific domains. The prominence of specific recognized journals allows the current research landscape to be categorized into four main thematic pillars: (1) analytical and spectroscopic methodologies, (2) biosensing and engineered devices, (3) nanomaterials synthesis and substrate design, and (4) clinical translation and oncology.

The predominance of leading journals such as Analytical Chemistry, Spectrochimica Acta Part A: Molecular and Biomolecular Spectroscopy, Talanta, Analyst, and ACS Omega, among others, within the first theme confirms that fundamental analytical validation remains the basis of the field. Publications in these media predominantly focus on the rigorous characterization of spectral signatures, LOD, signal reproducibility, and the foundational physics of Raman scattering in complex biological matrices.

The second theme is characterized by highly cited journals, including ACS Sensors, Biosensors and Bioelectronics, Sensors and Actuators B: Chemical, Chemical Engineering Journal, and Biosensors, among others. They are journals that reflect the strategic shift toward transitioning benchtop SERS methodologies into viable, miniaturized, practical diagnostic devices. Consequently, this category emphasizes the integration of active SERS substrates with microfluidics, lab-on-a-chip technologies, and point-of-care biosensing architectures.

The third theme, focusing on nanomaterials and substrate design, highlights the intrinsic role of materials science in synthesizing platforms with optimized plasmonic properties. Notably, leading journals such as ACS Applied Materials & Interfaces, Small, ACS Applied Nano Materials, Advanced Functional Materials, and Nanomaterials, among others, underscore that substrate engineering represents a critical technological bottleneck for SERS applications. Achieving the ultrasensitive enhancement factor required to identify early-stage breast cancer biomarkers demands continuous innovation in plasmonic nanomaterials. Accordingly, research in these venues drives the synthesis of novel gold and silver nanostructures, exploring complex morphologies and surface functionalization to maximize the electromagnetic enhancement mechanism.

While initial scientific efforts focused on optimizing analytical performance, advancing biosensor design, and synthesizing novel plasmonic substrates to achieve ultrasensitive and specific detection thresholds, the field has increasingly intersected with clinical oncology. At this stage, biologically and medically oriented journals such as the Journal of Biophotonics, Biomaterials, Journal of Nanobiotechnology, Lasers and Medical Science, Nanotheranostics, Photodiagnosis and Photodynamic Therapy, Theranostics, among others, illustrate the trajectory of the field. These publications prioritize the clinical utility of technology, addressing in vivo studies, non-invasive clinical liquid biopsies (e.g., blood, serum, and saliva analysis), and the validation of real-world diagnostic sensitivity and specificity against traditional histopathology.

These multidisciplinary journals demonstrate a robust and cohesive research line. The field has successfully bridged the gap between fundamental nanomaterial synthesis and rigorous analytical validation, with a clear and accelerating trajectory toward clinical diagnostic applications in oncology.

The keyword cloud (Figure 4b) outlines the conceptual landscape of the dataset, illustrating the most prevalent terminology in the research. Principal terms, including “breast cancer”, “SERS”, “diagnosis”, and “detection”, present the visual center of the cloud. However, the prominent inclusion of terms like “clinical specimen”, “procedures”, and “sensitivity and specificity” remains a field that is moving towards actively attempting to validate SERS protocols on real human samples. Then, highly evidenced terms such as “nanomaterials & substrates”, “plasmonics”, “chemistry”, and “surface” are related to the physical and chemical foundation of the technique. Because the efficacy of SERS is fundamentally dependent on the electromagnetic enhancement factor provided by metallic nanostructures.

Also, as observed in the cloud, keyword distribution provides deep insight into the specific biological entities being probed, in terms such as “cells”, “tumor cell”, “biomarkers”, “antibodies”, “DNA”, “biomolecules”, “alpha fetoprotein”, and “extracellular vesicle”. This demonstrates that SERS is increasingly being utilized for complex molecular profiling and liquid biopsy applications to detect circulating tumor DNA or tumor-derived extracellular vesicles (exosomes) at ultra-low concentrations, positioning it as a powerful tool for early-stage, non-invasive oncological screening.

Some critical emerging terms visualized in the cloud are related to methodological data interpretation: “machine learning & chemometrics”, “analytical techniques”, and particularly terms like “convolutional neural networks”, which illustrate a paradigm in analytical techniques. Due to biological SERS spectra being inherently complex, highly dimensional, and often obscured by background noise or overlapping molecular signatures. The integration of advanced AI and chemometric algorithms has become essential to decode these complex spectral fingerprints, enabling the automated, rapid, and highly accurate classification of healthy versus malignant samples.

Although both word clouds present a congruent macro-level representation of the principal research themes, their analytical utility is mainly confined to frequency-based distributions. While providing a valuable visual synthesis of the current state of the art, word clouds are inherently limited by their inability to capture topological connectivity, directional proximity, or hidden relation structures among terms. To overcome these limitations, to decode the explicit mathematical linkages between keywords, and expose the latent intellectual architecture of the domain, a robust network-based Keyword Co-occurrence Analysis (via VOSviewer) and a geometric MCA are systematically deployed in the following sections.

### 3.3. Keyword Co-Occurrence Analysis

**Figure 5:**
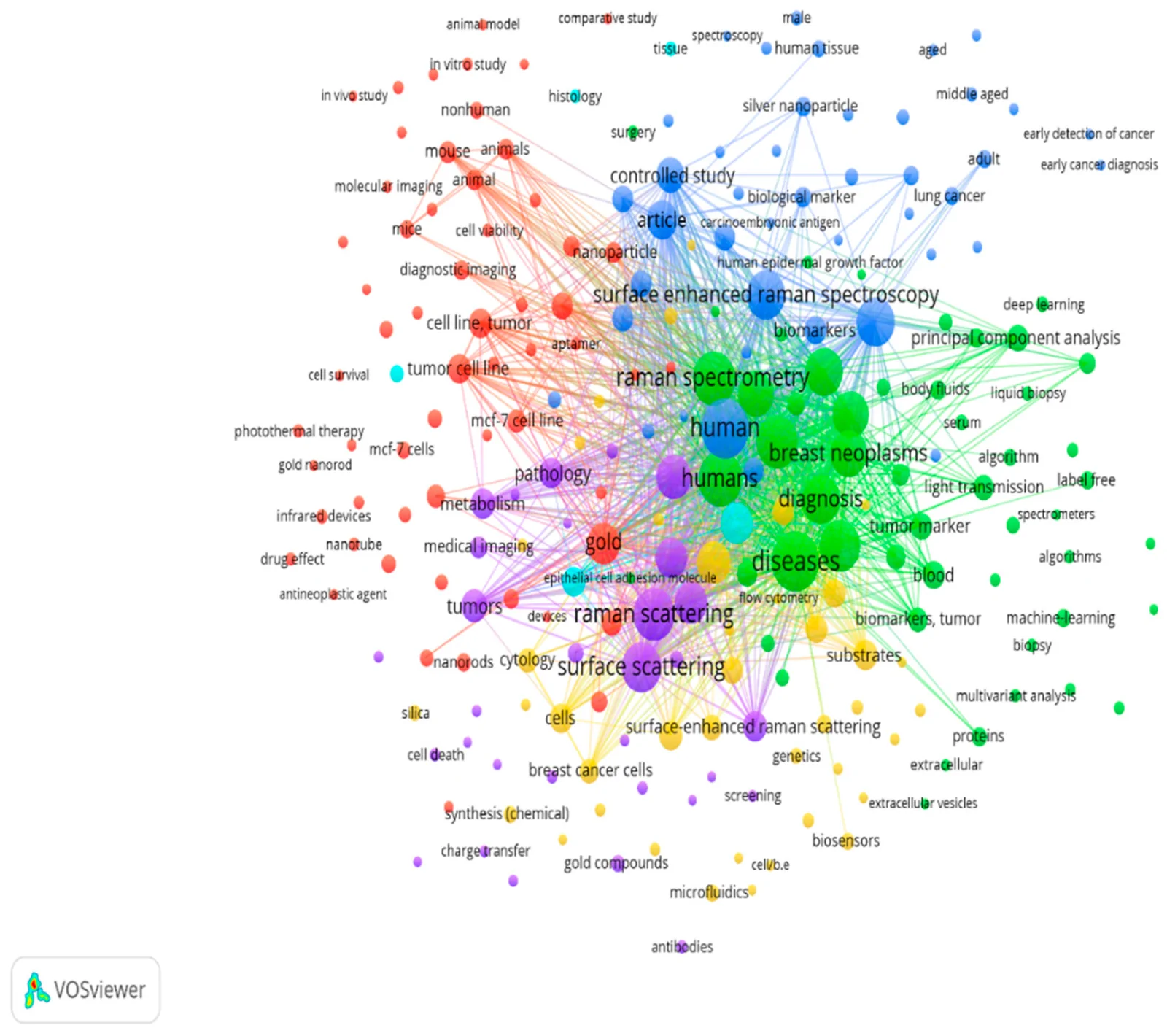
The keyword co-occurrence network was generated with VOSviewer, applying the association strength normalization method. The visualization highlights thematic clusters, each represented by a distinct color, and their interrelationships, with node size proportional to keyword frequency. Colors denote cluster membership, while font size reflects relative variation: larger letters correspond to higher frequencies, whereas smaller letters indicate lower values.

The VOSviewer keyword co-occurrence map reveals six well-defined clusters, differentiated by colors, as observed in Figure 5. In this visualization, each node represents a high-frequency keyword, with the node size proportional to its occurrence in the literature. Large, central keywords: “human/humans”, “breast neoplasms”, “diagnosis”, “Raman spectroscopy”, “surface-enhanced Raman spectroscopy”, “gold”, and “surface scattering” indicate that SERS methodology and clinical application dominate the literature. The links between nodes represent the co-occurrence of terms within the same publications, and the distance between them reflects their conceptual relatedness.

Clusters correspond to plasmonic substrate engineering (yellow cluster), methodology and human translation (blue and purple clusters), preclinical validation and biological models (red cluster), clinical diagnostics and computational integration (green cluster), and inter-cluster cellular biology and histopathological interfacing (cyan cluster).

The yellow cluster highlights the material science requisite for effective SERS analysis. The cluster is centered around “surface scattering”, “gold”, “chemistry”, “nanoparticles”, “substrates”, and “synthesis (chemical)”. It emphasizes that the continuous optimization of plasmonic nanomaterials remains a highly active research frontier. Connections from “gold” to “biosensors” and “microfluidics” demonstrate the ongoing engineering efforts to integrate optimized metallic nanostructures into automated, reproducible lab-on-a-chip devices suitable for clinical environments. The blue and purple clusters represent the central hub of the entire network; these clusters bridge the physical sciences with medical pathology. Including terms such as “surface-enhanced Raman spectroscopy”, “Raman spectrometry”, “human”, and “controlled study”, this region demonstrates the methodological core of the dataset. The proximity of “pathology” and “metabolism” suggests that researchers are actively utilizing SERS to establish metabolic fingerprints of malignant tissues, moving beyond simple detection toward a deeper understanding of tumor microenvironments and biochemical alterations.

Regarding the red cluster, about preclinical validation and biological models, it is dedicated to the experimental biological validation of SERS technologies, including terms such as “animal”, “mouse”, “in vitro study”, “in vivo study”, “tumor cell line”, and “MCF-7 cells”, underscoring intermediate steps before clinical translation. The strong connection between this cluster and the spectroscopic core indicates that a substantial proportion of the current literature is focused on evaluating the biocompatibility, targeting efficacy, and real-time molecular imaging capabilities of newly synthesized SERS nanoprobes in controlled in vitro and murine models.

In contrast, the green cluster reflects a step up in the translation process, referring to clinical diagnostics and computational integration, including terms from “breast neoplasms”, “diagnosis”, “diseases”, through “blood”, “serum”, “body fluids”, and “liquid biopsy” terms, hightlighting the robust scientific pivot to minimally invasive, blood-based diagnostic methodologies, towards, in the peripheral but significant presence of nodes, terms such as “machine learning”, “algorithms”, “principal component analysis”, “multivariant analysis”, and “deep learning”, demonstrating a critical methodological shift. The field is actively integrating artificial intelligence and advanced chemometrics to decode complex multivariate SERS spectra, thereby enhancing diagnostic specificity and mitigating human error in spectral interpretation.

Finally, a small specialized cluster in cyan is observed with terms such as “epithelial cell adhesion molecule”, “tissue”, and “histology”, anchoring as a critical interface between molecular targeting and structural pathology, and specific structural designations of “tumor cell line”. This grouping highlights the advanced biological targets and validation methodologies within the SERS domain.

In general, the high centrality of clinical and methodological keywords suggests a research trajectory moving from proof-of-concept spectroscopy toward clinically oriented biomarker discovery and diagnostic model development. The emergence of machine learning keywords indicates an increasing emphasis on robust, automated interpretation of complex spectral data, an essential step for clinical translation. The co-location of biosensor and therapy-related terms points to parallel efforts in device development and multimodal therapeutic strategies.

### 3.4. Bibliographic Structure via MCA

**Figure 6:**
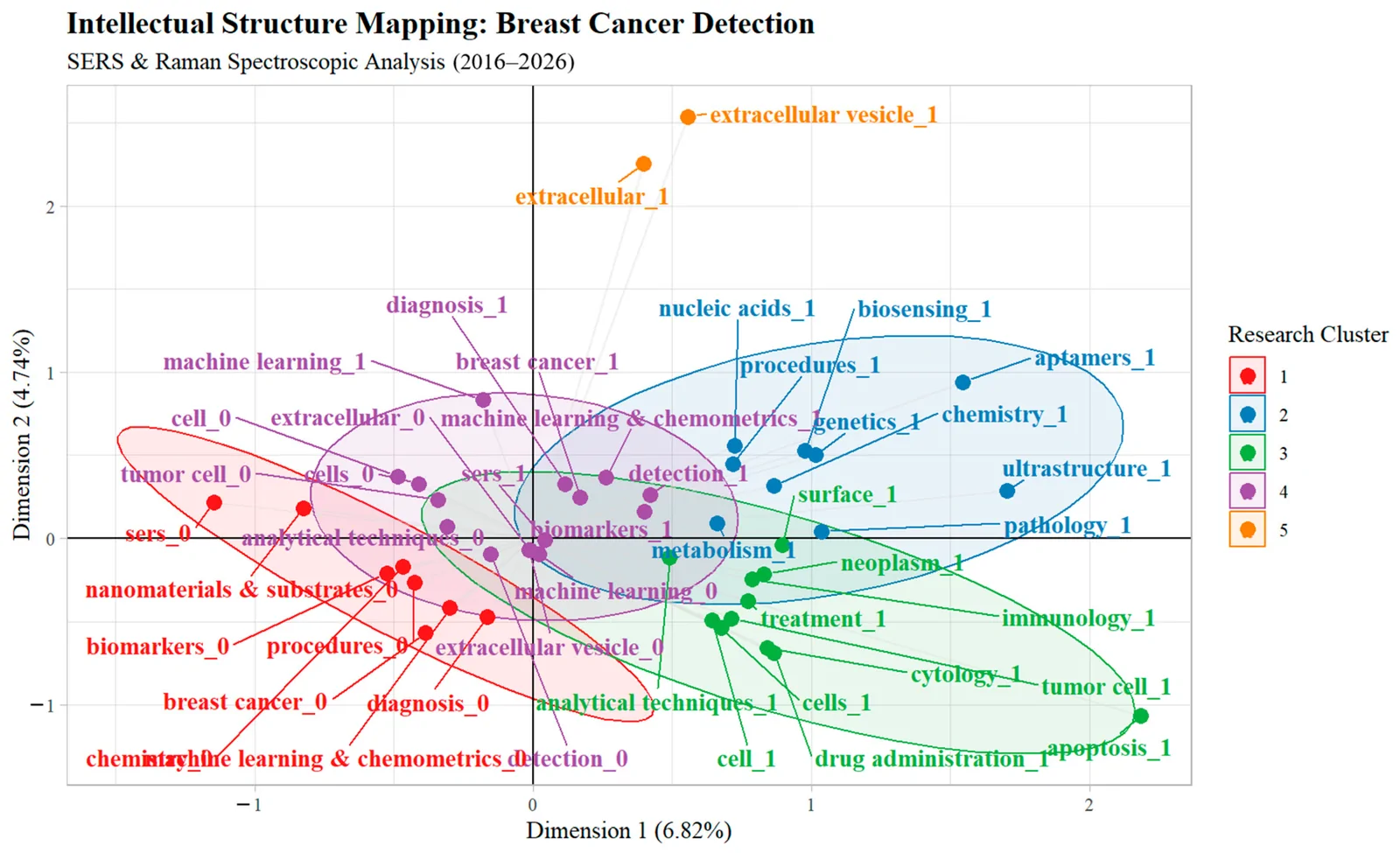
Multiple Component Analysis map of the thematic and intellectual structure of the research on Surface-Enhanced Raman Spectroscopy for breast cancer detection/diagnosis.

To further decode the underlying conceptual architecture and hidden structural relationships within the SERS breast cancer research, an MCA was conducted (Figure 6). Unlike the direct co-occurrence network depicted in Figure 5, which maps explicit linkages between terms, the MCA is a multivariate dimensionality-reduction technique that elucidates the complex dataset in a two-dimensional Euclidean space. In this representation, the proximity between keywords indicates a high degree of conceptual similarity and co-occurrence in the literature, while distance denotes thematic divergence. The map successfully partitions the intellectual structure into five distinct research clusters, distributed across Dimension 1 (6.82% of total variance) and Dimension 2 (4.74% of total variance). As observed in Figure 6, the arrangement along Dimension 1 reveals a distinct trajectory, transitioning from analytical chemistry on the left to advanced biological and clinical applications on the right.

In the MCA plot, five specialized thematic clusters can be identified that define the complete structure of the SERS breast cancer detection field.

The analytical and production domain (red cluster), located in the lower-left quadrant, includes terms such as “nanomaterials & substrates”, “chemistry”, “SERS”, “procedures”, and “diagnosis”. Its structural isolation on the negative side of Dimension 1 suggests that a substantial segment of the literature is strictly dedicated to the physical optimization of plasmonic platforms and analytical parameters, prior to complex biological integration.

The AI-assisted diagnostic interpretation (purple cluster) is situated in the center of Dimension 1 and bridges the origin of the map. This cluster represents the most highly integrated themes across the entire dataset. Integrated by terms such as “machine learning”, “chemometrics”, “biomarkers”, “diagnosis”, and “detection”, it illustrates the critical methodological bridge in modern SERS research. Furthermore, it highlights how researchers have combined the paradigm of basic physicochemical properties of substrates and the SERS method with advanced computational algorithms to decode complex biomarker signatures for accurate breast cancer diagnosis.

The molecular biosensing and genetic targeting (blue cluster) is placed in the upper-right quadrant. This group shifts the focus toward highly specific molecular biology, including terms such as “aptamers”, “nucleic acids”, “biosensing”, “genetics”, and “ultrastructure”, which outline a complex research area. Essentially, demonstrating the transition from generic cellular detection toward the design of highly engineered, targeted SERS nanoprobes capable of recognizing genetic profiling and specific biomolecular structures at the ultrastructural level and lower concentrations.

The liquid biopsy frontier (orange cluster), located prominently in the upper center of the map, this small but highly distinct cluster that centers exclusively around “extracellular vesicle”. The separation from the main body of clusters highlights it as an emerging, highly specialized, and cutting-edge niche. This signifies a major contemporary push toward liquid biopsies, utilizing SERS to isolate and molecularly profile tumor-derived exosomes from bodily fluid for early, non-invasive diagnostics.

Cellular pathology and pharmacological monitoring (green cluster) occupy the lower-right quadrant. This cluster is dedicated to both clinical and pre-clinical research on the biological mechanisms and therapeutic applications of SERS in breast cancer. It features terms such as “apoptosis”, “drug administration”, “metabolism”, “immunology”, “neoplasm”, “cytology”, and “treatment”. Through these words, it is inferred that SERS is evolving beyond static detection toward a dynamic monitoring tool capable of evaluating real-time cellular responses to antineoplastic drugs, tracking tumor metabolism, and investigating the immunological microenvironment of neoplastic tissues. The presence of both clinical and pre-clinical terms underscores the translational trajectory of SERS, bridging fundamental cellular studies with applied therapeutic monitoring.

The MCA plot reveals a research landscape composed of diverse, interconnected thematic areas, including the fundamental synthesis of nanomaterials and substrate development (Cluster 1), in-depth biological analysis (Clusters 2 and 3), computational spectral analysis (Cluster 4), and the study of extracellular vesicles (Cluster 5). These groups reflect the diversification and evolution of research topics within the field. However, the observed thematic structure should not be interpreted as evidence of translational maturity, clinical readiness, or future clinical implementation. Rather, it reflects areas that have garnered increasing attention from the scientific community and constitute active lines of research within this domain.

Once the macro-level bibliometric landscape was established and mapped, the topological and spatial configurations of the literature were analyzed through keyword co-occurrence networks and multiple correspondence analysis (Section 3.1, Section 3.2, Section 3.3 and Section 3.4); the focus of this review shifts from quantitative performance metrics to a qualitative evaluation of the dominant research fronts driving the discipline. While science mapping successfully illuminates where, when, and how frequently specific paradigms intersect, it inherently lacks underlying molecular mechanisms, chemical innovations, and clinical realities that dictate these trends.

To deconstruct the technological bottlenecks and breakthroughs that define this field, the subsequent sections (Section 3.5, Section 3.6, Section 3.7, Section 3.8 and Section 3.9) consolidate the transition from bibliometric analysis to a comprehensive, state-of-the-art scientific discussion. This analysis is organized based on the previous identified MCA spatial clusters, keeping an evolutionary trajectory of a SERS biosensor, beginning with the materials science of plasmonic substrate engineering (Section 3.5) and the chemistry of surface biofunctionalization (Section 3.6); moving through its analytical deployment in liquid-biopsy matrices (Section 3.7); examining the computational algorithms required for automated spectral decoding (Section 3.8); and, ultimately, exploring the preclinical and clinical translation (Section 3.9).

### 3.5. Surface-Enhanced Nanostructured Platforms for Breast Cancer Diagnosis

The bibliometric and structural landscape mapped by the MCA identifies the engineering, production and optimization of advanced plasmonic and hybrid nanostructured substrates as one of the main clusters. Some papers from the large principal list (199) were classified in this cluster and categorized into four structural classes based on their core nanomaterial structure, with the 10 most cited presented in Table 3.

Recent BC-focused studies extend this evolution toward electroporation-assisted intracellular probes, gold-modified microneedles, SERS-active structures embedded in three-dimensional tumor models, high-brightness NIR agents, and hierarchical chips that regulate molecular access to plasmonic hotspots in complex tissues [67,68,69,70,71,72]. These platforms are engineered to resolve the bottlenecks of SERS, signal irreproducibility and low chemical enhancement, while improving LOD down to a few cells, picomolar and femtomolar thresholds.

The landmark publications in this cluster signify a paradigm shift toward organized, reproducible, and multidimensional architectural designs. The principal thematic axis is the strategic interaction between the EM and CM to maximize the EF through hotspot formation. SERS has been dominated by the EM, which relies on the confinement of LSPR within “hotspots” of noble metals; however, CM has gained relevance through recent studies, improving electron donation, charge transfer, among other mechanisms.

**Figure 7:**
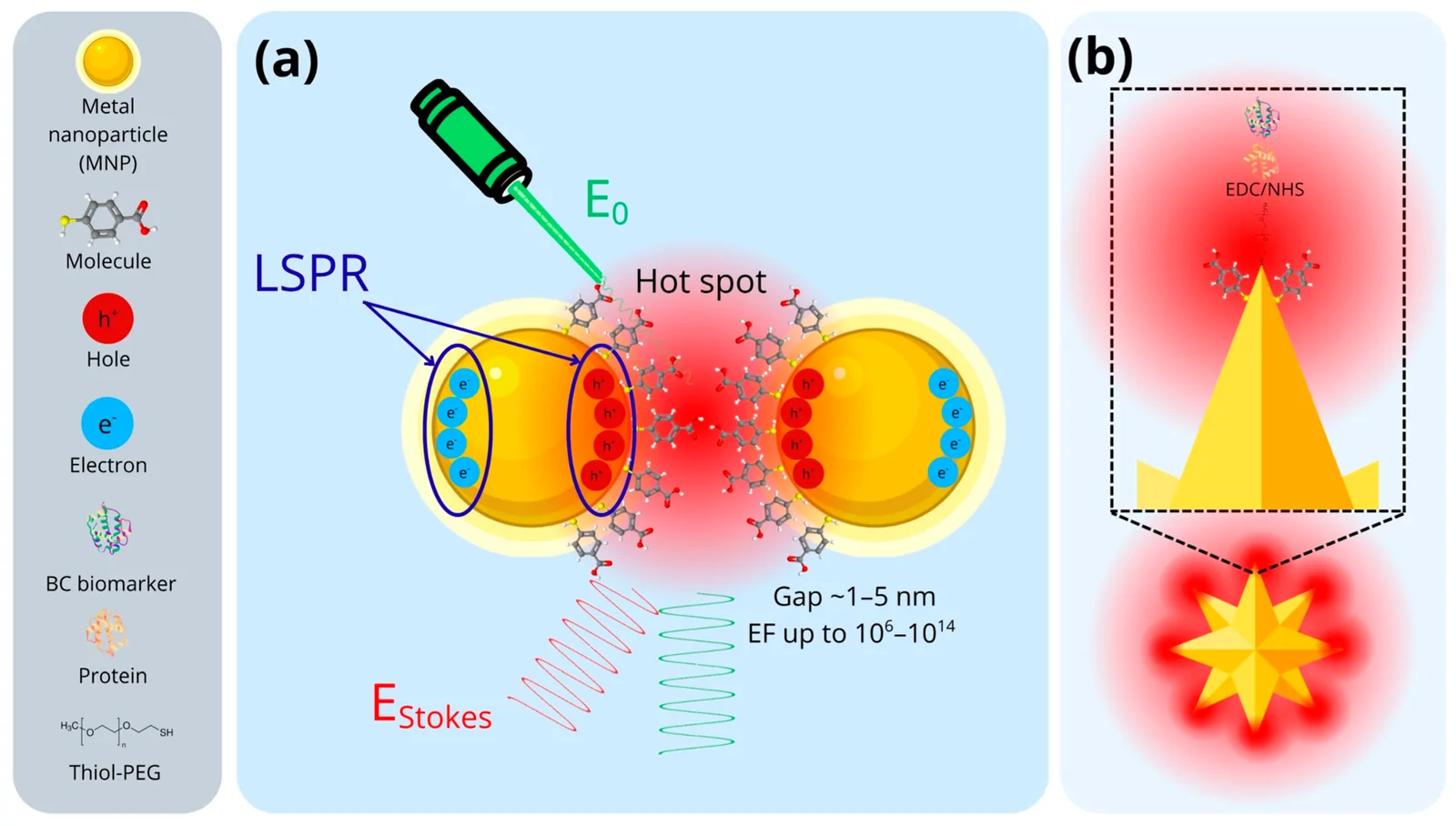
Schematic representation of hotspot formation and its relevance to SERS biosensing in breast cancer: (**a**) Incident excitation (E_0_, green) induces localized surface plasmon resonance (LSPR), polarizing the nanoparticle surface into electron-rich and hole-rich regions. Constructive near-field coupling within the nanoscale gap (~1–5 nm) confines and amplifies the electromagnetic field, producing enhancement factors (EF) up to 10^6^–10^14^. Molecules positioned within this region experience dramatically intensified Stokes-shifted scattering (E_Stokes_, red). (**b**) Tip-localized hotspot: Independent hotspot geometries arise at sharp nanoparticle features, where curvature concentrates the electromagnetic field via the “lightning rod effect”. A thiol PEG linker anchors covalently to the gold tip through Au-S bonding, while its distal carboxyl terminus is conjugated to capture proteins using EDC/NHS chemistry. These proteins selectively bind breast cancer biomarkers directly within the region of maximal field intensity. The dashed arrow illustrates how this mechanism is realized in branched nanostar architectures, where multiple sharp tips generate a high density of hotspots across a single particle, thereby enhancing multiplexed biomarker detection.

Figure 7 illustrates how nanostructure morphology governs the spatial distribution, magnitude, and biochemical accessibility of the electromagnetic hotspot underlying SERS-based BC biomarker detection. For understanding, two complementary geometric routes to field confinement are illustrated. In interparticle-gap architectures, the near fields of two adjacent plasmonic nanoparticles couple constructively across a nanometer-scale gap (typically 1–5 nm) upon LSPR excitation, producing a spatially confined hotspot with reported enhancement factors of 10^8^–10^14^, several orders of magnitude above the weak, uniformly distributed field at an isolated particle surface [73]. In sharp-tip architectures such as branched nanostars, comparable field concentration is achieved through curvature alone via the lightning-rod effect, without requiring interparticle coupling; each additional tip on a branched structure constitutes an independent hotspot, multiplying the effective hotspot density per particle [74]. Critically, the diagnostic value of either geometry depends on positioning the analyte precisely within this confined field. This is achieved through a defined bioconjugation route: a thiol-terminated PEG linker anchors covalently to the metal surface via an Au-S bond, while its distal carboxyl terminus is activated through EDC/NHS carbodiimide chemistry to form a covalent amide bond with a capture protein (antibody, aptamer, or engineered binding protein, depending on the platform) [75,76]. This protein selectively captures the target breast cancer biomarker directly at the site of maximal field intensity, ensuring that the vibrational fingerprint of the captured biomarker, rather than of background biomolecules, undergoes the greatest signal amplification [77]. This geometry–function relationship rationalizes the diversity of nanostructure architectures reviewed below: platforms are engineered not simply to maximize the electromagnetic field in the abstract, but to co-localize that field with a biochemically accessible capture site, an interdependence made explicit through the bio-functionalization strategies discussed in Section 3.6.

Core–shell configurations constitute the most extensively explored class. Noble metal core–shells (Au@Ag, Ag@Au, hollow Au/Ag cages) remain prominent because they combine tunable plasmonic resonances with engineered internal gaps that trap Raman reporters or analytes. Some examples include the systems developed by Zhang et al. (2017), who used Ag-coated nanorods with pyridine-Ag^+^ coordination complexes to confine reporters and detect as few as 20 cells in blood-mimicking fluid [78] and Choi et al. (2020), who demonstrated Ag-encapsulated Au hollow nanospheres for multiplexed biomarker quantification down to 10^−7^ M [58]. Galvanic replacement derived from Ag nanocubes (NCs) into Au nanocages [79] and a three-dimensional Au@Ag nanosphere substrate [80] further illustrate how internal voids and ordered packing increase hotspot density and uniformity, improving both EF (10^10^), reproducibility, and sensitivity (picomolar (pM) concentrations).

Complementing these electromagnetic strategies, there is a clear shift toward semiconductor-assisted and free-noble-metal pathways, driven largely by the need to reduce the material costs of pure metals and to exploit charge-transfer mechanisms that augment CM. Lin et al. (2020) [81] exemplified this trend with a crystal-amorphous TiO_2_ core–shell nanoparticle in which an amorphous layer rich in oxygen vacancies aligns the highest occupied molecular orbital (HOMO)–lowest unoccupied molecular orbital (LUMO) levels of analytes, enabling highly efficient interfacial charge transfer and yielding an EF of 4.3 × 10^5^ [81]. Similarly, hybrid core–shell architectures such as Cu_2_O@Ag [82] and SiO_2_@Au [83] combine semiconductor or dielectric cores with plasmonic shells to deliver both strong electromagnetic hotspots and enhanced chemical coupling, reporting an EF of 9.04 × 10^9^ for Cu_2_O@Ag (detecting 4-nitrophenthiol) and an EF of 1.37 × 10^6^ for SiO_2_@Au (measured with Rhodamine B). Beyond core–shell proposals, more complex nanoassemblies, such as tentacle-versus-satellite structures described by Dey et al. (2020), have been engineered to optimize broadband near-infrared plasmonic coupling, enabling simultaneous SERS diagnostics and photothermal therapy [84]. Collectively, these semiconductor-integrated and hybrid designs illustrate a pragmatic route to cost-effective, chemically selective, and multifunctional SERS substrates suitable for translational biosensing.

Heterostructures, semiconductor structures composed of two or more semiconductor materials with different bandgaps, offer another route to synergistic enhancement. Mixed-dimensional systems such as graphene oxide (GO) coupled to Au nanostars (AuNs) [85] demonstrate plasmon–exciton coupling that amplifies both EM (10^7^) and CM (10^2^) effects, enabling exosome detection at 10^2^ particles/mL from triple-negative breast cancer (TNBC) and HER2(+). Hybrid nanoporous GaN/Au/Ag substrates [86] and the Ag_2_O-Ag-Psi system [87] show how band engineering and defect chemistry can be tuned to improve adsorption, charge transfer and analyte resonance, producing LODs in the 10^−13^–10^−11^ M range for model probes (rhodamine, R6G) and miRNA targets. Notably, noble-metal-free ternary heterostructures such as Fe_3_O_4_@GO@TiO_2_ [88] and InGaN [89] have achieved single-cell sensitivity for PD-L1 expression and circulating tumor DNAs, respectively, underscoring the potential of heterostructures to combine biocompatibility, multifunctionality and cost-effectiveness.

The third structural class, two- and three-dimensional templates and flexible matrices, addresses practical challenges in biosensing, sample handling, throughput, mechanical compliance and biofouling. By moving beyond planar substrates, 3D porous networks, nanocage arrays and flexible nanofiber mats increase analyte capture, concentrate targets within confined volumes and provide mechanically compliant interfaces for cells and vesicles. These structures, therefore, improve sampling efficiency and spectral reproducibility in complex biological fluids.

Representative examples illustrate the range of design strategies and analytical performance. AgNP-based 3D platforms have been applied to exosome profiling [8]. Nitrogen-doped amorphous carbon nanocages combine porosity with electronic modification of the carbon matrix to enhance charge transfer and analyte trapping, enabling label-free live-cell fingerprinting with a reported EF of 5.52 × 10^6^ [90]. TiO_2_-based 3D templates exploit oxygen vacancies and semiconductor–analyte interactions to detect epidermal growth factor receptor at limits comparable to noble-metal substrates without additional labels [7]. Hybrid cellulose platforms incorporating Ag@Au raspberry-like NPs have been developed for urine-based monitoring of BC progression [91], reporting EF values near 3.98 × 10^8^. Conversely, Au-nanourchin films engineered to minimize serum biofouling while maximizing tip-enhanced hotspots demonstrate how nanoarchitectonic control of film thickness and immobilization kinetics can enable label-free immunosensing directly in raw clinical samples [92].

Flexible substrates extend functionality to dynamic and in vivo mimetic measurements. Electrospun nanofiber mats decorated with branched Au nanostars permit spatial mapping of intra- and extracellular pH gradients, an important functional readout relevant to the tumor microenvironment [93]. Miniaturized templates such as PDMS mini-pillars with deposited AuNPs improve droplet homogeneity and repeatability for liquid-phase assays; Das et al. (2019) and Song et al. (2020) reported an LOD of 1 × 10^−12^ M for miRNA-1246 using this approach [94,95]. Self-assembled Langmuir–Blodgett films of stearic acid and gold nanorods provide another route to high-throughput cell line discrimination with demonstrated cytotoxicity and biocompatibility, using Principal Component Analysis (PCA) and spectral classification [94]. Simpler planar implementations, gold nanospheres on ITO [96], laser-ablated AgNP substrates [20] or Au/SiO_2_ [97] remain valuable for subtype discrimination and femtomolar sensitivity in reporter-based assays.

A critical design consideration is the preservation of analyte bioactivity during capture and measurement. Zhu et al. (2023) proposed SiO_2_ nanopore arrays with superwettable, omniphobic, lubricated surfaces that trap droplets and generate stable liquid hotspots [43]. The authors reported femtomolar sensitivity for R6G with reproducible signals maintained over 30 days. Carbon-based probes (graphite-derived or amorphous) offer degradability, fluorescence, and multifunctionality (drug delivery and imaging) for intracellular monitoring [98]. Finally, SERS analysis of histopathological tissue sections using AgNPs remains the clinical gold standard for biochemical mapping, identifying amino acids, proteins and nucleic acids with spatial resolution that complements molecular assays [99].

Together, these studies show that 3D templates and flexible matrices are not merely structural variants of planar SERS substrates but constitute a distinct design paradigm; they integrate physical trapping, directional hotspot orientation, and surface chemistry control to improve sensitivity, selectivity, and robustness in real biological matrices.

Finally, microfluidic platforms represent the translational frontier for point-of-care SERS diagnostics. Microfluidic systems enable controlled sample delivery, pre-concentration, and on-chip mixing with SERS substrates, reducing sample volume and improving reproducibility. Wang et al. (2024) demonstrated a femtosecond laser-fabricated capillary-actuated chip for H_2_O_2_ detection in MCF-7 intracellular medium with an LOD of 0.5 µM [100]. Other authors presented a platform to detect exosome secretion at the single-cell level, using three Raman reporters and three antibodies immobilized on the surface with AgNPs as probes, combined with machine learning [101]. Then, coupling microfluidics with engineered substrates, as Zhu et al. (2023) [43] and Park et al. (2024) [20] demonstrated, improves assay throughput and robustness while preserving high sensitivity.

Across these structural classes, several cross-cutting themes emerge. First, hotspot engineering, through controlled nanogap formation, 3D packing, or tip-enhanced geometries, remains the primary determinant of EF and LOD. Second, chemical enhancement via charge transfer is increasingly exploited to complement EM effects, particularly in semiconductor–metal hybrids where defect states and band alignment facilitate analyte-substrate electronic coupling. Third, reproducibility and stability are now central design criteria: internal standards (e.g., built-in reporters or stable oxide shells), robust fabrication methods (laser ablation, galvanic replacement, SILAR), and anti-fouling surface chemistries are commonly reported to reduce signal variability. Fourth, application-driven design, tailoring substrates for exosomes, circulating tumor cells, miRNA, or tissue imaging, has produced specialized platforms that trade off multiplexing, invasiveness and throughput according to clinical need.

In summary, as highlighted in Table 3, the evolution of BC detection using SERS should not be interpreted as a mere succession of nanomaterials. Rather, these investigations collectively suggest that nanoarchitecture governs the analytical process—from the localization of the electromagnetic field and charge transfer to probe organization, biomolecular recognition, signal stability, and device integration. However, analytical sophistication does not necessarily correspond to equivalent clinical maturity. The highest enhancement factors and lowest detection limits were generally achieved using model molecules such as R6G, 4NTP, or Crystal Violet, rather than breast cancer biomarkers measured in representative patient populations. Consequently, reproducibility was predominantly assessed across different positions within the same substrate, while only limited evaluations were conducted across batches, instruments, or laboratories. None of the studies provided clearly independent clinical validation. These findings indicate that the primary challenge is no longer simply maximizing SERS amplification, but rather designing nanoarchitectures whose molecular recognition properties, signal transduction, and reproducibility remain stable during large-scale fabrication and evaluation in representative clinical cohorts.

**Table 3:** Bibliometric metadata, plasmonic configurations, and analytical performance of the top 10 most-cited articles in Scopus within the “Surface-Enhanced Nanostructured Platforms for Breast Cancer Diagnosis” cluster from an MCA.

SERS System and Functionalization	Raman Reporters and/or Dye Probes and Biomarker	Experimental System and Effective Sample	Analytical Performance and Technical Reproducibility	Main Findings (Innovation)	Validation Level and Methodological Limitations	TC Journal Year Reference
**Type of nanostructure: core–shell**						
Au@Ag core–shell hollow nanosphere arrays. Multiplexed cocktail of monoclonal antibodies covalently immobilized onto plasmonic nanotags via carbodiimide chemistry (EDC/NHS).	RBTIC, MGTIC and DTDC, and antibodies (ErbB2, EpCAM, CD44)	Cell cultures of MCF-7, SK-BR3 and MDA-MB-231 lines in vitro. Multiplex imaging in living cells. Continuous pixel-by-pixel spatial mapping at 1.2 µm scan intervals on single cells fixed on coverslips with PLL. Number of independent cultures/cells: NR	Analytical performance - Spatial quantification. - Comparison of intensification according to Ag shell and reporter concentration. - LOD: 10^−7^ M and EF: N.R. - PEG avoided aggregation and relevant variations. - RSD and batch-to-batch reproducibility: NR - Classification of phenotypic profiles: Sk-Br3 expresses EpCAM(+)/ErbB2(+)/CD44(−) and MDA-MB-231 expresses CD44(+)/EpCAM(+)/ErbB2(−). Signals obtained from Raman: 1646 cm^−1^ (RBITC), 1615 cm^−1^ (MGITC), and 1133 cm^−1^ (DTDC). Reproducibility - Excellent colloidal stability and spectral consistency. - The PEG coating prevents aggregation of the nanospheres and Raman signal variations under extreme saline conditions (0–100 mM NaCl), pH variations, and elevated temperatures.	- Structure that combines the excellent intensification of silver in the gap and the great chemical stability of hollow gold without causing uncontrolled aggregation. PEG prevents the typical colloidal toxicity of silver in living systems. - Magnetic isolation and a tunable SERS biosensor capable of quantifying cellular phenotypic heterogeneity in a single run, minimizing false negatives from low single-marker expression. - Limitation: Exposed nanostructures (without PEG) show time-dependent cellular toxicity.	- Preclinical cellular/multiplex imaging. - No human samples or clinical classification - Effective experimental size: NR - The molecular profiles of the three biomarkers mapped by SERS are in close agreement with Western blotting data from the cell lines. *-* In vitro cell viability confirmed by WST-1 colorimetric assay.	142 *Biosensors and* *Bioelectronics* 2020 [58]
Core–shell plasmonic nanorods (Au-AgAu) 4-mp on the surface of the Au NR core grows a peripheral Ag shell by forming a pyridine-Ag+ Nanogap internal adjustable (≈2.5–3.3 nm) and free of conventional organic linkers.	4-mp and R6G Detection of CTCs overexpressing MUC-1. Controlled release of Dox induced by NIR light.	MCF-7 added to a fluid simulating HeLa and HEK blood as selectivity controls. No patients	Analytical performance - LOD: 20 MCF-7 cells and EF: NR. - Linear curve in the range of 20 to 12,000 cells based on reporter signal intensity at peak 1099 cm^−1^. - Temporal stability of the signal qualitatively demonstrated; composition/nanogap described as reproducible. - RSD:NR - MCF-7 cells incubated in vitro and evaluated in a fluid simulating blood with white blood cells and control cell lines (HeLa, HEK). - 150 nanoparticles analyzed individually using ImageJ software to characterize sizes and gaps. Reproducibility - Stable and quantitative electromagnetic field and SERS signal thanks to the reporters encapsulated in the gap that serve as an internal standard. The gap is adjusted by the concentration of silver and the precursor ligand. Limitation: High doses of 4-mp induce aggregation, reducing storage stability.	- Isolating the Raman reporter inside the sub-nanometer gap prevents bio-polluting or signal attenuation from complex whole blood matrix components (proteins, cells), enabling ultra-precise CTC quantification. - Free synthesis of organic linkers via coordination interactions and galvanic replacement driven by the Kirkendall effect. - It combines ultrasensitive diagnostics and controlled drug release in a single nanocarrier. - Limitation: Risk of aggregation and colloid instability for long-term storage at elevated 4-mp concentrations.	- Preclinical cell testing in simulated matrix. - Specificity tested using non-functionalized PNRs, pure AuNRs and control cells (HeLa, HEK) in white blood cell background. Feasibility by CCK-8 assay. - No CTCs from patients: experimental unit and number of batches NR.	71 *ACS Applied Materials & Interfaces* 2017 [78]
Titanium dioxide nanoparticles with a crystal-amorphous core–shell architecture (c-TiO_2_@a-TiO_2_) Direct surface functionalization with 4-MBA reporter molecules and cell-targeting ligands. Modified with AR, PD layer, and anti-P-glycoprotein antibody. SERS cellular imaging and PTT of drug-resistant cancer cells.	R6G, 4MBA, 4NBT, and CV Alizarin Red as Raman reporter; P-glycoprotein in MCF-7/ADR	- MCF-7 and MCF-7/ADR spectra obtained from 10 cells of each type. - Number of independent crops: NR - Multidrug-resistant MCF-7/ADR breast cancer cells in in vitro culture. - SERS images and spectra mapped on 10 cells of each type (MCF-7/ADR vs. normal MCF-7).	Analytical performance - LOD: 10^−6^ M for AR/ANBT and 10^−7^ M for R6G/CV; EF: 4.3 × 10^5^ (4NBT) - Raman signals ~4 times stronger than in its white W-TiO_2_ counterpart. - Consistency, stability, and reproducibility are referred to in the supplement. Reproducibility The crystal-amorphous heterojunction and the bending band provide excellent light absorption, density of states and probe adsorption stability. SERS signals show excellent uniformity, stability, and repeatability.	- Discovered that the outer amorphous layer induces dense oxygen vacancies that align HOMO-LUMO levels, maximizing the charge transfer mechanism for high-sensitivity noble-metal-free bioimaging, and that efficiently promote semiconductor-molecule photoinduced charge transfer. - Prevents coagulation and nonspecificity of plasmonic metals. - Limitation: EF (~10^5^) is lower than that of traditional plasmonic silver or gold substrates.	- Preclinical in vitro cellular and theranostic. - Only two cell lines and few cells; no human samples. - Visual evaluation; variation between crops/lots and formal accuracy: NR. - Efficiency of PTT proven using 808 nm laser (1.5 W/cm^2^) achieving cell death of MCF-7/ADR in 5 min. - Cell viability monitored with propidium iodide and calcein-AM.	82 *ACS Applied Materials & Interfaces* 2020 [81]
Cuprous oxide core and silver shell nanoparticles (Cu_2_O@Ag) were synthesized at room temperature on a Cu_2_O octahedral template. Label-free. Combination of photo-induced charge transfer and plasmonic resonance. Ultrasensitive detection and label-free classification of breast cancer cell subtypes.	4-NTP, MB Breast cancer phenotypes	- Four breast cancer lines (SK-BR-3, BT-474, MCF-7 and MDA-MB-231) - Fifty spectra per line for LDA classification model assisted by machine learning - Independent crops/lots: NR.	Analytical performance - LOD: 10^−15^ M for 4NTP, EF: 9.04 × 10^9^ (octahedra) and 3.98 × 10^9^ (hexahedra) - LDA classification: 93.3% with a 70/30 split and k-fold cross-validation. - RSD: 9.81% and 8.84% in 10 points for bands of 1080 and 1572 cm^−1^. - Subtyping accuracy of 93.3% using LDA model with k-fold cross-validation. Reproducibility Excellent signal stability and repeatability with an RSD of 8.84% (at peak 1572 cm^−1^) and 9.81% (at peak 1080 cm^−1^) for octahedral morphology	- Integration of Cu_2_O and silver. - Synergism of photoinduced charge transfer (PICT with 21.31% contribution) and surface plasmon (SPR) of Ag. - FDTD simulations confirm massive hotspots at Cu_2_O@Ag junctions. - Leveraged plasmon-semiconductor coupling generates an ultrahigh synergistic electromagnetic/chemical enhancement. When paired with machine learning, it accurately classifies complex subtypes.	- Cellular preclinical with multivariate classification. - Effective sample size depends on a few lines/cultures. - 200 independent spectra: no external evaluation or human samples. - 10-fold cross-validated classifier algorithm (88.10% average accuracy) and quantitative robustness assessment using ROC curves (excellent AUCs of 0.96 to 0.99 for classifying all 4 lines).	57 *ACS Applied Materials & Interfaces* 2017 [82]
**Type of nanostructure: heterostructures**						
Fe_3_O_4_@GO@TiO_2_ plasmon-free magnetic ternary heterostructure. Immobilization of anti-PD-L1 antibodies combined with thiolated reporter molecules. Recyclable magnetic platform based on charge transfer. In situ quantification and expression mapping of the immune receptor PD-L1 in tumor cells at the single-cell level.	Salicylic acid, oxalic acid, CC pyrocatechol, dopamine, PTCDA, CV, and CuPc; PD-L1	- Triple-negative breast cancer cells (HCC38, MDA-MB-231) and MCF-7 control cells.	Analytical performance - LOD: 3 cells HCC38 or 10^−7^ M and EF: 8.08 × 10^6^ for CuPc. - Calculated average PD-L1 molecules: 1729 (HCC38) and ~2973 (MDA-MB-231) per cell - PD-L1 quantification and comparison with ELISA. - Ten points used for EF. - Six replicate spectra were recorded per cell calibration point (*n* = 6) and three parallel experiments described. - No patients. Reproducibility Highly stable signals. CuPc retained ~97% of its Raman signal under prolonged laser exposure in free air. Excellent recyclability due to photocatalytic self-cleaning of TiO_2_ in visible light.	- Developed an immune checkpoint screening platform that quantitatively maps the spatial distribution of PD-L1 at a single-cell level, offering a highly accurate predictive tool for immunotherapy selection. - Multifunctional integration: magnetism for rapid separation/capture (Fe_3_O_4_), selectivity by chemical interactions of GO and molecular enrichment by the porous TiO_2_ shell. - PICT synergism between GO and TiO_2_ verified by DFT and TDR calculations. Limitation: Multilayer chemical synthesis process (Fe_3_O_4_, GO, TiO_2_ shell) very laborious and complex.	- Mapping the distribution of PD-L1 induced by IFN-γ stimulation. - Preclinical cellular/quantitative assay. The results correspond to selected cell lines; the LOD of three cells does not imply clinical sensitivity; no external validation. - Concordance validation against PD-L1 quantitative ELISA and Western blot assays. - Cell viability preserved at ~98% in cytotoxicity assays.	78 *Science Advances* 2018 [88]
Hybrid large area porous GaN nanostructures with Au/Ag layer (5 nm Au/50 nm Ag) and thiolated DNA capture probes linked via Au-S bonds.	Breast cancer microRNA detection: miR-K12-5-5p R6G, FAM	SERS microarrays incubated with synthetic microRNA in TE buffer and in real human serum from healthy patients. Signal uniformity evaluated over a continuous spatial mapping of 2500 points	Analytical performance *-* LOD: 1.13 × 10^−13^ M for R6G, 8.84 × 10^−10^ M for miRNAand EF: 2.5 × 10^8^ - Discrimination against non-complementary miRNA. - RSD ≈20% at 2500 points; signal decreased 22.7% at 30 days and 52% at 60 days in air. Reproducibility Signal distribution mapped with an RSD of ~20% over 2500 points in a 50 × 50 µm area. Stability up to 60 days in air (intensity drop of 22.7% at 30 days and 52% at 60 days).	- The porous GaN acts as a rigid, 3D-ordered, and thermally stable template sieve obtained by simple electrochemical etching and metallization that prevents metallic nanoparticle aggregation. - This layout yields a highly homogeneous hotspot density across wide areas, solving the classic signal reproducibility bottleneck in nucleic acid SERS detection. - Au partially protects Ag from oxidation. Limitation: Degradation of the plasmonic signal with storage time due to the susceptibility of Ag to oxidation in air	- Relatively high RSD and relevant temporal degradation; no clinical samples. - The LOD of the dye does not represent the LOD of the biomarker; reproducibility between batches NR. - Specificity against non-complementary mismatch microRNA (miR-4732) and negative controls in buffer and real human serum. - FDTD modeling interfacial electromagnetic distribution	52 *Applied Surface Science* 2021 [86]
**Type of nanostructure: three-dimensional templates and flexible/paper matrix**						
AgNPs synthesized in situ into bacterial cellulose membrane films obtained from commercial “Nata de Coco”. Label-free approach utilizing passive physical adsorption and electrostatic trapping of vesicles.	R6G for characterization; sEVs	Exosomes were purified from cultures of the healthy line MCF-10A and the aggressive tumor line MDA-MB-231. Raman spectra were measured: *n* = 18 (healthy), *n* = 20 (tumorous), and *n* = 13 (PBS control).	Analytical performance - LOD: 10–11 M for R6G, EF: 5.2 × 10^5^ (citrate) and 7.3 × 10^4^ (green synthesis) - Exploratory separation by PCA. - Twenty measurements on different samples/points for intra/inter sample reproducibility; RSD: N.R. - Correct classification of tumor exosomes with 95% confidence by PCA (PC1 and PC2 explain 96.25% of the total variance). Reproducibility Repeatability was analyzed in 20 different spot measurements. Citrate synthesis drastically reduces background noise and autofluorescence of bacterial cellulose.	- Biotechnological exploitation of a waste food resource (coconut cream) to achieve an extremely cheap active and reproducible SERS substrate. - Demonstrate that multivariate chemometric analysis applied directly to native exosome spectra can discriminate breast cancer subtypes without capturing antibodies, drastically reducing sample preparation costs. - Citrate acts as a growth inhibitor in the Ag(111) plane to induce anisotropic nanoparticles and cover cellulose peaks. Limitation: “Green” substrates without citrate have lower EF and high cellulose interference/autoflucescence.	- Cellular/analytical preclinical. - No patient exosomes; effective biological size and independent lots unclear. - Exploratory PCA without external testing; autofluorescence/cellulose signals may interfere. - Blind validation where tumor and healthy test samples fell exactly within the 95% confidence ellipses of the PCA calibration model.	76 *ACS Sensors* 2019 [8]
Quantum-scale structured (Q-structured) template of self-assembled TiO_x_ with oxygen vacancies introduced by multiphoton ionization. Label-free. Detection of breast cancer EGFR peptides and native cellular spectral signatures free of heating.	CV (characterization); EGFR and cellular Raman fingerprinting.	MDA-MB-231 cells (breast cancer), HeLa (cervical) and NIH3T3 fibroblasts (control). Number of independent biological cultures: N.R. Triplicate measurements in three samples (total *n* = 9 measurements averaged for calculation of EF).	Analytical performance - LOD: 10^−9^ M for CV; EF: 3.5 × 10^5^ (TiO_x_, 785 nm). - The cellular test was qualitative. Stability against laser exposure was evaluated for 120 cycles; RSD and variation between lots/substrates: N.R. - Quantitative differentiation of cancer cells by higher lipid content in region 2600–2800 cm^−1^ and EGFR peaks at 1134, 1242–1284 and 1566–1610 cm^−1^. Reproducibility High structural stability and optical inertia. The laser does not alter or deteriorate the positions of the substrate’s native Raman peaks after 120 cycles (360 s laser exposure).	- Non-plasmonic TiO_x_ rivaling noble metals. - First report of the use of oxygen vacancies incorporated into 3D quantum TiO_x_ to achieve a SERS substrate with semiconducting properties rivaling traditional plasmonic metals. - It brilliantly solves the problem of local plasmonic heating (which degrades or pyrolyzes proteins). Limitation: Evaluated only in a physiological environment at pH 7. Formal PCA statistical modeling is required to comprehensively discern HeLa lines.	- Cellular preclinical. - No human samples or diagnostic accuracy; EF and LOD were determined with dye; reproducibility between batches, days and instruments was not evaluated. - Excellent biocompatibility. - Direct cell culture on the template shows no cytotoxicity after 24 and 48 h (average viability of ~80%). - Visualization by SEM microscopy and fluorescent staining with DAPI/FITC.	69 *Nanoscale Horizons* 2019 [7]
Branched gold nanoparticles (nanostars and nanoraspberries) decorated onto flexible electrospun polymeric nanofibers. Surface modification with 4-MBA. Determination of intracellular pH (pHi) and extracellular pH gradients (pHe) in breast tumors with 4-MBA intracellular and extracellular pH gradients.	4-MBA; Intracellular and extracellular pH gradients	- MCF-7 cells (less invasive) and triple-negative MDA-MB-231 cells (highly aggressive and metastatic). - Six sensor fibers analyzed for reproducibility. - pHe gradients analyzed at 14 different positions with triplicate mapping. - No patients.	Analytical performance - Linear sensitivity in the physiological pH range (6.5–9.5); 0.68 nM, LOD and EF not applicable as the main criterion for pH measurement. - RSD ≈7% for absolute intensity and 4% for internal ratio; stability after reversible cycles; evaluation with six tips/fibers. - Average pHi determined in MCF-7 of 7.26 ± 0.27. - Identification of variable pHe gradients (0.1 to 0.9) in MDA-MB-231 as a function of distance (25 to 500 μm) Reproducibiilty Excellent reversibility and cyclic stability in 7 reversible cycles. RSD of only 4% for pH ratio (I_max_/I_1068_) and 7% in absolute intensity. No interference by cations or amino acids in the calibration. Ultra-fast response (<1 min).	- Fabricated a flexible, biocompatible cellular scaffold that monitors tumor acidosis in real-time and 3D, a critical parameter directly correlated with therapeutic resistance and metastatic potential. - The lens effect of the nanofiber enhances the electromagnetic field at the tip. Minimally invasive thanks to nanofiber tip diameters up to 60 nm. Limitation: Fluctuating pH and cellular dynamics can introduce noise; sporadic peaks in calibration at very low pH (<5.5).	- Real-time measurements on single live cells in vitro using optical micromanipulators and SERS fiber insertion (45° angle) controlled by magnetic holders and Raman immersion microscopy. - COMSOL Multiphysics simulates the electric field distribution of the hotspot in the nanostar. - Preclinical cell/physiological sensor. - It is not a study of diagnostic accuracy; few cell lines and biological units; in vivo applicability, batch-to-batch variation and inter-instrumental reproducibility not evaluated.	72 *ACS Sensors* 2020 [93]
Bimetallic cellulose strip (analytical filter paper) with silver core-gold shell (Ag@Au) nanoparticles structured in a “raspberry” shape directly synthesized in Whatman cellulose by SILAR method and wax printing. Passive capture driven by paper porosity and capillarity. SERS Label-free. Rapid POCT diagnosis of aquatic toxins (aniline, sodium azide, malachite green) and progression of breast cancer in urine.	R6G; FA, FCC, non-invasive carcinoma (stage 0) and invasive carcinoma (stage 1–3). Creatinine and tryptophan were proposed as urinary bands associated with tumor progression.	- Urine from four groups: fibroadenoma, fibrocystic change, non-invasive carcinoma and invasive carcinoma. - Mixtures of contaminants and real clinical urine samples from patients with benign (FA, FCC) and malignant tumors (non-invasive stage 0, and invasive stages 1–3). - Duplicate measurements/averaged at 10 random points per sample. - Number of participants per group: NR.	Analytical performance - LOD: 10^−15^ M and EF: 3.98 × 10^8^ for R6G. - LOD in mixtures: 10^−6^ M for aniline, 10^−9^ M for sodium azide and 100 pM for malachite green, with quantitative accuracy greater than 92–99%. - Descriptive urinary analysis by intensities at 850 and 1465 cm^−1^. - RSD: 6.1% on 10 strips; degradation < 10% at 30 days. - Identification and monitoring of invasive urinary tumor progression using bands of creatinine (850 cm^−1^) and tryptophan (1465 cm^−1^). Reproducibility Perfect spectral consistency with an RSD of only 6.1% in 10 independent strips. The discontinuous Au shell reduces the oxidation rate and oxidation decay of core silver to less than 10% in 30 days, in contrast to the 75% decay of monometallic AgNPs.	- Flexible, economical, and biodegradable paper test strip with direct layer-by-layer deposition using SILAR, free of complex colloid linkers. - Gold antioxidant protection on a plasmonic-optimized silver core. Limitation: Discontinuous structure of the “raspberry” shell, providing long-term incomplete antioxidant protection compared with a dense continuous shell.	- Exploratory clinical feasibility. - Clinical sample size, selection criteria, blinding, and diagnostic accuracy not reported; proposed biomarkers without independent validation; R6G LOD does not equate to clinical performance. - Extensively validated with synthetic toxicity mixtures and a clinical cohort of urine from real breast cancer patients. Theoretical verification of LSPR using COMSOL Multiphysics by FEM.	55 *Analytical Chemistry* 2017 [91]

TCs: Total Citations; LOD: limit of detection; EF: enhancement factor; N.R.: not reported; RBTIC: Rhodamine B Isothiocyanate; MGTIC: Malachite Green Isothiocyanate; DTDC: 3,3′-Diethylthiadicarbocyanine Iodide; R6G: Rhodamine 6G; 4MBA: 4-Mercaptobenzoic Acid; EpCAM: Anti-Epithelial Cell Adhesion Molecule; ErbB2: Anti-Erythroblastic Oncogene B2; CD44: Anti-Cluster of Differentiation 44; EGFR: Epidermal Growth Factor Receptor; CTCs: Circulating Tumor Cells; PD-L1: Programmed Death-Ligand 1; sEVs: Small Extracellular Vesicles; 4-mp: 4-Mercaptopyridine; CV: Crystal Violet; MB: methylene blue; FA: Fibroadenoma; FCC: Fibrocystic Change; AR: Alizarin Red; PD: Polydopamine; GaN: Gallium Nitride; FAM: Fluorescent Molecular Tag; SILAR: Successive Ionic Layer Adsorption and Reaction; FEM: Finite Element Model. Note: EF, LOD, and reproducibility values describe the analytical performance of each SERS platform. Citation counts (TC) reflect bibliometric visibility but do not necessarily indicate methodological rigor, diagnostic accuracy, or clinical utility.

### 3.6. Bio-Functionalization Strategies for Single, Dual, and Multiple Biomarker Recognition Through SERS

The bibliometric mapping reveals that bio-functionalization has emerged as the second major axis of innovation in SERS-based breast cancer detection, complementing advances in nanostructural engineering. While nanostructured materials primarily enhance sensitivity through electromagnetic and chemical enhancement, bio-functionalization improves selectivity, multiplexing capability and biological relevance.

The reviewed literature identifies five main bio-functionalization strategies: (i) antibody-based functionalization, (ii) aptamer–based functionalization, (iii) DNA/RNA probe functionalization, (iv) protein/enzyme activity probes, and (v) other innovative synthetic receptors. Together, these approaches expand the diagnostic potential of SERS by enabling more specific recognition of breast cancer biomarkers and improving analytical performance.

Recent studies highlight a shift from single antibody-based recognition toward multiplexed and biomimetic bio-functionalization strategies in SERS, as shown in Table 4. Antibody-functionalized probes have been used to detect breast cancer-associated receptors, angiogenic factors, and immune mediators [102,103,104,105]. In parallel, aptamers and peptides, including MUC1, AS1411, RGD, and iRGD, have enabled complementary targeting and phenotypic characterization of different breast cancer cell types [106,107]. Other emerging approaches include hyaluronic acid-CD44 recognition, m6A-sensitive DNAzymes for epitranscriptomic analysis, and tumor cell membrane-coated nanoparticles for homotypic targeting and tumor-margin delineation [69,70,108,109]. Collectively, these advances extend SERS beyond single-biomarker detection, enabling multidimensional characterization of breast cancer cells and their microenvironment.

To facilitate discussion, these strategies are organized into the following five subsections.

#### 3.6.1. Antibody-Based Functionalization

Antibodies, Y-shaped glycoproteins produced by the immune system, remain the most widely used recognition elements in SERS biosensors, due to their strong binding affinity and clinical familiarity. They are typically immobilized on plasmonic substrates or nanoparticles to capture target biomarkers. However, random immobilization often masks antigen binding sites, reducing efficiency. To overcome this, directional conjugation strategies such as Fc region binding via Protein G (PG), PEG linkers, or hydrazone chemistry maximize accessibility and reproducibility.

Examples include Cheng et al. (2023), who designed a raspberry-shaped dual-mode immune-nanoprobe conjugated with anti-EGFR antibodies, enabling in situ monitoring of EGFR expression after drug treatment, revealing that rosiglitazone reduced EGFR while metformin unexpectedly increased it [110]. Similarly, Kapara et al. (2020) functionalized AuNPs with anti-ERα antibodies via carbodiimide chemistry (with EDC-NHS-HS-PEG5000-COOH), allowing single-cell mapping of estrogen receptor α (ERα) expression and fulvestrant activity [111]. Extending this approach, Kapara et al. (2021) combined ERα-AuNPs and HER2-AuNPs for multiplexed characterization in 3D tumor spheroids, highlighting the role of antibody functionalization in distinguishing receptor profiles in complex microenvironments [112]. In other research, authors presented a dual-targeted SERS system based on transferrin-modified niosomes encapsulating AuNPs and Fe*_x_*O*_y_* NPs for MCF-7 detection, with 5-thio2-nitrobenzoic acid (TNB) as a Raman reporter [113].

HER2 detection has been a major focus; Spaziani et al. (2024) used AuNPs functionalized through an amine coupling reaction (EDC: NHS) with trastuzumab and 4-MBA as a reporter to classify HER2-positive and silenced breast cancer cell lines, enabling quantitative evaluation and subtype classification [13]. Téllez-Plancarte et al. (2018) immobilized trastuzumab on silver nanostructured plates via 4-MBA self-assembled monolayers (SAMs), achieving HER2 heterogeneity mapping across four cell lines [114]. Miser-Salihoğlu et al. (2025) extended antibody functionalization to BRCA1 quantification in tissue samples using magnetic nanoparticles, achieving a logarithmic correlation between protein concentration and Raman intensity (R^2^ = 0.9928), validating sensitivity and reproducibility [115].

Advances in orientation chemistry, such as those by Khosroshahi et al. (2023, 2024), demonstrated how hydrazone linkers improve reproducibility and sensitivity in HER2 and CA15-3 detection [76,116,117]. A similar strategy was applied by Piñeiro et al. (2026), presenting oriented anti-CD81 antibody conjugation on Au nanostars to track extracellular vesicle (EV) release from MCF-7 [118]. To address the random orientation on nanoparticle surfaces and hinder antigen recognition efficiency, the authors reported an oriented conjugation strategy using PG, which binds antibodies through their central region (Fc) while maintaining variable region (Fab) availability. Better reproducibility was obtained in a developed glass-based biosensor, which immobilized and oriented antibody-conjugated nanourchins for HER2 detection, achieving an EF of 3.5 × 10^4^ [76]. From the same group of research, a PCB-based platform further extended oriented immobilization to thin gold films and nanourchins, achieving EF up to 3 × 10^6^ [117]. These studies underscore how substrate-based immobilization can overcome reproducibility challenges inherent to colloidal systems. Other antibody-based innovations include Zhu et al. (2022), who reported interference-free AuNP probes conjugated with MT1-MMP antibodies, enabling clean detection of protease activity [119].

Multiplexed immunophenotyping has also been achieved with thiolated AuNPs-silica coating, labeled with distinct Raman reporters (BPE, 44AP, PHTH, TBBT) [120], and developed tetraplex SERS nanotags for simultaneous profiling of HER2, ER, and PR [121]. Meanwhile, M. Li et al. (2019) introduced alkyne/nitrile Raman reporters in the Raman-silent region, conjugated with antibodies against ER, PR and EGFR, avoiding spectral overlap and enabling multiplex imaging [122].

Theranostic applications further extend antibody functionalization, combining targeted therapy with imaging. For instance, Narayan et al. (2021) developed cucurbituril-gold nanorod assemblies loaded with methylene blue and conjugated with anti-HER2 antibodies through CH3-PEG-SH and EDC:NHS, providing targeted phototherapy (photothermal (PTT) + photodynamic (PDT)) and SERS imaging [77]. Also, Parchur et al. (2016) presented multimodal imaging (fluorescence and SERS) and photothermal therapy, with Prussian blue (PB)-functionalized hybrid nanostructures (CaMoO_4_:Eu@SiO_4_@Au nanorod) conjugated with oriented anti-HER2 antibodies [12]. A similar functional concept was proposed by Hu et. al (2025) [123]. They prepared antibody-functionalized hexoctahedral Au nanoparticles for EpCAM/EGFR detection, with an LOD of 10^─12^ M and EF of 3.8 × 10^8^, because the antibodies are conjugated to plasmonic nanoparticles carrying Raman reporters and fluorophores, ensuring highly selective recognition with improved sensitivity and reproducibility. Another proposal to combine theranostics is presented by Nima et al. (2017), who used silver-decorated Au nanorods functionalized with anti-EpCAM antibodies and loaded with doxorubicin for targeted drug delivery and intracellular SERS monitoring [124].

With an innovative proposal, L. Li et al. (2019) introduced ratiometric SERS nanoprobes conjugated with urokinase plasminogen activation receptor and EGFR-targeting peptides, enabling quantitative discrimination of biomarker expression in single cells [125]. Lately, Miao et al. (2026) have considered two new variables, space and time, studying how the biomarkers change as the cancer evolves [126]. For this work, the authors reported a dual-targeted sensing platform using IO NPs@AR@PDA@aPD-L1 and Au NPs@4-MBA@PDA@aHER2 bioprobes with PD-L1 and HER2 antibodies, enabling mapping of therapeutic markers during breast cancer progression. Collectively, antibody-based strategies highlight the balance between specificity, orientation, and multiplexing, making them central to translational diagnostics.

#### 3.6.2. Aptamer-Based Functionalization

Aptamers, short single-stranded DNA or RNA oligonucleotides selected through Systemic Evolution of Ligands by Exponential Enrichment (SELEX), offer synthetic consistency, chemical stability, and flexible structures that access epitopes that may be sterically hindered for antibodies. Their small size and ease of engineering make them ideal for multiplex detection.

For example, Yarbakht et al. (2018) used Fe_3_O_4_@Au nanoparticles functionalized with MUC1 aptamers for selectively isolating breast cancer cells, while aptamer-functionalized AuNPs with 4-mercaptopyridine (4-Mpy) [127]. Xia et al. (2026) advanced this approach by combining aptamer probes with Machine learning (Random Forest) for classification, achieving 96% accuracy and 93.7% specificity, with an ultralow LOD of 2.96 fg/mL for MUC1 [32]. Hybrid biosensors integrating aptamers and antibodies, such as those by T. Another biofunctionalization is reported by Oliveira et al. (2024), who combined molecularly imprinted polymers with antibody-functionalized Au NSs for CA15-3 detection [4]. Aptamer-like selectivity was achieved through MIPs, while antibody-AuNSs conjugates amplified SERS signals, supporting point-of-care applications.

Innovative designs include Shen et al. (2021), who synthesized PB analogue (PBA) AuNPs functionalized with aptamers through EDC:NHS for multiple detection of EpCAM and EGFR [75], and Xia et al. (2025), who developed logic-gated nanoplatforms for dual biomarker presence (APE1 and miR-155) to reduce false positives [128]. Aptamer-based functionalization thus provides synthetic reliability, multiplexing capacity, and theranostic potential, complementing antibody systems.

#### 3.6.3. DNA/RNA Probe Functionalization

DNA and RNA probes exploit sequence-specific hybridization to detect genetic and epigenetic biomarkers. Amplification strategies such as catalytic hairpin assembly (CHA), Exonuclease III (Exo III), duplex-specific nuclease (DSN) recycling, rolling circle amplification (RCA), and tyramine signal amplification (TSA) consistently push detection limits into the femtomolar or attomolar range, enabling early detection of low-abundant biomarkers.

For instance, Ge et al. (2021) integrated RCA with Au@SiO_2_ arrays to detect CpG methyltransferase (M.SssI) activity (EF of 7.49 × 10^6^ and a LOD of 2.51 × 10^−4^ U/mL), providing insight into DNA methylation [129]. Indeed, Zhao et al. (2022) presented two amplification strategies, RCA and TSA, for the exosomal targeting of miR-21 in a microfluidic chip, reaching an LOD of 1 pM [28]. H.-S. Tan et al. (2024) and Meng et al. (2021) employed dual signal calibration and holography chips for miRNA detection through hairpin DNA functionalization and hairpin and Exo III amplification, respectively, achieving femtomolar sensitivity and rapid multiplexing [40,130]. Weng et al. (2022) integrated CHA amplification with Ag@Au core–shell nanoparticles for the detection of miR-21 (LOD: 0.398 fM) and miR-155 (0.215 fM) in breast cancer serum [131]. Pang et al. (2016) and Xu et al. (2023) used DSN amplification for miRNA profiling, with Fe_3_O_4_@Ag reaching an LOD of 0.3 fM for let-7b, and Au@Ag core–shell nanorods to identify miR-21, miR-155, and let-7b at LODs of 0.05–0.063 fM [17,132].

Other designs introduced miRNA-detectable (miR-200c) nanoparticles for intracellular profiling [133]. Zhenxing Wang et al. (2019) demonstrated enzyme-free strand displacement recycling in a microfluidic platform, achieving femtomolar detection in miR-21 [16].

Then, Eom et al. (2017) engineered nanogap-rich gold nanowires for ultrasensitive detection of telomerase activity, achieving attomolar detection limits, among the lowest reported for this biomarker [10]. Hou et al. (2025) developed SERS nanotags for dual detection of BRCA1 and BRCA2, achieving detection limits of 0.61 pM and 0.78 pM [134]. Collectively, DNA/RNA probes provide mechanistic insights, multiplexed profiling, and therapeutic monitoring, confirming their pivotal role in precision oncology.

#### 3.6.4. Protein/Enzyme Activity Probes

Protein and enzyme activity probes monitor dynamic biochemical processes rather than static biomarkers, offering real-time functional readouts of tumor progression, invasion, and therapeutic response. These systems employ peptide substrates, enzyme-responsive chemistry, or ratiometric designs to achieve quantitative detection.

Shashni et al. (2017) targeted hypersialylation with phenylboronic acid PEGylated AuNPs, linking glycan metabolism to metastasis [135]. Zhong et al. (2022) and Zhu et al. (2025) probed MMP-2 activity using ratiometric nanoprobes and core–satellite substrates, respectively, highlighting the importance of internal standards for reproducibility [31,136]. J. Wang et al. (2023) integrated microdroplet microfluidics with magnetic SERS probes to profile extracellular vesicle proteins (EpCAM, HER2, EGFR) at the single-cell level, revealing heterogeneity [39]. He et al. (2018) monitored Protein Kinase A with AuNSs functionalized with kemptide substrates, extending SERS into intracellular signaling pathways [137]. This study demonstrates how protein/enzyme probes expand SERS into functional diagnostics by capturing enzymatic activity and signaling events critical to cancer biology.

#### 3.6.5. Other Innovative Functionalization Approaches

This section highlights several non-conventional functionalization strategies that broaden the scope of SERS biosensing beyond antibodies, aptamers, or nucleic acid probes. Each study introduces a unique way to functionalize nanostructures, targeting glycans, receptors, stem cells, or even organelles, and, together, they illustrate how innovation in probe design can expand diagnostic and therapeutic applications.

Jana et al. (2023) and Liang et al. (2018) focused on sialic acid detection, a glycan biomarker linked to metastasis [138,139]. Jana et al. introduced a fluorescence–SERS dual-enabled simultaneous quantitative detection and spatial imaging of glycans at the single-cell level with AuNPs [138], while Liang et al. used MPBA@AgNP to distinguish cancerous from normal tissues spectroscopically [139]. Haldavnekar et al. (2020) explored cancer stem cell (CSC) surveillance using nickel-based nanoprobes [140]. Their system achieved ~99% accuracy in distinguishing CSCs, offering a powerful prognostic tool. These innovative approaches collectively expand SERS from biomarker detection to real-time monitoring of cancer progression and treatment efficacy, with improvements in dual-mode imaging, enhanced stability, 3D architectures, and intracellular targeting.

The studies analyzed indicate that the sensitivity and diagnostic performance of SERS platforms are strongly governed by the characteristics of the employed nanoarchitecture, particularly by its capacity to generate, distribute, and stabilize plasmonic hotspots. Simpler nanostructures, such as spherical gold or silver nanoparticles, offer relatively straightforward and reproducible fabrication, although they typically provide moderate enhancement factors. In contrast, more sophisticated architectures, such as gold nanopillars, nanogap-rich nanowires, multiplexed nanotags, gold nanostars, and three-dimensional plasmonic platforms, substantially increase the density of localized electromagnetic fields and enable LOD in the femtomolar or even attomolar range. However, greater structural complexity does not necessarily guarantee improved diagnostic capability or a higher degree of clinical validation.

This apparent paradox arises because analytical sensitivity constitutes only one of the components determining the overall performance of a SERS platform. Nanoarchitectures with extraordinary EF may nonetheless present limitations related to fabrication reproducibility, spatial signal uniformity, hotspot stability, or scalability for clinical applications. Conversely, some relatively simple platforms have demonstrated greater translational potential owing to their robustness, reproducibility, and compatibility with complex clinical samples.

Biofunctionalization plays an equally decisive role. Strategies based on antibodies, aptamers, DNA/LNA probes, or exosome-capture systems enable the physical amplification generated by the nanoarchitecture to be converted into specific molecular recognition. In these systems, diagnostic specificity depends both on the quality of the bioreceptor and on the substrate’s capacity to uniformly amplify the signal arising from the biomolecular interaction. However, high specificity is accompanied by new challenges related to bioreceptor stability, variability in conjugation processes, and the need to select biomarkers of genuine clinical relevance.

Label-free platforms, by contrast, represent a fundamentally different philosophy. Rather than targeting individual biomarkers, they exploit the global molecular fingerprint of cells, tissues, serum, or exosomes. In these cases, complexity shifts from biofunctionalization toward computational analysis. While highly biofunctionalized systems tend to generate more targeted signals that can be analyzed using conventional chemometric tools such as PCA-LDA or PLS-DA, label-free strategies produce high-dimensional spectra that require more advanced ML and deep learning methods, including Random Forest, SVM, XGBoost, CNN, or Transformer-based models, as shown in Section 3.8.

This relationship suggests a technological gradient in which the support for AI increases as the intrinsic molecular specificity of the system decreases. Consequently, diagnostic capability does not depend exclusively on the nanoarchitecture or on the algorithm employed, but on the interaction between both. A highly complex nanoarchitecture may generate large volumes of spectral information, but without an adequate computational strategy, that information remains unexploited. Likewise, sophisticated algorithms are unlikely to compensate for limitations arising from poorly reproducible substrates or inconsistent biofunctionalization.

For this reason, the future evolution of SERS in breast cancer research should move toward co-design strategies that simultaneously integrate nanoarchitecture, biofunctionalization, and computational analysis. The evaluation of new platforms should incorporate not only classical sensitivity and LOD parameters, but also metrics of reproducibility, stability, model interpretability, robustness to inter-patient variability, and multicenter external validation. From this perspective, the future of SERS does not appear to depend on developing progressively more complex nanostructures, but rather on optimizing the balance between plasmonic amplification, molecular recognition, and analytical capability to yield reproducible, scalable, and clinically useful systems.

**Table 4:** Synthesis matrix of bio-functionalization strategies and signal amplification mechanisms.

Biological Target	Principal SERS Architecture	Functionalization and Amplification Strategy	Main Findings (Innovation)	Main Application	AP and RE	Validation Level	TC Reference
microRNA let-7b	Fe_3_O_4_@Ag magnetic core–shell nanoparticles bearing Raman-tagged DNA probes	DSN, catalytic cleavage of DNA/RNA heteroduplexes	Enables ultra-low detection of miRNA by target recycling and signal amplification	Cellular miRNA analysis/potential liquid biopsy	AP LOD: 0.3 fM (15 zeptomole, 50 μL) RE Spike-recovery 97–103%, selectivity against let-7 family reported	Level 1—cell-derived material	200 [17]
miRNA-21/miRNA-155	2D Au-Si substrate + Ag@4-MBA@Au core–shell nanotags (sandwich chip)	CHA, enzyme-free DNA metastable hairpin cascades	Dual-miRNA assay linked analytical amplification to clinical serum discrimination	BC diagnosis/prognosis by liquid biopsy	AP LOD: 0.398 fM (miR-21) and 0.215 fM (miR-155); range 1 fM–10 nM; reported accuracy 100% RE 30 BC + 30 healthy sera; repeatability and specificity assessed; no external cohort	Level 2—single-center clinical cohort	81 [131]
Telomerase activity	Nanogap-rich Au nanowires formed by Au nanoparticle deposition	Telomeric-substrate primer elongation, G-quadruplex folding and methylene blue intercalation	Directly detects enzymatic hyperactivity in gastric and breast tissue extracts	Gastric/breast cancer tissue assessment	AP LOD: 0.2 cancer cells/mL RE Substrate RSD 4.80%; tested in multiple cell lines and 16 tumor-bearing mice; no human cohort	Level 1—cell/animal tissue	112 [10]
Extracellular Protein kinase A (PKA)	Star-shaped gold nanoparticles. BSA-kemptide-functionalized Au nanostars	Label-free peptide phosphorylation. Ratiometric I725/I1395 index assisted by PCA	Achieves quantitative phenotyping of phosphorylation states without external tags	Cancer-cell phenotyping/kinase assay	AP LOD: 5 mU/mL RE 12 samples measured in triplicate (*n* = 36); selectivity against other kinases assessed	Level 1—in vitro/cell-line study	69 [137]
Cancer-cell surface receptors multiplex discrimination	SERS-encoded gold NSs with embedded Raman reporters	Multiplexed reporter encoding. Deep trapping of Raman dyes paired with cellular targeting	Allows high-throughput, algorithmic storage and discrimination of complex cancer cell populations	Multiplex cancer-cell imaging/discrimination	AP Nanotag detection limits characterized; no clinical sensitivity/specificity reported RE Multiple reporters/cell models and mapping experiments; no patient validation	Level 1—cell-line study	171 [54]
Intracellular targets. uPAR and EGFR on BC cells	Ratiometric nanoprobes. Two peptide-targeted, spectrally encoded Au SERS nanoprobes	Internal reference calibration. Dual signal functionalization to cancel out intracellular noise	Internal ratio reduces uptake/illumination variability and discriminates MDA-MB-231 from MCF-7 cells	BC cell screening	AP Cell discrimination demonstrated; LOD, sensitivity and specificity not reported RE Single-live-cell imaging with two cell lines; no clinical cohort	Level 1—cell-line study	60 [125]
Multiple cell/tissue biomarkers	Au NPs carrying alkyne- or nitrile-bearing Raman reporters	Raman-silent-region reporters (1800–2800 cm^−1^) that also anchor to Au; antibody conjugation	Expands multiplex tag chemistry beyond conventional thiols and minimizes biological spectral background	Multicolor imaging in cancer cells and human breast tissues	AP Qualitative multiplex imaging; no LOD or diagnostic accuracy reported RE Cell experiments and clinical tissue imaging reported; number of tissue donors not stated in supplied article	Level 2—exploratory clinical tissue	58 [122]
DNA-drug interactions. BRCA1 dsDNA-doxorubicin interaction	Au electrode/SAM/rGO/Fe_2_Ni@Au magnetic nanoparticles functionalized with BRCA1 dsDNA	Multimodal interface tracking. Dual readouts of structural intercalation and electrical impedance	Reveals intricate binding kinetics between anticancer chemotherapeutics and native DNA	Preclinical anticancer-drug screening	AP LOD: 8 μg/mL of DOX; semilog linearity over 3 orders RE Dose–response and complementary SERS/electrochemical signals; purified DNA only	Level 0—analytical proof of concept	167 [65]
Exosomal miR-21, miR-222 and miR-200c	Uniform plasmonic head-flocked Au nanopillars	Locked-nucleic-acid probes; direct hybridization without amplification; multiplex detection	Uniform hotspots provide attomolar, single-nucleotide-specific detection and subtype-related expression patterns	Exosomal-miRNA profiling	AP LOD: 1 aM; range 1 aM–100 nM; recovery 97–102% RE Spot RSD 2.98%; probe RSD 4.65%; detection RSD 4.53%; spiked human serum and cell-line exosomes	Level 1—cell-derived/spiked serum	233 [59]
ER, PR and HER2 in FFPE tissue	Antibody-conjugated Raman-label AuNP SERS nanotags	Singleplex/duplex/triplex immunorecognition with ratiometric signatures	Multiplex spectro-histology and semi-quantitative HER2 grading in one tissue section	BC biomarker detection and grading	AP Sensitivity/specificity: 95/92% singleplex; 88/85% duplex; 75/67% triplex; 0.5–5 mm2 imaged within 45 min RE Compared with IHC/FISH in retrospective FFPE tissues; sample size limited and no multicenter validation	Level 2—retrospective clinical tissue	42 [141]
Serum biochemical fingerprint	Colloidal Ag NPs; coffee-ring deposition	Label-free SERS at coffee-ring margin versus center; multivariate classification	Shows sampling position materially affects serum classification performance	Breast cancer serum screening	AP Sensitivity 94.8%; specificity 98% (best sampling region/model) RE 109 serum samples (58 breast cancer, 51 healthy); LOOCV/k-fold internal validation; no independent cohort	Level 2—single-center clinical cohort	27 [142]
Primary normal epithelial vs. cancer cells	Citrate-reduced Ag NPs mixed with isolated single cells	Label-free cellular fingerprinting + Random Forest	Uses fresh patient-derived cells rather than established cell lines	Cell-level breast cancer discrimination	AP Accuracy 78.2%; precision 75.5%; recall 66.7% RE 135 spectra from cells of 15 patients; random 10-fold cross-validation risks patient-level leakage if spectra from	Level 2—small single-center clinical cohort	29 [143]
Cellular HER2 expression	Label-free cells on plasmonic Au NSs substrate	No affinity label; ALS/threshold segmentation + PCA/chemometrics and LOOCV	Enables longitudinal, cellular-level HER2 surveillance during targeted treatment	Therapeutic monitoring of HER2-positive breast cancer	AP Accuracy/AUC reported as 0.996 for cell-line classification RE LOOCV on spectra from cell lines; no independent patients; biological generalizability remains untested	Level 1—cell-line study	28 [144]
Polysulfide/hypotaurine stromal fingerprints	Au NPs array for large-area tissue SERS imaging	Label-free spatial metabolomics aligned with H&E; XGBoost and comparator algorithms	Identifies a stromal redox signature of desmoplasia to distinguish DCIS from invasive cancer	Differential diagnosis of DCIS vs. invasive breast cancer	AP High classification performance reported; exact metric is model/split-dependent RE 46 patients (14 DCIS, 32 invasive); patient-level evaluation set after cross-validation; single center	Level 2—clinical cohort with held-out internal evaluation	10 [145]
Serum exosomal molecular fingerprints/BC subtype	Label-free exosomes adsorbed on a plasmonic SERS substrate	One-dimensional CNN classification; no affinity reporter	AI-based exosome fingerprinting supports diagnosis, subtype classification and postoperative assessment	Liquid-biopsy diagnosis and follow-up	AP 100% prediction accuracy for human patients RE 5 healthy + 34 breast cancer patients; 200 spectra/patient used for clinical prediction.	Level 2—small single-center clinical cohort	0 [144]
HER2-positive serum exosomes before/after NAT	Magnetic beads@HER2-Exos@HER2-SERS detection nanoprobes (HER2-MEDN)	Dual anti-HER2 capture/detection + PCA-LDA-SVM	Enriches HER2-positive exosomes and improves early prediction of neoadjuvant response	Early NAT-response prediction	AP Base Raman model AUC > 0.89; HER2-MEDN model accuracy > 0.94 RE 56 HER2-positive patients; 70/30 spectrum/data split; no independent external cohort	Level 2—single-center clinical cohort	22 [146]
Pretreatment serum fingerprint + clinicopathological features	Colloidal AgNP serum SERS	PCA + Transformer feature extraction and multimodal fusion	Combines molecular fingerprint and clinical variables for NAT-response prediction	Personalized prediction of neoadjuvant response	AP Accuracy 92.6% in cohort 1; independent cohort accuracy 90%, AUC 93% RE Cohort 1: 68 patients (204 spectra); independent cohort 2: 30 patients (90 spectra); double-blind external temporal/site validation reported	Level 3—independent validation cohort	3 [147]

AP: analytical performance; RE: reproducibility evidence; TCs: total citations; DSN: duplex-specific nuclease; LOD: Limit of Detection; CHA: catalytic hairpin assembly; NSs: nanostars; NPs: nanoparticles; DOX: doxorubicin.

### 3.7. Liquid Biopsy in Breast Cancer: Exosomes and Circulating Biomarkers

Historically, the definitive diagnosis and molecular characterization of breast cancer have relied on traditional tissue biopsies, including core needle biopsies and surgical excisions. Although tissue sampling remains the clinical gold standard, it is associated with significant limitations: the procedures are invasive, often painful, and provide only a static examination of the tumor microenvironment. Furthermore, tissue biopsies frequently fail to capture the full spatial and temporal heterogeneity of breast tumors, and their invasive nature makes them impractical for continuous, long-term monitoring of a patient’s response.

**Figure 8:**
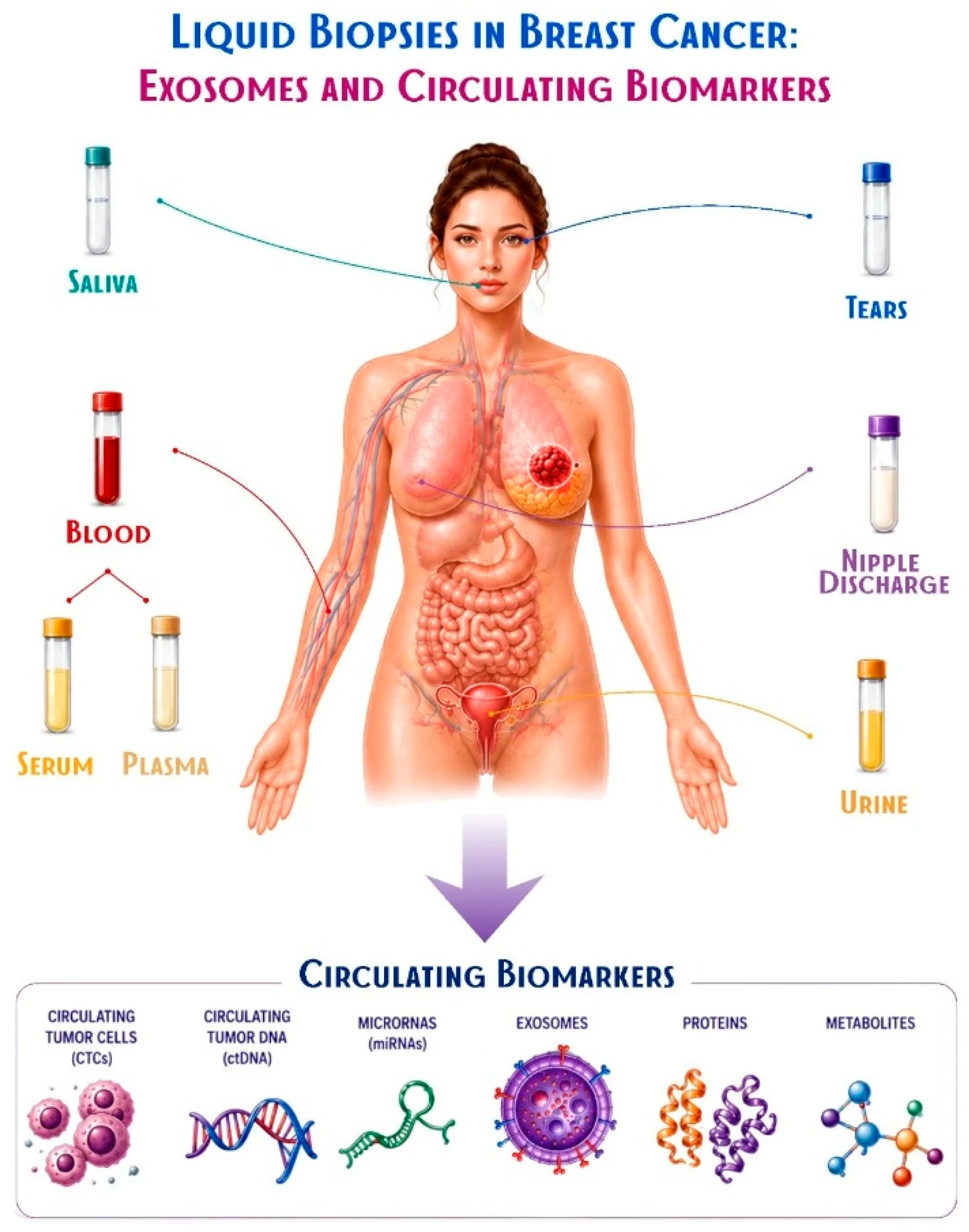
Schematic overview of liquid biopsy matrices and circulating biomarkers in breast cancer diagnostics.

To address these clinical bottlenecks, liquid biopsy has emerged as a transformative diagnostic paradigm, offering minimally invasive access to tumor-derived information using non-solid biological fluids, most commonly blood (plasma or serum), but increasingly expanding to highly accessible and non-invasive biofluids such as saliva, tears, urine, and nipple discharge (Figure 8).

Within the context of the structured literature search and the MCA, several articles were identified that align with the third thematic cluster, which focuses specifically on liquid samples, blood, serum and plasma, through the isolation, detection, and characterization of exosomes, circulating biomarkers, as well as alternative biofluids. In this context, SERS effectively bridges the gap between the clinical need for liquid biopsies and the technical capability to detect trace tumor-derived entities.

#### 3.7.1. Exosome-Based/Derived SERS Strategies

Exosomes have emerged as pivotal biomarkers for liquid biopsy in BC owing to their high abundance in biofluids, superior stability, and their ability to reflect the molecular status of their parental tumor cells [18]. Recent research trends highlight a significant shift toward the development of integrated SERS platforms capable of exosome isolation, multiplex detection, and label-free molecular fingerprinting, thereby enabling precise and clinical staging.

A central challenge in exosome-based diagnostics is the simultaneous quantification of multiple surface proteins to accurately identify cancer subtypes. To address this, Su et al. (2023) developed a paper-based vertical flow assay (VFA) biosensor employing AuNSs for quantitative detection of MUC1, HER2 and CEA in clinical serum [18]. Unlike conventional lateral flow assays, the VFA format minimizes the hook effect, a phenomenon in which excessively high analyte concentrations yield false-negative or attenuated signals. This system reaches an LOD in the range of 1–2.9 ×10^7^ particles/mL. Extending this approach, Su et al. (2023) reported an integrative lateral flow strip combined with multivariate spectral unmixing, enabling absolute quantification of SKBR and MCF exosome subtypes rather than single surface markers [62]. This method achieved an LOD of 1 × 10^6^ particles/mL and demonstrated utility in monitoring tumor reduction following surgical intervention. Further advancing multiplexing capabilities, G. Zhang et al. (2023) introduced a magnetically driven tandem chip that enables rapid isolation and comprehensive profiling of EVs by using cycling SERS tags to amplify signals from multiple protein biomarkers [148].

The specific isolation of tumor-derived exosomes, such as PDL-1, is critical for diagnostic accuracy. Su et al. (2025) introduce an immunomagnetic substrate modified with zwitterionic TMAO and PAMAM dendrimers and EpCAM aptamers (Fe_3_O_4_@PAMAM@TMAO@Aptamer), achieving a capture efficiency of 90.5% with minimal nonspecific protein adsorption [149]. Complementing these findings, the development of a nanophotonic immunoarray with electrochemically roughened surfaces provides a handheld approach for detecting secreted PD-L1 [150]. Similarly, Li et al. (2021) utilized gold nano-dot-immobilized magnetic nanoparticles (MNPs; MNPs@Au) with an EF of 225 to isolate and distinguish cell- and patient-derived exosomes using CD9 as a target [151]. In contrast, Zhou et al. (2023) proposed a non-selective trapping strategy using Ti3C2TxMXene, which forms Ti-O-P complexes with the exosomal lipid layer, to overcome the spatial site resistance of sandwich immunoassays [152]. This approach, combined with peptide-functionalized SERS tags, achieved an exceptionally low LOD of 20.74 particles/mL. Ngo et al. (2025) utilized silane-PEG-DSPE to anchor small extracellular vesicles (sEVs) onto glass surfaces through lipid bilayer integration, successfully detecting EpCAM-positive vesicles from patient plasma at concentrations as low as 1.6 × 10^7^ particles/mL [153].

Label-free strategies have also been implemented, providing an intrinsic “fingerprint” of the exosomal composition without the need for exogenous tags. For example, Wang et al. (2025) designed a 3D plasmonic AgNP@Au@PS nanosphere array chip that performs automated on-chip nanofiltration to shield SERS “hotspots” from interfering plasma proteins and salts [154]. This platform enabled accurate BC staging (stages 0–III) with a diagnostic accuracy of 99.6%. In terms of substrate innovation, Li et al. (2023) developed an electroplated platinum-black template, which offers high reproducibility and a near-zero background, distinguishing 4T1 cancer-derived exosomes with 83.3% sensitivity [155]. Furthermore, Zhao et al. (2025) engineered Ag@Na_7_PW_11_O_39_ (lacunary polyoxometalate architecture) nanoclusters that improve EM with interfacial charge transfer, achieving an LOD of 7.73 × 10^7^ particles/mL and an EF of 5.6 signal increase for exosomal protein detection in a nanozyme-linked immunosorbent assay [11].

Beyond protein profiling, nucleic acid-based exosomal biomarkers have been targeted with multiplexed SERS strategies. Hou et al. (2022) developed an asymmetric Au@Au@Ag probe system for simultaneous detection of multiple exosomal microRNAs [156]. By leveraging enhanced Raman signals and spectral separation, this approach achieved highly sensitive multiplexing, underscoring the potential of exosomal miRNAs as robust diagnostic indicators. Complementing this, Lee et al. (2019) [59] introduced a plasmonic head-flocked Au nanopillar sensor for quantitative and specific detection of exosomal miRNAs. This architecture provided strong electromagnetic field enhancement and reproducibility, enabling accurate BC diagnosis [59]. Similarly, Q. Zhang et al. (2023) [55] reported a ratiometric SERS biosensor based on an Au@Ag/GO substrate and V-shaped double-stranded DNA probes for precise identification of BC-derived exosomes. By employing 4-NTP as an internal standard, the system mitigated environmental fluctuations and achieved an LOD of 150 particles/mL [55].

Previous studies, as mentioned before, are capable of staged BC for 0–III; however, the technique of Feng et al. (2025) reported on healthy donors and BC patients across all clinical stages (0–IV) with an accuracy of 89.7%, using an array integrating an amphiphile-dendrimer supramolecular probe on a nitrocellulose membrane for capturing sEVs, with AuNP-based nanotags targeting CD9, EpCAM, and HER2, including machine learning [24]. Then, a proposal for rapid clinical screening from Ho et al. (2024) introduced a droplet microfluidic platform for detecting HER2-positive exosomes [157]. The system leveraged salt-induced gold nanoparticle aggregation triggered by the presence of targeted exosomes, achieving an LOD of 4.5_log10_ particles/mL in five minutes.

Exosome-based SERS platforms have advanced significantly, combining immunomagnetic capture, plasmonic nanostructures, nucleic acid probes and label-free fingerprints to achieve highly sensitive, multiplexed, and reproducible detection. These innovations enable precise molecular subtyping and staging of BC, positioning SERS assays on exosomes as robust, non-invasive tools with strong translational potential for clinical diagnostics.

#### 3.7.2. Circulating Tumor Cells (CTCs)

In the area of liquid biopsy for breast cancer, circulating tumor cells (CTCs) are recognized as high-value biomarkers for early diagnosis, prognostic assessment, and therapeutic monitoring. Their clinical implementation, however, is hindered by their extreme rarity, often present at concentrations as low as 1–10 cells/mL of blood, and by substantial phenotypic heterogeneity. These challenges necessitate the development of isolation and detection platforms with ultra-high sensitivity and advanced multiplexing capabilities.

Recent research emphasizes the need to move beyond single-marker detection to capture heterogeneous CTC subpopulations, particularly those that evade EpCAM-based assays. Key strategy advancements include those reported by Wilson et al. (2020) [158], who integrated a microfluidic platform employing magneti–optical iron-oxide-Au core–shell SERS nanotags. This system enabled quantitative, simultaneous detection of four biomarkers (EpCAM, HER2, CD44, IGF1R) through immunomagnetic capture and spectral deconvolution using classic least-squares (CLS) regression, demonstrating strong concordance with gold-standard ELISA [158]. Similarly, Zhang et al. (2018) introduced an on-chip strategy combining size-based isolation with three spectrally orthogonal SERS aptamer nanovectors targeting EpCAM, HER2 and EGFR [64]. By applying revised-CLS and partial least-squares discriminant analysis (PLS-DA), they achieved 3D phenotypic profiling at single-cell resolution, enabling reliable classification of breast cancer subtypes (SKBR-3, MCF-7, MDA-MB-231).

To minimize sample loss, “all-in-one” devices have been proposed by Jibin et al. (2021) [159], developing a portable lab-on-a-filter centrifugal prototype capable of processing up to 5 mL of whole blood within 60 s. The report indicates that this system employed a dual-functional sandwich complex, an anti-EpCAM-functionalized polycarbonate filter combined with Au-rGO@anti-ErB2 nanohybrids, achieving a LOD of 5 cells/mL and providing block-free, highly selective detection in complex biological matrices [159].

Additional strategies have expanded the scope of CTC detection. Pan et al. (2025) reported a PD-L1-targeted iron oxide SERS bioprobe, enabling accurate detection of CTCs and the capacity for tumor boundary differentiation [160]. This approach demonstrated high specificity for PD-L1-positive cells without the use of noble metals and underscores its potential for monitoring expression in BC, including triple-negative BC. Szymborski et al. (2022) introduce a dielectrophoresis-based sensor integrated into a microfluidic chip, which allowed label-free detection of cancer cells with a threshold sensitivity as low as 20 cells/mL [161].

Related dual-function SERS identification platforms are being developed to enhance the precision of liquid biopsy for CTCs, providing a preliminary screening tool that complements exosomal analysis in comprehensive liquid biopsy frameworks. The platform presented by D. Zhang et al. (2023) is tailored for both preliminary screening and definitive diagnosis, using a magnetic Fe_3_O_4_ complex capable of capturing TNBC [162]. Building on this, Zhang et al. (2025) reported a dual-modal approach using A and IO bioprobes integrated with a Random Forest classifier [35]. This machine learning-assisted system achieved a 98% detection rate for CTCs, 90% classification accuracy across multiple tumor cell lines, and an ultra-sensitive LOD of 2 cells/mL, while effectively eliminating interference from peripheral leukocytes [35].

The rapid evolution of SERS-based biosensors, microfluidics, and machine learning integration has drastically enhanced the sensitivity and specificity of CTC detection. By successfully addressing the core challenge of tumor heterogeneity and extremely low cell concentrations, these advanced platforms provide a robust foundation for incorporating CTC analysis into routine clinical workflows. Finally, these technologies hold profound potential for non-invasive disease monitoring and highly personalized breast cancer management.

#### 3.7.3. Circulating Biomarkers

Human blood serves as a rich reservoir of systemic information, characterized by a complex composition of cellular remains, protein aggregates, lipoproteins, and diverse biochemical species in the plasma and serum. Within the paradigm of liquid biopsy for BC, CTCs and exosomes are traditionally considered the most prominent targets due to their direct cellular origin and the tumor-specific load they carry. Nevertheless, a substantial and growing number of other high-importance circulating biomarkers are equally vital for the comprehensive detection and monitoring of BC. These include proteins such as CA15-3, CA125, CEA, and HER2, as well as circulating nucleic acids like miRNAs, cell-free DNA (cfDNA) and metabolites. Furthermore, the tracking of circulating metabolites, including amino acids, lipids and nucleic acid catabolites, allows for the acquisition of a molecular fingerprint that reflects real-time pathologic transformations and therapeutic efficacy.

Regarding protein biomarkers, Zheng et al. (2018) developed a multiplexed microfluidic chip for the simultaneous detection of CA15-3, CA125 and CEA in human serum [61]. The system utilized self-assembled AgNPs, reaching an LOD of 0.01 U/mL for CA15-3/CA125 and 1 pg/mL for CEA. In a similar vein, Wang et al. (2019) targeted the chemokine IL-8, an important marker for tumor angiogenesis [163]. Their sandwich immunoassay utilized highly branched gold nanoparticles on ITO glass as the substrate and 4-MBA-labeled Au nanocages as SERS tags [163]. The study reported a linear response in human serum, identifying higher IL-8 levels in BC patients compared to healthy controls.

Also, in proteins but with directional antibody conjugation, Khosroshahi et al. (2022, 2025) presented a label-free immunosensor for HER-2 and CA15-3 using directional monoclonal antibodies conjugated to Au nanourchins immobilized on a printed circuit board substrate [164,165]. This dual-oriented target platform achieved an EF of 0.5 × 10^5^ and successfully quantified biomarkers in clinical serum samples with an LOD of 10 ng/mL (HER-2) and 20 U/mL (CA15-3) [164]. This work, along with related studies by the same group, such as the label-free multiplexed SERS microfluidic immunoassay and the optical characterization of CA15-3 using Au nanocluster probes, underscores the utility of GNU morphology and directional conjugation in maximizing biomarker capture and signal reproducibility [165].

Integrating the detection of multiple types of circulating biomarkers, Reza et al. (2018) reported a platform for the parallel profiling of cancer cells and proteins [166]. Their system consisted of AuNPs functionalized with Raman reporters and secondary antibodies targeting HER2 and MUC-16, reaching an LOD of 100 fg/mL.

Circulating miRNAs and small metabolites offer additional opportunities for diagnostic information. Zhang et al. (2019) proposed a plasmonic affinity sandwich assay for miR-21 using AuNP-decorated Ag@SiO_2_ core–satellite nanocomposites [167]. This platform demonstrated high sensitivity with an LOD of 10 fM and, impressively, only 5 µL of serum [167]. For metabolic characterization, Moisoiu et al. (2019) used hydroxylamine-reduced AgNPs to analyze serum metabolites extracted via methanol, achieving a sensitivity and specificity of 98% and 91%, respectively, for discriminating cancer from controls [3]. Similarly, Știufiuc et al. (2020) utilized solid plasmonic substrates (Leopold–Lendl Ag nanocolloids) purified by tangential flow filtration to detect BC from pristine plasma with 89% accuracy [9]. Other specialized methods include the HAp-based adsorption–exfoliation of serum albumin for label-free screening, which reached a diagnostic accuracy of 96.68% for BC [23]. Additionally, the analysis of low-molecular-weight fraction proteins (<30 kDa) with centrifugal filters and AgNPs identified disease markers such as prolactin and fibrinogen with 79% accuracy [168]. Finally, Nguyen et al. (2025) [169] presented a label-free platform using self-assembled AuNP clusters within microcapillaries to scan plasma metabolites. This approach, combined with a 2D-CNN machine learning model, classified BC patients with 87.5% accuracy, highlighting the potential for fast, on-site diagnostics without complex pre-treatment [169].

The studies discussed in this subsection demonstrate that SERS-based detection of circulating biomarkers is a powerful and versatile approach for breast cancer liquid biopsy. By leveraging diverse substrates and innovative probing strategies, SERS platforms can now target a wide spectrum of markers beyond cells and exosomes, reaching exceptional sensitivity and accuracy suitable for clinical translation.

#### 3.7.4. Alternative Biofluids

While blood-derived samples such as plasma and serum remain the predominant matrices for liquid biopsy, growing attention has been directed toward alternative biofluids that offer non-invasive, accessible, and patient-friendly sampling routes. Urine, saliva, tears and nipple discharge represent promising sources of tumor-derived biomarkers, each providing unique biochemical signatures that complement conventional blood-based assays. These fluids are particularly attractive because they can be collected repeatedly with minimal discomfort, thereby facilitating longitudinal monitoring and broadening the applicability of liquid biopsy in diverse clinical locations.

Urine-based SERS diagnostics: Urine is regarded as an ideal medium for real-time monitoring due to its abundance and ease of collection. Lin et al. (2020) [170] demonstrated the efficacy of a label-free SERS approach using 40 nm AuNPs combined with Principal Component Analysis–Linear Discriminant Analysis (PCA-LDA). Their method achieved a high diagnostic sensitivity of 96% for breast cancer versus healthy controls, identifying glycogen, nucleic acids, and urea as key metabolic discriminators [170]. Similarly, Moisoiu et al. (2019) utilized AgNPs activated with calcium ions to enhance the adsorption of anionic purine metabolites (uric acid, xanthine, and hypoxanthine), achieving an overall accuracy of 88% and specificity of 95% without requiring filtering or protein removal [171].

The biochemical landscape of urine was also elucidated by Iancu et al. (2022), who compared serum and urine biofluids [172]. Their findings surprisingly showed that urine-based SERS outperformed serum in classification accuracy (89% vs. 83%) by comparing three algorithms; both fluids are dominated by purine metabolite signatures; however, urine offers a more distinct metabolic profile for BC detection. To address the physiological variability inherent in urine, such as pH and dilution, Andras et al. (2025) optimized the protocol by adjusting urine to pH 9 and normalizing signals to the creatinine band at 1420 cm^−1^ [173]. This standardization significantly enhanced spectral detail and classification accuracy (85.7%), providing a robust framework for clinical translation.

Beyond label-free metabolic profiling, Kim et al. (2022) [174] developed a highly sensitive SERS platform for circulating miRNAs (miR-21 and miR-155) in urine using seed-mediated grown Ag nanopillars. This dual-targeted approach achieved zeptomole-scale LOD and successfully discriminated between healthy and breast cancer mouse models, highlighting the potential for specific molecular liquid biopsy in urine [174].

Salivary-based SERS diagnostics: Saliva offers a safe, non-invasive diagnostic medium that reflects systemic inflammation and malignancy. Hernández-Arteaga et al. (2017, 2019) focused on sialic acid as a salivary biomarker for BC [5,175]. Their research established that SA concentrations are significantly elevated in BC patients (18.3 ± 9.4 mg/dL) compared to healthy controls (3.5 ± 1.0 mg/dL). By utilizing citrate-reduced AgNPs and ROC curve analysis, they established a diagnostic threshold of 12.5 mg/dL, yielding 80% sensitivity and 93% specificity. This test can be particularly valuable for screening women with dense breast tissue, where traditional mammography often fails.

Tears and nipple discharge: Kim et al. (2020) [26] designed a well-aligned Au-decorated nanosphere monolayer SERS chip for on-site analysis of human tears. Using a portable spectrometer, they identified distinctive Raman peaks for DNA, tyrosine, and tryptophan, achieving a classification accuracy of 96% and a sensitivity of 92% for asymptomatic BC detection [26].

Furthermore, Song et al. (2024) [27] explored nipple discharge, which provides direct access to the breast ductal microenvironment where most cancers originate. They employed a magnetic-field-amplified SERS technique to detect carcinoembryonic antigen (CEA), with an antibody-conjugated silver nano-probe (AgNPs-Ab1/4-ABP) linked onto the surface of another antibody-conjugated magnetic probe [27]. This approach reached an LOD of 0.5 ng/mL and successfully distinguished between malignant lesions and benign conditions.

The evidence from these studies demonstrates that alternative biofluids provide a comprehensive molecular and metabolic landscape for breast cancer detection that often rivals or complements traditional blood-based biopsies. While challenges such as pH variability and the need for standardized protocols persist, advancements in portable instrumentation and multivariate statistical modeling (PCA-LDA, machine learning) are rapidly bridging the gap toward decentralized, point-of-care screening. These non-invasive tools hold the potential to significantly reduce mortality through earlier detection and more effective clinical monitoring.

The collective evidence across Section 3.7.1, Section 3.7.2, Section 3.7.3 and Section 3.7.4 demonstrates that SERS has become a cornerstone technology for advancing liquid biopsy in breast cancer. Exosome-based strategies (Section 3.7.1) highlight the capacity of SERS to achieve multiplexed, label-free, and highly sensitive detection of vesicular proteins and nucleic acids, enabling precise molecular subtyping and staging. CTC platforms (Section 3.7.2) address the challenges of rarity and heterogeneity through immunomagnetic capture, microfluidics, and targeted bioprobes, achieving ultra-low detection limits and reliable phenotypic profiling. Circulating biomarkers in blood (Section 3.7.3) expand the diagnostic scope to proteins, metabolites, and nucleic acids, offering systemic insights into tumor progression and therapeutic response. Finally, alternative biofluids (Section 3.7.4), including urine, saliva, tears, and nipple discharge, provide non-invasive, patient-friendly sampling routes that complement blood-based assays and broaden the applicability of liquid biopsy to decentralized and longitudinal monitoring.

Taken together, these advances underscore the versatility of SERS for capturing the molecular and cellular heterogeneity of breast cancer across diverse biological matrices. Through the integration of nanotechnology, microfluidics, bio-functionalized nanoplatforms, and advanced spectral analysis, SERS-enabled liquid biopsy approaches have evolved into increasingly robust, reproducible, and clinically relevant diagnostic tools. This convergence positions liquid biopsy not only as a minimally invasive alternative to tissue sampling, but also as a comprehensive framework for early detection, molecular characterization, and longitudinal monitoring of breast cancer. Recent studies further illustrate the evolution of SERS from conventional nanostructured sensing toward biomarker-specific and AI-assisted diagnostic approaches, including HER2 classification using ResNet-assisted analysis [176], molecular profiling with receptor-targeted nanoparticles and nucleic acid probes [177,178], and characterization of the tumor microenvironment through functional nanosensors [179]. Together, these developments highlight the increasing generation of complex spectral and molecular datasets, creating a need for advanced analytical and computational tools for data interpretation.

### 3.8. Chemometrics, Machine Learning, and AI-Assisted Diagnostic Interpretation

Chemometrics provides the mathematical and statistical foundation for extracting meaningful information from complex, high-dimensional spectroscopic data. In the context of SERS, these tools are indispensable for mitigating spectral noise, baseline drift, and the inherent complexity of biological matrices such as human serum, plasma, and urine. To overcome these limitations, computational strategies have evolved from traditional chemometrics to classic ML, and more recently, to DL and AI, each offering distinct advantages depending on data complexity [180].

Following the fourth cluster of MCA, several research papers have been included in this section. They demonstrate the successful application of previous tools across various biosensing platforms and biomarkers, as will be discussed below. A summary of the computational frameworks applied in BC SERS diagnostics is presented in Table 5, including PCA-LDA, PLS-DA, SVM, RF, KNN, MLP, CNN, ResNet, MIL-CNN, etc. This diversity illustrates the rapid methodological evolution from linear statistical models to sophisticated AI.

Traditional chemometrics remain widely used for dimensionality reduction and baseline classification. For example, Lin et al. (2021) [181] utilized hydroxyapatite microspheres to target and adsorb serum albumin, an acidic protein whose levels correlated with BC. By using a PO_4_^3─^ release reagent, they preserved the protein’s native structure, allowing PLS-DA models to analyze albumin-SERS spectra, achieving 100% sensitivity and 97.5% specificity [181]. For staging diagnostic screening, Nargis et al. (2021) demonstrated that SERS combined with PLS-DA provided superior sensitivity (90%) and specificity (98.4%) compared to conventional Raman for identifying BC stages through II-IV [66].

On the other hand, Wang et al. (2025) avoid the Vroman effect (displacement of small molecules by high-abundance proteins) through two-stage ultrafiltration, fractioning serum by molecular weight (≥30, 3–30, <3 kDa) [37]. Then, after quantitatively detecting protein components and metabolites, overlapping peaks in SERS spectra were observed. The authors further analyzed all SERS spectra with traditional PCA-LDA algorithms; however, as reported, they were unable to discriminate between benign breast tumors and breast cancer after segmentation. Then, the DNN algorithm was used, obtaining an overall accuracy of 99.53%. However, as demonstrated by Lin et al. (2019), PCA-LDA was useful in urine samples for identifying BC vs. normal, and gastric cancer vs. BC, with a diagnostic specificity of 87.5% and 90.7% [25]; a similar work was reported by Vargas-Obieta (2016) with AuNPs of 40 nm [182]; also, Lin et al. (2021) used both ML methods, highlighting the physical importance of measurement location with the “coffee-ring” area, indicating that this area concentrates more biochemical features, improving accuracy by 12.1% compared to the middle of the sample droplet, with 94.8% sensitivity and 98% specificity [57]. Wang et al. (2025) [183] used the same “coffee-ring” distribution but with AuNPs to compare LDA and SVM with Multilayer Perceptron models, with the latter presenting better accuracy of 85.7%. This demonstrated how the physical redistribution of solutes, coupled with advanced NN, can resolve complex serum profiles for clinical auxiliary diagnosis [183].

Advanced biosensor architecture has also been developed to enhance signal reliability, so Ma et al. (2020) engineered a porous Si Bragg reflector that provided a signal enhancement 3.17 times higher than single-layer PSi, enabling 95% diagnostic accuracy via PCA-LDA [56]. Hernández Cedillo et al. (2026) evaluated magnetic Ag/Ni/NiO nanowires, achieving a remarkable 99% accuracy (PCA-LDA) in differentiating major BC subtypes (LUMA, LUMB HER2, TNBC) while reaching an LOD of 10^−14^ M for rhodamine [6].

Classic ML algorithms provide stronger handling of non-linear spectral variances. Some authors use one model for classification, such as X. Lin et al. (2021), Nguyen et al. (2024), Shah et al. (2025), and Zhou et al. (2025), with DL, CNN, RF, and PLS, respectively, obtaining results with single substrates [34,57,184,185]. Indeed, S. Zhang et al. (2024) introduced a multi-modal approach combining ATR-FTIR and SERS, identifying that the integration of both techniques provided complementary molecular fingerprints of RNA biomarkers, resulting in a validation accuracy of 95.1% using only SVM [186]; other authors have found better results with two or more models.

Traditional and classic methodologies are also combined to compare parameters such as accuracy, sensitivity, and specificity. For example, Y. Lin et al. (2021) implemented a cellulose acetate membrane purification system to isolate serum proteins, where a PLS-SVM model presented higher performance than the traditional PCA-LDA model, with an accuracy of 94.67% [187]. In another study, Gao et al. (2021) utilized recursive weighted PLS (rPLS) to extract sensitive bands for OPLS-DA, LDA, AND PCA-SVM models, with the rPLS-LDA model achieving a peak accuracy of 97.77% for simultaneous cervical and BC screening [188]. Similarly, Liu et al. (2022) applied OPLS-DA to classify normal and breast cancer cells, reaching a predictive performance of 0.714 [189].

Instead, Xie et al. (2026) focused on cellular-level analysis using TiO_2_ nanotube arrays modified with AgNPs [180], achieving a 100% accurate classification of cell lines via composite ML models (PCA, LDA, DT, RF, KNN, and LR). While PCA gives a comprehensive dimensionality reduction in high-dimensional data, i.e., assesses the differences between the whole spectra of cells, LDA maximizes the separation between samples of different categories and minimizes the scatter within the same categories. Then the authors opted to create a composite model based on LDA (LDA-DT, LDA-RF, etc.). They found that the results from the LDA-DT model were relatively weak in recognizing cancer cells.

Deep learning and artificial intelligence surpass traditional methods by automatically extracting hierarchical features; some examples are described further. ResNet-based CNNs have been used to differentiate label-free exosomes from malignant (MCF-7, MDA-MB-231) and normal (MCF-10A) cell lines, leveraging metabolically active biomarkers like nucleotides and proteins to achieve 95% accuracy [190]. Similarly, Shin et al. (2023) employed a Multiple Instance Learning (MIL)-CNN to analyze plasma exosomes for pan-cancer screening, reporting an area under the curve (AUC) of 0.97 for cancer presence and effectively identifying BC with a sensitivity of 95.7% [38].

A significant innovation in SERS-AI is the conversion of 1D Raman spectra into 2D images to leverage the spatial feature-learning capabilities of 2D-CNNs. Cheng et al. (2024) utilized the Gramian Angular Field technique to generate 2D spectral images, achieving an accuracy of 98.13%, a sensitivity of 98.65%, and an AUC of 0.9884 [191]. Their results indicate that 2D-CNNs capture richer contextual information than 1D-CNNs or traditional SVM models. Lin et al. (2025) also compared heatmap and Continuous Wavelet Transform (CWT) transformations, finding that heatmap-based ResNet18 models yielded superior accuracy (94.75%) for multi-cancer screening [192].

Other authors use hybrid models combining feature selection with DNNs, reaching exceptional precision for complex tasks like molecular subtyping; Zhang et al. (2024) developed a model integrating LightGBM (LGB) for feature selection with a DNN [193]. LGB identified critical wavenumbers contributing to classification, enabling the model to distinguish BC from benign BC and healthy controls with 96.40% accuracy. Crucially, this method achieved high accuracy in evaluating molecular subtypes, such as HR+/HR− (90.11%) and HER2+/HER2− (88.89%).

Throughout this section, we have reviewed how SERS-AI platforms demonstrate substantial analytical potential. Nevertheless, their clinical translation will depend less on nominal classification accuracy and more on patient-level data partitioning, standardized reporting, multicenter external validation, and prospective testing.

Overall, Table 5 reveals that the evolution of SERS-AI systems is driven not only by increasingly sophisticated algorithms, but also by substantial advances in nanoarchitecture design. Colloidal Ag NPs have progressively evolved toward ordered hotspot-bearing substrates, core–shell nanoscale tags, dual-targeted probe systems, microfluidic platforms, plasmonic superclusters, and multiplexed immunoassays. These architectures govern signal amplification, reproducibility, molecular selectivity, and data dimensionality, thereby determining the quality of information available to ML models. Notably, high diagnostic performance cannot be attributed to the algorithm alone, but rather to the integrated sequence nanoarchitecture design, hotspot and probe organization, biomolecular recognition, SERS signal quality, and, ultimately, AI-assisted prediction.

Building on this analysis, Table 5 also provides a more critical framework for evaluating the clinical translation of SERS-based breast cancer diagnostic methods. From this framework, the most significant and still unresolved gap emerges as the absence of a systematic co-design framework linking nanoarchitecture properties to AI model robustness and clinical generalizability. Accordingly, future studies should move beyond the independent optimization of SERS substrates and classification algorithms to determine how hotspot distribution, probe organization, surface bio-functionalization, batch-to-batch variability, and signal stability affect model predictions across different instruments, laboratories, and patient populations.

**Table 5:** Computational and AI algorithms applied in SERS-based breast cancer diagnostics.

Reference	Dataset and Classes	Classification/Analytical Task	Validation Strategy	SERS Substrate/Platform	Accuracy (%) Sensitivity (%) Specificity (%)	Other Performance and Within-Study Comparison	Application	Evidence Limitation
Chemometric classifier: PCA-LDA ^a^; PLS-LDA ^b^ and PLS-DA; PCA ^c^								
[6] ^a^	31 FFPE tissue specimens/patients: normal 6; Luminal A 8; Luminal B 5; HER2-positive 4; TNBC 8. Nine spectra/patient; 837 spectra acquired; analyses used 31 patient-level averaged spectra.	Cancer vs. normal; luminal vs. non-luminal; HER2 vs. non-HER2; TNBC vs. non-TNBC; five-class subtype classification.	Repeated stratified k-fold cross-validation at patient level; k NR in main article.	Magnetically aligned Ag/Ni/NiO nanowires on Al.	99 (reported) 99 99	All samples are stated to be correctly classified, which mathematically implies 100%, although the article reports 99%.	Tissue diagnosis and molecular subtyping.	Small retrospective pilot cohort; no external validation.
[56] ^a^	60 serum samples: 30 early breast cancer and 30 healthy controls; three spectra/sample.	Binary early breast cancer vs. healthy classification.	Leave-one-out cross-validation.	AgNP-decorated porous-silicon Bragg reflector.	95.0 93.3 96.7	Eight PCs gave the best LDA accuracy. Two controls and one cancer case were misclassified.	Early breast cancer serum screening.	Small single-center cohort; internal LOOCV only; no independent or external validation.
[25] ^a^	141 urine samples/spectra: healthy 48; gastric cancer 50; breast cancer 43.	Three-class discrimination; reported metrics are one-vs-rest for breast cancer.	Leave-one-sample-out cross-validation.	Phenylboronic-acid affinity purification of urinary modified nucleosides + colloidal AuNPs.	79.4 62.8 86.7 for BC	AUC for breast cancer discrimination = 0.914. Gastric-cancer accuracy = 85.8%; healthy-class accuracy = 79.4%.	Non-invasive urine-based multicancer screening.	Single-center exploratory cohort; moderate breast cancer sensitivity; no independent or external test cohort.
[182] ^a^	27 donors: 12 advanced breast cancer patients and 15 controls; 289 spectra (139 cancer, 150 control). One cancer donor contributed an additional sample.	Binary breast cancer vs. healthy classification.	Leave-one-spectrum-out/random spectral cross-validation; patient grouping not reported.	40 nm colloidal AuNPs mixed with serum.	91.3 (calculated based on published data) 96.0 87.0	Accuracy calculated from the reported confusion counts: (133 + 131)/289. Six cancer and 19 control spectra were misclassified.	Serum-based detection of advanced breast cancer.	Metrics are spectrum-level, not patient-level; high pseudoreplication/data-leakage risk; very small and advanced-stage-only cohort.
[194] ^a^	Three cell types: HER2+ SK-BR-3, triple-negative MDA-MB-231 and WBCs; 50 cells/SERS spectra per class.	Three-class discrimination and breast cancer molecular subtyping.	7:3 train/test split with five-fold cross-validation; grouping beyond individual spectra not applicable/reported.	Dual HER2-targeted AuNP@4-MBA@PDA and AuNP@4-MPY@PDA SERS bioprobes (‘symphonic’ spectra).	97.33 NR NR	Single-probe five-fold CV accuracies: 86.24% and 84.46%; combined probes: 97.33%. Test-set class AUCs were 1.00; the paper also states all test predictions were correct.	Detection and HER2-based molecular typing of breast cancer cells.	Small in vitro cell-line study; no patient specimens or external validation.
[181] ^b^	70 serum donors: 30 breast cancer patients and 40 healthy volunteers; albumin isolated before SERS.	Binary breast cancer vs. healthy classification.	Leave-one-out cross-validation.	Hydroxyapatite microsphere albumin adsorption/release + AgNP SERS.	98.6 100 97.5	Three latent variables selected; one healthy sample was misclassified.	Targeted serum-albumin breast cancer screening.	Single-center cohort; internal LOOCV only; no independent or external validation.
[66] ^c^	29 serum donors: 12 healthy and 17 breast cancer (stage II-7; stage III-5; stage IV- 5); 15 spectra/sample for both Raman and SERS.	Healthy vs. breast cancer classification; stages explored.	Cross-validated training model applied to the remaining dataset; split size and patient-level grouping NR.	Colloidal AgNPs; SERS compared with conventional Raman.	NR 90.0 98.4	SERS: AUROC 0.940 vs. Raman: sensitivity 88.2%, specificity 97.7%, AUROC 0.834.	Serum detection and stage-related spectral assessment.	Very small cohort; no stage-I cases; unclear train/test composition and whether repeated spectra were grouped by patient.
Deep learning: DNN spectral unmixing/quantification ^d^; CNN ^e^; ResNet-based CNN/PCA ^f^; MIL-CNN/SVM baseline ^g^; ResNet on Heatmaps/DNN on 1D spectra/ BorderlineSMOTE ^h^								
[57] ^d^	Training: 1000 spectral points from each of four pure SERS tags plus 500 from a 1:1:1:1 mixture; test: 50 points/tag spectrum. Biological testing: five cell-line exosome types and urine pooled from 28 mice in two groups.	Quantification of multiplexed exosomal HER2/GRB7/GAPDH signals and trastuzumab-response monitoring; not a diagnostic classifier.	Biological agreement checked by qRT-PCR and Western blot; no diagnostic test set.	Au@Ag embedded-reporter SERS tags + exosome sandwich immunoassay.	NA NA NA	Reported expression changes, not classification metrics; e.g., HER2/GAPDH decreased after treatment in both mouse groups.	Preclinical therapeutic-efficacy monitoring.	Mouse and cell-line study; pooled urine; no human clinical validation; classification metrics are not applicable.
[184] ^e^	148 plasma donors: 74 breast cancer and 74 healthy controls; three pillars/donor = 444 spectral samples, each with 1600 features.	Binary breast cancer vs. healthy classification.	80:20 train: test split plus five-fold cross-validation repeated 20 times; donor-level grouping of the three pillars not reported.	3D-printed AuNP plasmonic superclusters from hybrid bioink.	93.37 ± 2.51 (CV) 91.9 94.6	AUC = 0.932; training accuracy reached 99%. The 91.9% and 94.6% values are class-wise test accuracies corresponding to sensitivity and specificity.	Plasma-metabolite breast cancer detection.	Single-center cohort; no external validation
[190] ^f^	1160 spectra from cell-derived exosomes: MCF-10A 400, MDA-MB-231 380 and MCF-7 380.	Three-class classification of exosome cellular origin.	70% training, 10% validation, 20% test; biological replicate grouping NR.	Label-free exosome/AuNP dried mixture SERS.	>95 NR NR PCA showed overlapping clusters for sensitivity/specificity: 81.2/56.3% 89.7/80.8% 67.0/66.1%	CNN AUC: MDA-MB-231 0.999, MCF-7 0.991, MCF-10A 0.997.	Breast-cell-derived exosome identification.	Cell-line-only data; spectra rather than independent subjects; CNN sensitivity/specificity not reported.
[38] ^g^	753 plasma samples: 210 healthy controls and 543 cancers (six organs). Training *n* = 233 (50 healthy, 183 cancer); independent test *n* = 520 (160 healthy, 360 cancer), including 70 breast cancer cases. One hundred spectra/sample.	Cancer presence and TOO; breast cancer-specific metrics reported within the multicancer test set.	Independent held-out test set.	Label-free plasma-exosome SERS on AuNP colloidal dot array in PDMS wells.	94.8 95.7 94.4	Breast cancer presence: AUC 0.984. Breast TOO vs. other cancers: AUC 0.876, sensitivity 71.4%, specificity 93.1%. CNN outperformed SVM and dummy baselines.	Early multicancer detection and localization, including breast cancer.	Retrospective biobank study; TOO sensitivity for breast cancer was lower than cancer-presence sensitivity.
[192] ^h^	3551 serum donors: 1655 early cancers (BC 569; lung 513; thyroid 220; colorectal 215; gastric 100; esophageal 38) and 1896 healthy controls; one averaged spectrum/participant.	Seven-class early multicancer detection, including breast cancer.	Participant-level random split with a 7:3 train: test ratio; resampling applied to the training data; no external cohort.	Label-free serum SERS with ~40 nm colloidal AgNPs; 1D spectra transformed into heatmaps/CWT images.	94.75 86.97 (macro recall) NR	ResNet heatmap: precision 89.02%, F1 87.88%; class AUCs 0.940-0.999 (BC 0.995). DNN with BorderlineSMOTE: accuracy 93.15%, recall 85.68%, F1 86.98%.	Large-scale serum-based early multicancer screening.	Case–control design and single random split; specificity was not reported; recall is macro-averaged rather than breast cancer sensitivity; no prospective/external validation.
Machine learning: KNN for SERS/SVM for ATR-FTIR and multimodal data ^i^; Random Forest/t-SNE for visualization ^j^; LDA-DT, LDA-RF, LDA-KNN, LDA-logistic regression ^k^								
[186] ^i^	91 clinical serum-miRNA samples: 44 malignant and 47 benign; 20 measurements/sample per modality, averaged for analysis.	Binary malignant vs. benign breast lesion classification.	Six blind test-size scenarios (5–30 samples), each repeated three times; ten-fold cross-validation within training/validation data.	Ag-coated Si nanopillars for SERS; combined with ATR-FTIR in the multimodal model.	95.1 9.15 ~95 (multimodal validation)	Best models: KNN for SERS alone; SVM for ATR-FTIR and multimodal. Best multimodal AUC = 0.9979 for 86/5 split; AUC = 0.8571 for 61/30 split.	Label-free early breast cancer RNA fingerprinting.	The 95.1% result is multimodal, not SERS alone; no external cohort.
[34] ^j^	Three breast cancer cell lines (MDA-MB-231, MCF-7, SKBR-3). Pure-cell integrin profiles used for training; 450 mixed cells in PBMC matrix used for prediction.	Three-class cell-line/subtype classification from five-integrin profiles.	70:30 train/test split; 200 repeated training iterations; then retrained for mixed-cell prediction.	Five-color gap-enhanced Au nanorods (GENRs) with integrin antibodies; PBMC spike-in model.	99.9 (claimed) NR NR	Mean F1 = 0.99; predicted mixed cells: 148 MCF-7, 22 SKBR-3 and 280 MDA-MB-231.	Single-cell integrin profiling and putative CTC subtype classification.	In vitro/spike-in study without clinical patient samples; accuracy claim is not supported by a separately reported accuracy calculation.
[180] ^k^	Three cell lines: MDA-MB-231, MCF-7 and MCF-10A; number of spectra per class NR.	Three-class breast-cell-line recognition.	Stratified 80:20 train/test split; grouping by biological replicate NR.	TiO_2_ nanotube arrays decorated with AgNPs.	100 (reported) NR NR	AUCs for LDA-DT by class: 0.97, 0.94 and 1.00; LDA-RF, LDA-KNN and LDA-logistic regression: AUC 1.00 for all three classes.	Rapid recognition of malignant and normal breast cell lines.	In vitro cell-line data; dataset size and independent biological replicates NR.
[42] ^j^	Three breast cancer cell lines (SKBR-3, MM-231, MCF-7); individual-cell marker profiles used for training	Three-class cell-line classification from CD44/HER2/EpCAM profiles.	70:30 train/test split; 200 repeated trainings; whole training set then used for mixed-cell prediction.	Gap-enhanced magnetic-plasmonic IO/Au/gap/Au Raman nanotags.	93.9 NR NR	Mean F1 = 0.94; predicted-vs-test Pearson r = 0.94;	Multiplexed surface-protein profiling and putative CTC classification.	Cell-line/spike-in model; no patient-derived CTC validation; sensitivity and specificity were not reported.
Machine learning/chemometrics: PLS-SVM vs. PCA-LDA ^l^; rPLS-OPLS-DA, rPLS-LDA and rPLS-PCA-SVM ^m^; SVM/PLS-DA comparators ^n^;LDA, SVM and MLP, neural network ^o^								
[187] ^l^	75 serum donors: 30 breast cancer and 45 healthy controls; ten spectra/sample were averaged to represent each donor.	Binary breast cancer vs. healthy classification.	Ten-fold cross-validation.	Cellulose-acetate membrane protein purification + colloidal AgNP SERS.	94.67 90.00 97.78	PLS-SVM: accuracy 94.67%, sensitivity 90.00%, specificity 97.78%, AUC 0.940; PCA-LDA: 84.00%, 86.67%, 82.22%, AUC 0.856.	Label-free serum-protein breast cancer screening.	Single-center cohort; internal cross-validation only; no independent or external validation.
[188] ^m^	335 serum donors: healthy 146, cervical cancer 100, breast cancer 89. Training *n* = 201; held-out test *n* = 134. Ten spectra/sample averaged.	Three-class simultaneous screening; breast cancer values are one-vs-rest.	Held-out test set; rPLS-OPLS-DA also assessed by 50 permutations and LOOCV; SVM parameters tuned by cross-validation grid search.	AuNPs on 785 nm porous-silicon photonic crystals.	97.77 (best overall) 100 (BC, all models) 97.8 (BC, best rPLS-LDA)	Overall accuracy: rPLS-LDA 97.77% vs. rPLS-PCA-SVM 94.78% vs. rPLS-OPLS-DA 93.28%. Breast AUC: 0.997, 0.956 and 0.948, respectively.	Simultaneous non-invasive screening of breast and cervical cancer.	Single-center data; no external cohort
[172] ^n^	FNA specimens from 78 patients with breast cancer, fibroadenoma or breast hyperplasia; 2–10 spectra/sample. Three-class SVM validation counts total 61 spectra; binary SVM used 226 spectra (BC 175; hyperplasia 51).	Three-class breast-disease discrimination; binary breast cancer vs. hyperplasia.	Training 68–76% and test 24–32%; SVM verified with 10-fold validation and PCA-LDA with LOOCV; patient-level grouping NR.	FNA sample SERS with 50–60 nm colloidal AgNPs and a portable Raman spectrometer.	90.16 (3-class SVM)/94.74 (BC)/86.96 (BC)	Three-class SVM sensitivity/specificity: BC 94.74/86.96%, fibroadenoma 83.33/100%, hyperplasia 81.82/94.00%. PLS-DA accuracy 71.88%; binary SVM accuracy 94.2% vs. PLS-DA 89.86% and PCA-LDA 84%.	Minimally invasive early differential diagnosis of breast lesions from FNA.	Only 78 patients, but multiple spectra/sample; single-center cohort and no external validation.
[183] ^o^	192 breast cancer serum donors: Luminal A 47, Luminal B 45, HER2-positive 56, TNBC 44; 220 constructed spectral lines/class after balancing. Healthy samples (*n* = 50) were characterized but not included in the four-subtype classifier.	Four-class molecular-subtype classification.	Five-fold cross-validation; whether folds were grouped by patient NR.	AuNP serum coffee-ring with combined inner/outer radial spectra.	85.7 NR NR	Combined-ring data: MLP 85.7% vs. SVM 68.3% vs. LDA 58.2%. Outer-ring only: MLP 70.3%, SVM 58.6%, LDA 49.9%. MLP AUC range 0.933–0.991 for combined data.	Serum molecular-subtype classification.	No independent/external validation; undersampling and constructed spectral lines
Chemometric regression: PLS regression ^p^; chemometric modeling/no reported classification rate: PCA and OPLS-DA ^q^								
[185] ^p^	Four cell lines (MCF-7, MDA-MB-231, LM2 and BrM2) exposed to known Zn^2+^ concentrations (10^−4^ to 10^−12^ M); number of spectra/biological replicates NR.	Intracellular Zn^2+^ quantification across breast cancer metastatic phenotypes; not diagnostic classification.	Ten-fold cross-validation for latent-variable selection.	Au@SiO_2_ hollow nanocapsules with terpyridine Zn^2+^ probe.	NA NA NA	R2 calibration > 0.98 and R2 cross-validation > 0.99; RMSEC/RMSECV reported by cell line; 5–9 latent variables.	Mechanistic quantification of zinc homeostasis and metastasis.	Cell-line mechanistic study; no diagnostic classes or clinical samples; accuracy/sensitivity/specificity are not applicable.
[189] ^q^	Cell lysates from MCF-10A, MCF-7 and MDA-MB-231; 60 SERS spectra/cell line.	Normal vs. cancer and MCF-7 vs. MDA-MB-231 cell-line separation.	Random-permutation testing; no held-out diagnostic test set.	Self-assembled Ag nanorods.	NR NR NR	Normal vs. cancer OPLS-DA: R^2^Y 0.833, Q^2^ 0.714. MCF-7 vs. MDA-MB-231: R^2^Y 0.715, Q^2^ 0.646. No classification accuracy was reported.	Label-free cell-lysate differentiation.	In vitro spectra; no clinical samples; no held-out test set; the LOD of 5.3 µM refers to creatinine, not breast cancer classification.
Deep learning/machine learning: 2D-CNN-GAF vs. 1D-CNN, SVM, KNN and PCA-LDA ^r^; LightGBM feature selection + DNN/comparators: KNN, SVM, RF, AdaBoost, 1D-CNN and ANN (hybrid ML/deep learning) ^s^; K-Nearest Neighbors, KNNs/machine learning ^t^								
[191] ^r^	128 serum donors: 69 breast cancer and 59 healthy; 3200 GAF images (1725 cancer, 1475 healthy) generated from spectra.	Binary breast cancer vs. healthy classification.	90% for five-fold cross-validation/model development; independent 10% image validation (320 images).	Colloidal AgNP SERS; Gramian angular field converts 1D spectra into 2D images.	98.13 (independent images) 98.65 97.67	CV accuracy: 2D-CNN 98.44%, 1D-CNN 94.21%, SVM 93.84%, PCA-LDA 92.87%, KNN 90.43% (best preprocessing). AUC: 0.9884, 0.9704, 0.9613, 0.9544 and 0.9648, respectively.	Automated serum breast cancer screening.	Image-level rather than donor-level split is not clarified; no external cohort.
[193] ^s^	833 serum donors/spectra: BC 207, benign breast disease 83 and healthy controls 543. BC subtyping: HR− 112/HR+ 70; HER2− 142/HER2+ 65.	BC vs. benign disease; BC vs. benign vs. healthy; HR and HER2 molecular-subtype classification.	Internal training/validation monitoring and model tuning; no external cohort.	Label-free serum SERS with AgNPs; one averaged spectrum/participant.	98.30 (macro) 92.76 (macro recall) 98.30 (macro)	LGB-DNN: BC vs. benign 91.38/89.27/85.71%; HR 90.11/90.09/90.18%; HER2 88.89/85.65/85.64% (accuracy/recall/specificity).	Non-invasive breast cancer screening and serum molecular subtyping.	Single-center cohort; no independent/external validation.
[195] ^t^	Three breast cell lines: normal MCF-10A, mildly malignant MCF-7 and malignant MDA-MB-231. Training: 270 spectra (90/class); testing: 180 spectra per independent experiment (60/class), repeated in three experiments.	Three-class label-free breast-cell-line identification.	Separate spectral test sets in three independent experiments.	Dynamic liquid SERS in soft tubular microfluidics with positively modified silver nanostars.	94.1 ± 1.14 >83.3 (each class) >91.6 (each class)	One displayed experiment had 94.4% overall accuracy; platform RSD was 1.90% within-run and 4.98% across nine experiments.	Rapid label-free identification of breast cell phenotypes.	In vitro pure cell-line populations; spectra reflect multiple cells in the laser focus; no patient samples and no external validation.

Notes: Accuracy, sensitivity and specificity are reported for the validation or test setting specified in each article. Values from training sets were not used as the principal performance estimate. NR: not reported; NA: not applicable because the study performed regression, quantification or mechanistic analysis rather than diagnostic classification; AUC/AUROC: area under the receiver operating characteristic curve; CNN: Convolutional Neural Network; RF: Random Forest; ResNet: Residual Network; SVM: Support Vector Machine; KNNs: K-Nearest Neighbors; DL: deep learning; DNN: Deep Neural Network; MLP: Multilayer Perceptron; PLS: Partial Least Squares; DNN-LightGBM: Deep Neural Network–Light Gradient Boosting Machine; MIL-CNN: Multiple Instance Learning Convolutional Neural Network; OPLS-DA: Orthogonal Partial Least-Squares Discriminant Analysis; 2D-CNN: 2-Dimensional Convolutional Neural Network; GAF: Gramian Angular Field; PCA: Principal Component Analysis; LDA: Lineal Discriminant Analysis; TOO: tissue of origin. Matching superscript letters in the first column denote the association between each author (or study) and the methodology applied in the corresponding work. Cross-study performance values are not directly interchangeable because the studies differ in biological unit, sample type, class definition, preprocessing, data augmentation, validation design and whether splitting was performed at the patient, sample, spectrum or derived-image level. Algorithmic comparisons are strongest when made within the same study and dataset.

Addressing this gap will require standardized nanoarchitecture descriptors, patient-level data partitioning, multicenter external validation, the use of prospective cohorts, and direct algorithm comparisons performed on identical biological samples and SERS platforms. Ultimately, systems with genuine clinical translation potential must demonstrate not only high classification accuracy, but also reproducible nanofabrication, interpretable structure-signal-prediction relationships, calibration transferability, and robust performance under real-world conditions.

### 3.9. Translation Achievements: Preclinical and Clinical Studies

This section continues the MCA framework, maintaining the transition trajectory of SERS biosensors in BC diagnostics and therapy from advanced proof-of-concept experiments to preclinical validation and early clinical implementation. Here, SERS technologies are tested in biologically relevant models, patient-derived samples, and clinical workflows, demonstrating their proximity to moving from laboratory innovation into precision oncology applications.

#### 3.9.1. Preclinical Models

Preclinical studies have increasingly focused on multimodal nanoprobes that combine SERS with imaging modalities such as confocal fluorescence microscopy, MRI and photoacoustic (PA) imaging [30]. These hybrid systems overcome traditional Raman limitations in penetration depth and spatial resolution. For example, a 3D-printed PEGDA-based scaffold was presented, supporting both SERS bioimaging and label-free biosensing to track tumor microenvironment (TME) dynamics over time [196].

Advances in spatially offset Raman spectroscopy (SESORRS) have extended detection depths to 15–25 mm of tissue, validated in multicellular tumor spheroids with a handheld SORS spectrometer [197]. Dual-modal systems, such as gold nanorods-iron oxide bioprobes, combine SERS sensitivity with MRI deep-tissue tracking for PD-L1 monitoring in TNBC [198]. Another dual-modal system was proposed by Wang et al. (2016) [199], reporting a dual-mode system based on immunofluorescence (IF) and immuno-SERS (iSERS) microscopy, applied to formalin-fixed paraffin-embedded (FFPE) tissue sections, the clinical gold standard, to localize HER2 expression. In this system, wide-field IF facilitates rapid global analysis, while iSERS provides high-resolution local mapping of cell membranes using gold nanostars [199]. Also, advancement in this domain involves the “MATRA” probe, which expands the multimodal repertoire to include T1-weighted MRI and NIR-II photothermal immunotherapy. Preclinical studies in 4T1 breast tumor models demonstrated that the integration of MRI/SERS surgical navigation with postsurgical anti-PD-1 antibody delivery effectively suppressed local recurrence and eradicated distant lung and liver metastases by triggering a systemic immune response [200].

Triple-modality nanoprobes (TMNPs) have also been developed to cover the entire perioperative window, integrating optoacoustic (preoperative), fluorescence (intraoperative), and SERS (clean margin verification) capabilities. These probes utilize DNA functionalization as a “nanoscopic ruler” to optimize the distance between near-infrared fluorophores and the gold nanorod surface, balancing SERS enhancement and fluorescence quenching. These TMNPs effectively target FOLR1-overexpressing TNBC in patient-derived xenograft models and facilitate image-guided PTT [201]. Another example is the proposal by Wen et al. (2021) [202], which is not limited to simple detection, integration of theranostic nanoprobes that combine preoperative localization with intraoperative eradication of residual microtumors. The “starPART” probe, utilizing a gold nanostar core, integrates PA imaging, SERS detection, and PTT. In orthotopic breast cancer models, this system allowed for preoperative tumor demarcation via PA imaging and the subsequent SERS-guided “thermosurgery”, which eradicated submillimeter residual foci post-resection, achieving a 100% tumor-free survival rate [202].

Preclinical innovations also address metastasis research, with microfluidic chips enabling real-time detection of interleukins (IL-6 and IL-8) during extravasation [29], demonstrating how SERS can replicate tumor complexity, monitor therapeutic responses, and overcome technical barriers such as penetration depth and imaging speed.

#### 3.9.2. Clinical Translation Studies

Clinical translation emphasizes intraoperative tumor margin delineation and multiplexed biomarker profiling. Davis et al. (2018) validated CD47 antibody-labeled SERS core–shell nanoparticles (Au@Si) in xenografts and an ex vivo human carcinoma specimen [14], achieving strong tumor-to-normal tissue discrimination, as well as theranostic applications that combine SERS imaging with PTT. Feng et al. (2017) demonstrated Au NBPs conjugated with 2-naphthalenethiol (2-NAP) and folic acid for targeted MCF-7 cell imaging and tumor reduction [63], while Wang et al. (2020) introduced bioorthogonal nanotags in the Raman-silent region (2205 cm^−1^) with a diynl group, achieving high-contrast in vivo imaging and 99% tumor inhibition [203].

Furthermore, Zhang et al. (2025) [204] designed a SERS-bioluminescence dual-modal imaging strategy using macrophage-membrane-encapsulated probes. This system uses bioorthogonal click chemistry to track TNBC metastasis to critical organs like the lungs and liver in real-time, providing a highly sensitive method for assessing therapeutic responses to drugs like adriamycin and ifosfamide [204]. Ou et al. (20218) also achieved longitudinal tracking of PD-L1 and EGFR biomarkers in vivo using gold nanostars, showing that maximum accumulation occurred 6 h post-intravenous injection and correlating SERS spatial maps with vascular density ex vivo [205].

Clinical pathology has benefited from spectro-histology platforms. Murali et al. (2023) achieved triplex detection of ER, PR, and HER2 in formalin-fixed paraffin-embedded tissues, aligning perfectly with traditional immunohistochemistry and fluorescent in situ hybridization grading [141]. J. Li (2023) presented a volume-active nanoprobe, which has 32 resolvable colors in the Raman-silent region using bioorthogonal Raman molecules [206], enabling multiplexed imaging of immune checkpoint networks in biopsies. These advances demonstrated SERS as a powerful complement to conventional pathology, offering speed, objectivity, and multiplexing capacity.

The translational achievements of SERS biosensors in breast cancer reflect a continuum of innovation that mirrors the MCA trajectory. Beginning with preclinical models, these systems replicate tumor complexity and validate multimodal nanoprobes for imaging and therapy, demonstrating tumor sensitivity-stroma interactions, metastatic processes, and therapeutic responses. Progressing into clinical feasibility studies, SERS has confirmed its ability to detect and grade biomarkers in patient tissues and biofluids, establishing itself as a reliable adjunct to conventional pathology.

The next stage, intraoperative and theranostic applications, highlights the transformative potential of SERS in surgical oncology, enabling real-time tumor margin delineation, margin assessment, and integrated imaging therapy strategies that combine detection with photothermal eradication of residual disease. Finally, clinical translation studies validate SERS in patient cohorts, achieving multiplexed profiling, therapy monitoring, and immunotherapy response prediction, thereby underscoring its inclination for integration into precision oncology workflows.

## 4. Conclusions and Future Perspectives

The rapid evolution of SERS over the past decade has positioned it as a promising analytical tool in the landscape of breast cancer diagnostics. As evidenced by our comprehensive bibliometric analysis (2016–2025), the field has experienced exponential growth in global scientific production, driven by interdisciplinary collaborations across nanotechnology, physics, analytical chemistry, and oncology. Bibliographic mapping and Multiple Correspondence Analysis highlight a transition from fundamental substrate engineering to complex applications such as biomedical diagnostics.

The available evidence indicates that the clinical potential of SERS in breast cancer depends on the interaction among nanoarchitecture, biofunctionalization, and computational analysis, rather than on the isolated sophistication of any single component. Although advanced nanostructures such as plasmonic nanopillars, multiplexed nanotags, and exosome biosensors achieve high analytical sensitivity, they do not always exhibit the highest degree of clinical validation. In parallel, platforms biofunctionalized with antibodies, aptamers, or DNA/LNA probes increase molecular specificity, whereas label-free approaches preserve a broader range of biochemical information, although the cost of requiring more complex algorithms for interpretation.

The observed evolution from conventional chemometric methods (PCA-LDA) toward machine learning models (SVM, Random Forest, XGBoost) and deep learning architectures (CNN, Transformer) reflects this transition. The principal challenge remains the reproducible integration of nanoarchitecture, biofunctionalization, and artificial intelligence. Future studies should therefore prioritize standardization, multicenter validation, and model interpretability to facilitate the clinical translation of SERS-AI platforms.

## Data Availability

Available on request from the corresponding authors of the data presented in this review.

## Supplementary Materials

The following supporting information can be downloaded at https://www.mdpi.com/article/10.3390/bios16090511/s1, Table S1: comparison of previous reviews and the present review on SERS-based breast cancer detection. Table S2: original Scopus dataset, Table S3: original Google Scholar dataset, Table S4: original Web of Science dataset, Table S5: binary matrix for MCA construction, Table S6: standardization dictionary for MCA, Table S7: curated keyword list, Table S8: RStudio code for MCA. (References [44,45,46,47,48,49,50,51,207,208,209,210,211,212,213,214] are cited in the Supplementary File).

## Abbreviations

The following abbreviations are used in this manuscript:

SERS	Surface-Enhanced Raman Spectroscopy
BC	Breast Cancer
MCA	Multiple Correspondence Analysis
ML	Machine Learning
AI	Artificial Intelligence
MRI	Magnetic Resonance Imaging
ER	Estrogen Receptor
PR	Progesterone Receptor
AuNPs	Gold Nanoparticles
AgNPs	Silver Nanoparticles
EM	Electromagnetic Mechanism
LSPR	Localized Surface Plasmon Resonance
CM	Chemical Mechanism
EF	Enhancement Factor
HCPCs	Hierarchical Clustering on Principal Components
LOD	Limit of Detection
miRNAs	Micrornas
WoS	Web of Science
GS	Google Scholar
JIFs	Journal Impact Factors
HOMO	Highest Occupied Molecular Orbital
LUMO	Lowest Unoccupied Molecular Orbital
pM	Picomolar
NCs	Nanocubes
GO	Graphene Oxide
TNBC	Triple-Negative Breast Cancer
AuNs	Au Nanostars
RBTIC	Rhodamine B Isothiocyanate
MGTI	Malachite Green Isothiocyanate
DTDC	3,30-Diethylthiadicarbocyanine Iodide
4MBA	Mercaptobenzoic Acid
R6G	Rhodamine 6G
EpCAM	Anti-Epithelial Cell Adhesion Molecule
ErbB2	Anti-Erythroblastic Oncogene B2
CD44	Anti-Cluster of Differentiation
EGFR	Epidermal Growth Factor Receptor
CTCs	Circulating Tumor Cells
PD-L1	Programmed Death-ligand 1
sEVs	Small Extracellular Vesicles
4-MBA	4-Mercaptobenzoic Acid
4-mp	4-Mercaptopyridine
CV	Crystal Violet
MB	Methylene Blue
FA	Fibroadenoma
FCC	Fibrocystic change
GaN	Gallium Nitride
FAM	Fluorescent Molecule
TNB	5-Thio2-Nitrobenzoic Acid
SAMs	Self-Assembled Monolayers
EV	Extracellular Vesicles
PG	Protein G
PTT	Photothermal Therapy
PDT	Photodynamic Therapy
PB	Prussian-Blue
SELEX	Systematic Evolution of Ligands by Exponential Enrichment
4-Mpy	4-Mercaptopyridine
PBA	Prussian Blue Analogue
RCA	Rolling Circle Amplification
DSN	Duplex-Specific Nuclease
CSC	Cancer Stem Cell
PLS-DA	Partial Least-Squares Discriminant Analysis
PCA-LDA	Principal Component Analysis–Linear Discriminant Analysis
DL	Deep Learning
cfDNA	Cell-Free DNA
CEA	Carcinoembryonic Antigen
CNN	Convolutional Neural Network
RF	Random Forest
ResNet	Residual Network
SVM	Support Vector Machine
KNN	K-Nearest Neighbors
DNN	Deep Neural Network
MLP	Multilayer Perceptron
PLS	Partial Least Squares
DNN-LightGBM	Deep Neural Network–Light Gradient Boosting Machine
MIL-CNN	Multiple Instance Learning Convolutional Neural Network
OPLS-DA	Orthogonal Partial Least-Squares Discriminant Analysis
2D-CNN	2-Dimensional Convolutional Neural Network
GAF	Gramian Angular Field
TMNPs	Triple-Modality Nanoprobes
