# Supplementary material for: Surface-Enhanced Raman Spectroscopy in Breast Cancer Detection: A Bibliometric Review and Landscape of Global Trends
Source: Biosensors (Basel). 2026 Sep 10;16(9):511. doi: 10.3390/bios16090511 (PMC13604078; doi:10.3390/bios16090511)
Supplement: Supplementary file 1 [file biosensors-16-00511-s001.zip › biosensors-4474268-Table S1.pdf]

biosensors

MDPI

Review

Surface-Enhanced Raman Spectroscopy in Breast Cancer Detection: A Bibliometric Review and Landscape of Global Trends

Alitzel B. García-Hernández[1,2,*], Gethzemani M. Estrada-Villegas[1], Ana L. Gómez-Gómez[3], Ma. de la Paz Salgado-Cruz[3,*] and Dana M. Cortez Landa[3]

[1] Centro de Investigación en Química Aplicada (CIQA), Parque de Investigación e Innovación Tecnológica, Apodaca 66628, Nuevo León, Mexico
[2] Departamento de Ingeniería Mecatrónica, Universidad Politécnica de Apodaca, El Barrenal, Apodaca 66600, Nuevo León, Mexico
[3] Departamento dde Ingeniería Bioquímica, Escuela Nacional de Ciencias Biológicas, Instituto Politécnico Nacional, U. P. Adolfo López Mateos, Ciudad de México 07738, Mexico
* Correspondence: ali_ialee@outlook.com (A.B.G.-H.); msalgadoc@ipn.mx (M.d.l.P.S.-C.)

Methods

Classification of Studies and Selection of Methodological Frameworks

Due to the marked methodological heterogeneity of the included literature—ranging from physicochemical development of SERS substrates and in vitro analytical validation studies to in vivo preclinical investigations, clinical diagnostic accuracy studies, and predictive models based on AI—a structured evaluation strategy was adopted at three complementary levels: (*i*) completeness and transparency of reporting, (*ii*) risk of bias and applicability, and (*iii*) analytical, technical, and translational validation.

These levels were assessed independently. Consequently, reporting quality was not interpreted as evidence of methodological validity, analytical reproducibility, or clinical applicability. Likewise, high analytical sensitivity, diagnostic accuracy, or predictive performance was not considered sufficient evidence of clinical utility.

1. Assessment of Reporting Completeness

Reporting completeness was evaluated using international guidelines specific to each study design, all indexed by the EQUATOR Network (enhancing the quality and transparency of health research) [1].

- Diagnostic accuracy studies assisted by AI were examined using STARD-AI (Standards for Reporting Diagnostic Accuracy Studies–Artificial Intelligence) [2].
- Studies aimed at developing, updating, or validating multivariable diagnostic prediction models were evaluated using TRIPOD+AI (Transparent Reporting of a Multivariable Prediction Model for Individual Prognosis or Diagnosis + Artificial Intelligence [3]), regardless of whether the computational approach was based on conventional regression, machine learning, or deep learning.
- Preclinical studies employing animal models were assessed using ARRIVE 2.0 (Animal Research: Reporting of In Vivo Experiments [4]), specifically the "Essential 10" guidelines, which include experimental design, sample size, inclusion/exclusion criteria, randomization, blinding, outcome variables, statistical methods, animal characteristics, experimental procedures, and results presentation.

These tools were used exclusively as checklists to evaluate reporting quality and transparency. None of their elements were employed to generate aggregate scores or to classify risk of bias.

2. Assessment of Risk of Bias and Applicability

Risk-of-bias and applicability concerns were evaluated separately from reporting completeness.

- Diagnostic accuracy studies comparing a SERS-based index test with an appropriate target condition definition were assessed using QUADAS-2 (Quality Assessment of Diagnostic Accuracy Studies-2 [5]). For studies incorporating AI algorithms, methodological principles from QUADAS-AI [2] were additionally considered. Domains included the participants, index test, reference standard, and study flow/timing. Applicability concerns were analyzed for the participants, index test, and target condition. When a single study reported multiple biomarkers, thresholds, models, or diagnostic configurations, each relevant diagnostic estimate was evaluated independently.
- Prediction model studies were assessed using PROBAST (prediction model risk-of-bias assessment tool [6]), examining domains related to participants/data sources, predictors, outcomes, and statistical analysis. Risk-of-bias and applicability judgments were supported by textual justifications derived from authors' reported information.

No global quality scores were calculated due to the high heterogeneity of designs, experimental objectives, nanotechnology platforms, and application contexts, in line with methodological recommendations for systematic reviews of complex diagnostic technologies.

3. Assessment of Analytical and Technical Validation

Studies focused exclusively on SERS platform development, physicochemical characterization of nanoarchitectures, experimental biosensors, in vitro analytical validation, cell line discrimination, or preclinical investigations were not evaluated using clinical risk-of-bias tools when they lacked the methodological structure of diagnostic or predictive studies.

Instead, a specific analytical and technical validation framework was applied, derived from the IUPAC harmonized guidelines for single-laboratory validation of analytical methods [7] and the principles established by ISO 5725-1:2023 on accuracy, trueness, precision, repeatability, and reproducibility of measurement methods [8].

The evaluation included domains such as: physicochemical characterization of the nanoarchitecture, composition, morphology, and functionalization of the SERS substrate; description and calculation of the EF; calibration model and analytical range; LOD and limit of quantification (LOQ); analytical selectivity; interference and matrix effects; accuracy and recovery; spatial uniformity of the substrate; intra-assay repeatability; reproducibility across batches, operators, instruments, or days; temporal stability of the substrate and Raman signal; operational robustness; scalability; and manufacturing feasibility.

Additionally, parameters specific to SERS platforms that may influence analytical performance and reproducibility were documented, including: density and distribution of plasmonic hotspots, spectral response uniformity, intra- and inter-substrate variability, stability of Raman reporters, variability associated with nanoparticle aggregation phenomena, effects of analyte spatial distribution, and influence of the coffee-ring effect in liquid samples. These characteristics were analyzed descriptively and not treated as formal validation criteria unless explicitly supported by international standards or guidelines.

biosensors

MDPI

Table S1. Comparison of previous reviews and the present review on SERS-based breast cancer detection.

Review	Primary scope	Specific to BC	SERS central	Nanoarchitecture treated as functional variable	Spectral analysis / ML	Validation and reproducibility	Bibliometric analysis	Main distinction from present review
[9]	SERS biosensors for breast cancer on-comiRNAs	Yes	Yes	Partially; sensor designs described mainly by on-comiRNA application	Limited	Challenges noted; no structured evidence-quality framework	No	Restricted mainly to on-comiRNAs; no bibliometric or integrated clinical-readiness assessment
[10]	SERS detection of liquid-biopsy biomarkers in breast cancer	Yes	Yes	Partially; platforms organized primarily by biomarker class	Yes	Standardization and isolation challenges discussed; no formal bias framework	No	Liquid-biopsy focus; no bibliometric mapping or systematic architecture-to-translation framework
[11]	Current and developing liquid-biopsy techniques for breast cancer	Yes	No; one of several techniques	No	Not SERS-specific	Clinical trials and assay discordance discussed for liquid biopsy broadly	No	SERS is a secondary detection option
[12]	Plasmonic nanoparticles for early breast cancer biosensors	Yes	No; one of several mechanisms	Partially	No dedicated SERS spectral-analysis synthesis	Safety and biocompatibility noted	No	Broader plasmonic-biosensor scope; not exclusive to SERS
[13]	SERS sensors for diseases, biomolecules and machine learning	No	Yes	Partially	Dedicated ML coverage	Reproducibility discussed broadly	No	Strong ML focus but not breast cancer-specific

Review	Primary scope	Specific to BC	SERS central	Nanoarchitecture treated as functional variable	Spectral analysis / ML	Validation and reproducibility	Bibliometric analysis	Main distinction from present review
[14]	SERS cancer biomarkers and technological maturity	No	Yes	Yes, across several cancers	Yes	Broad technological-maturity and translation discussion	No	Closest translational comparator, but not breast cancer-specific or bibliometric
[15]	Gold-nanostar SERS nanotags for bioimaging	No	Yes	Yes, limited to nanostars	Electromagnetic simulation, not classification	Batch heterogeneity and biodistribution discussed	No	Single nanoarchitecture family and predominantly preclinical imaging
[16]	DNA-assembled plasmonic nanoarchitectures for biomedical applications	No	Yes, alongside LSPR	Yes, limited to DNA assemblies	Calibration; no diagnostic-classification synthesis	Yield and field-control challenges discussed	No	Architecture-specific but not breast cancer-specific
This review	SERS-based breast cancer detection across biomarkers, samples and platforms	Yes	Yes	Yes; architecture linked to hotspots, analytical function, reproducibility and manufacturability	Yes; includes bias and pseudoreplication considerations	Structured evaluation of model/sample level, external validation, reproducibility and translational readiness	Yes	Integrates bibliometric mapping, functional nanoarchitecture analysis and evidence/translation appraisal

References

1. EQUATOR Network – Enhancing the QUAlity and Transparency Of Health Research. Available online: http://www.equator-network.org/ (accessed on 3 September 2026).
2. Sounderajah, V.; Guni, A.; Liu, X.; Collins, G.S.; Karthikesalingam, A.; Markar, S.R.; Golub, R.M.; Denniston, A.K.; Shetty, S.; Moher, D.; et al. The STARD-AI reporting guideline for diagnostic accuracy studies using artificial intelligence. *Nat. Med.* **2025**, *31*, 3283–3289, https://doi.org/10.1038/s41591-025-03953-8.
3. Collins, G.S.; Moons, K.G.M.; Dhiman, P.; Riley, R.D.; Beam, A.L.; Van Calster, B.; Ghassemi, M.; Liu, X.; Reitsma, J.B.; van Smeden, M.; et al. TRIPOD+AI statement: updated guidance for reporting clinical prediction models that use regression or machine learning methods. *BMJ* **2024**, *385*, e078378, https://doi.org/10.1136/bmj-2023-078378.
4. du Sert, N.P.; Hurst, V.; Ahluwalia, A.; Alam, S.; Avey, M.T.; Baker, M.; Browne, W.J.; Clark, A.; Cuthill, I.C.; Dirnagl, U.; et al. The ARRIVE guidelines 2.0: Updated guidelines for reporting animal research. *PLoS Biol.* **2020**, *18*, e3000410, https://doi.org/10.1371/journal.pbio.3000410.
5. Whiting, P.F.; Rutjes, A.W.S.; Westwood, M.E.; Mallett, S.; Deeks, J.J.; Reitsma, J.B.; Leeflang, M.M.G.; Sterne, J.A.C.; Bossuyt, P.M.M.; QUADAS-2 Group. QUADAS-2: A Revised Tool for the Quality Assessment of Diagnostic Accuracy Studies. *Ann. Intern. Med.* **2011**, *155*, 529–536. doi: 10.7326/0003-4819-155-8-201110180-00009.
6. Wolff, R.F.; Moons, K.G.; Riley, R.; Whiting, P.F.; Westwood, M.; Collins, G.S.; Reitsma, J.B.; Kleijnen, J.; Mallett, S.; for the PROBAST Group. PROBAST: A Tool to Assess the Risk of Bias and Applicability of Prediction Model Studies. *Ann. Intern. Med.* **2019**, *170*, 51–58, doi:10.7326/m18-1376.
7. Thompson, M.; Ellison, S.L.R.; Wood, R. Harmonized guidelines for single-laboratory validation of methods of analysis (IUPAC Technical Report). *Pure Appl. Chem.* **2002**, *74*, 835–855, https://doi.org/10.1351/pac200274050835.
8. ISO 5725-1:2023; Accuracy (Trueness and Precision) of Measurement Methods and Results – Part 1: General Principles and Definitions. International Organization for Standardization (ISO) ISO: Geneva, Switzerland, 2023.
9. Chauhan, P.; Bhargava, A.; Kumari, R.; Ratre, P.; Tiwari, R.; Kumar Srivastava, R.; Yu. Goryacheva, I.; Kumar Mishra, P. Surface-Enhanced Raman Scattering Biosensors for Detection of oncomiRs in Breast Cancer. *Drug Discovery Today* **2022**, *27*, 2121–2136, doi:10.1016/j.drudis.2022.04.016
10. Liu, C.; Yang, Y.; Wu, Y. Recent Advances in Exosomal Protein Detection Via Liquid Biopsy Biosensors for Cancer Screening, Diagnosis, and Prognosis. *AAPS J* **2018**, *20*, 41, doi:10.1208/s12248-018-0201-1.
11. Wu, H.-J.; Chu, P.-Y. Current and Developing Liquid Biopsy Techniques for Breast Cancer. *Cancers* **2022**, *14*, 2052, doi:10.3390/cancers14092052.
12. Saadh, M.J.; Saeed, T.N.; Alfarttoosi, K.H.; Sanghvi, G.; Roopashree, R.; Thakur, V.; Lakshmi, L.; Aminov, Z.; Taher, W.M.; Alwan, M.; et al. Plasmonic Nanoparticles: Enhancing Early Breast Cancer Detection Through Biosensors. *Plasmonics* **2025**, *20*, 8067–8084, doi:10.1007/s11468-025-02864-1.
13. Beeram, R.; Vepa, K.R.; Soma, V.R. Recent Trends in SERS-Based Plasmonic Sensors for Disease Diagnostics, Biomolecules Detection, and Machine Learning Techniques. *Biosensors* **2023**, *13*, 328, doi:10.3390/bios13030328.
14. Vázquez-Iglesias, L.; Stanfoca Casagrande, G.M.; García-Lojo, D.; Ferro Leal, L.; Ngo, T.A.; Pérez-Juste, J.; Reis, R.M.; Kant, K.; Pastoriza-Santos, I. SERS Sensing for Cancer Biomarker: Approaches and Directions. *Bioactive Materials* **2024**, *34*, 248–268, doi:10.1016/j.bioactmat.2023.12.018.
15. Patel, M.T.; Goldberg Oppenheimer, P.G. Advancements in Cancer Diagnostics: Integrating Surface-Enhanced Raman Spectroscopy and Microptofluidics for Precision and Versatility. *Applied Spectroscopy Reviews* **2025**, *60*, 511–554, doi:10.1080/05704928.2025.2465394.
16. Song, S.; Jeon, M.J.; Lee, J.U.; Sim, S.J. Recent Advances in DNA-Assembled Plasmonic Nanoarchitectures for Biomedical Applications. *TrAC Trends in Analytical Chemistry* **2024**, *177*, 117784, doi:10.1016/j.trac.2024.117784.
